# A Scoping Review of Instruments Used to Measure Resilience in Samples of Nurses

**DOI:** 10.1111/jan.16769

**Published:** 2025-02-07

**Authors:** Alannah L. Cooper, Georgia Roberts, Desley G. Hegney, Janie A. Brown

**Affiliations:** ^1^ St John of God Subiaco Hospital Subiaco Western Australia Australia; ^2^ School of Nursing, College of Health & Education Murdoch University Murdoch Western Australia Australia; ^3^ Nursing School University of Adelaide Adelaide South Australia Australia; ^4^ School of Nursing Curtin University Bentley Western Australia Australia; ^5^ St John of God Midland Public and Private Hospital Midland Western Australia Australia; ^6^ The Western Australian Group for Evidence Informed Healthcare Practice Curtin University Bentley Western Australia Australia

**Keywords:** adversity, instrument, measure, nurse, resilience, scale, stress, tool

## Abstract

**Aim:**

To identify and critically appraise instruments that have been used to measure nurse resilience.

**Design:**

A scoping review.

**Data Sources:**

Comprehensive literature searches were conducted using four electronic databases CINAHL Ultimate, MEDLINE, PsycINFO and Emcare from the year 2012 to December 2024.

**Methods:**

The titles, then abstracts, of retrieved articles were screened by the authors against inclusion and exclusion criteria, then full‐text screening was performed using Rayyan. Data about the study characteristics and the instruments used to measure nurse resilience were extracted. Copies of the instruments used to measure resilience were obtained and appraised.

**Results:**

Of the *n* = 4694 publications identified in the initial search *n* = 386 were included in the scoping review. Studies originated in *n* = 45 countries, the majority were conducted in China (*n* = 119) and the United States of America (*n* = 53). Across the *n* = 386 included studies, *n* = 15 instruments to measure resilience were identified and critically appraised. The scores for the instruments critically appraised ranged from 0 to 6 out of a total possible score of 11. Synthesis of results examined instrument development, instrument features and application of instruments.

**Conclusion:**

Critical appraisal of the instruments used to measure nurse resilience revealed significant deficiencies. None of the instruments included all of the key attributes and factors that influence nurse resilience. There was a predominant focus on individual factors and little consideration of the influence of nursing work environments. Due to the shortcomings of the existing instruments, there are currently substantial limitations in our understanding of nurse resilience and how to measure it.

**Implications for the Profession:**

A profession‐specific comprehensive measure of nurse resilience needs to be developed to better capture the attributes of nurse resilience.

**Impact:**

This review highlights the limitations of instruments applied to measure nurse resilience.

**Reporting Method:**

The JBI scoping review framework.

**Patient or Public Contribution:**

No patient or public contribution.

**Summary**

- What does this paper contribute to the wider global clinical community?
  ○ Nurse resilience has been predominately investigated in cross‐sectional studies of hospital‐based nurses.
  ○ The instruments that have been used to measure resilience in samples of nurses are generally weak and do not feature all of the known attributes and factors that influence nurse resilience.
  ○ The impact of the work environment should be considered when measuring nurse resilience.

## 1 Introduction

Globally, nursing shortages continue to increase (Buchan and Catton 2023). This longstanding trend has been exacerbated by the coronavirus pandemic adding pressure to already struggling healthcare systems (Buchan, Catton, and Shaffer 2022). Nurses working in challenging conditions can experience negative impacts on their psychological well‐being (Hegney et al. 2015; Lorber and Dobnik 2022; Woo et al. 2020) which can impact the delivery of patient care (Aiken et al. 2023; Weaver et al. 2018). For example, burnout, which is one factor in resilience in nurses is associated with reduced patient safety and adverse events, including patient falls, medication errors and infections (Dall'Ora et al. 2020). In an attempt to promote patient safety and understand adverse psychological outcomes for nurses, protective factors such as resilience have been examined in research and practice (Mealer et al. 2012; Rees et al. 2015). The prevalence of research investigating nurse resilience has increased in recent years due to the urgent need to find ways to sustain and retain nurses in the profession (Kim and Chang 2022).

To investigate resilience in the context of nurses, researchers have sought to measure resilience with a variety of instruments (Cooper et al. 2020; Windle, Bennett, and Noyes 2011). Since early studies with a focus on nurse resilience were conducted (Dolan, Strodl, and Hamernik 2012; Manzano García and Ayala Calvo 2012; Mealer et al. 2012), our understanding of nurse resilience has evolved. This broader understanding of resilience is in keeping with psychological perspectives, where resilience is acknowledged to be variable based on the range of factors including time, context, age and the life circumstances individuals are exposed to (Connor and Davidson 2003). The majority of research investigating resilience has focused on the individual, and in practice, organisations have predominately looked at what individuals can do to maintain their own resilience whilst being exposed to the same work conditions (Cooper, Brown, and Leslie 2021b). This focus on the individual has come under criticism as it fails to examine and address factors in the work environment that undermine nurse resilience (Taylor 2019; Virkstis, Herleth, and Langr 2018). However, few research studies have investigated the external factors that influence nurse resilience (Cooper, Brown, and Leslie 2021b; Gensimore et al. 2020; Tabakakis et al. 2019).

A concept analysis (Cooper et al. 2020) and integrative review of nurse resilience (Cooper, Brown, and Leslie 2021b) highlighted the lack of consideration of external factors that can affect nurse resilience, and the consequent underestimation of the complexity of nurse resilience and the responsibility organisations have to their employees. Key attributes of nurse resilience were identified from the literature and a working definition was arrived at from the concept analysis (Cooper et al. 2020). The key attributes of nurse resilience derived from the literature were; (1) social support, (2) self‐efficacy, (3) work‐life balance (4) self‐care, (5) humour, (6) optimism and (7) being realistic. A limitation of the definition developed in the concept analysis was that it drew from the literature that had predominately focused on nurses maintaining their own resilience. To address this gap, an updated definition of nurse resilience was subsequently developed that derived from qualitative data obtained via focus groups with nurses. Data were analysed thematically, following the process described by Braun and Clarke (2006). This analysis revealed broader factors known to impact resilience in nurses. As a consequence of this focus group study, a further four attributes of nurse resilience were identified – workplace conditions, organisational philosophy, management performance and team factors – and a revised definition was published.

Resilience is a complex and dynamic process, influenced by individual factors, as well as modifiable workplace conditions, organizational philosophy, management performance, and the teams nurses work within. These factors influence the extent to which resilience can be sustained, to enable nurses to positively adapt to workplace stressors, avoid psychological harm, and continue to provide safe, high‐quality patient care (Cooper, Leslie, and Brown 2022, p. 9)

Given the urgent need to retain nurses in the profession and to ensure the highest standard of patient safety, establishing how well existing instruments capture the individual (Cooper et al. 2020) and organisational factors (Cooper, Leslie, and Brown 2022) known to influence nurse resilience is vital. The use of instruments that do not measure the known attributes of nurse resilience may be under‐ or over‐estimating levels of nurse resilience, contributing to the development of suboptimal interventions or strategies to promote nurse resilience, resulting in missed opportunities to better support nurses in their work.

## 2 The Review

### 2.1 Aim

The aim of this scoping review was to identify and critically appraise instruments that have been used to measure nurse resilience. The following question was developed to guide the review: How has resilience been measured in samples of nurses? The objectives of the review were to:

1. Identify all instruments that have been used to measure resilience in samples of nurses.
2. Extract information on the types of research studies that administered measures of resilience in samples of nurses.
3. Assess the features of each instrument used to measure resilience in nurses.
4. Ascertain how many of the key attributes (Cooper et al. 2020) and known factors that influence nurse resilience (Cooper, Leslie, and Brown 2022) are present in each instrument.

## 3 Methods

### 3.1 Design

A scoping review was conducted following the JBI scoping review framework (Peters et al. 2020) to identify and critically appraise instruments used to measure resilience in nurses (File S1). The JBI framework (Peters et al. 2020) consists of nine steps: (1) defining and aligning the research question and objectives; (2) developing and aligning the inclusion criteria with the research question and objectives; (3) describing the planned approach for searching, selecting, extracting and presenting the evidence; (4) searching the evidence; (5) selecting the evidence; (6) extracting the evidence; (7) analysing the evidence; (8) presenting the results and (9) summarising the evidence and drawing conclusions. The review is presented in accordance with the Preferred Reporting Items for Systematic Review and Meta‐Analyses Extension for Scoping Reviews (PRISMA‐ScR) (Tricco et al. 2018).

### 3.2 Search Methods

The search strategy was developed by two authors (AC and GR) and reviewed by an academic librarian. In a preliminary search of the electronic database CINAHL Ultimate, key search terms were nurs* AND resilienc* AND measure OR tool OR scale OR instrument. Results were analysed for the detection of further key words and seed articles (Cooper, Brown, and Leslie 2021a; Mealer et al. 2012) were utilised to ensure all relevant articles were included. During preliminary searching, the key search terms cross‐sectional OR longitudinal were added. An exclusion search term of NOT qualitative was added to minimise the number of ineligible articles retrieved. The strategy identified key search terms that were then applied across four electronic databases; CINAHL Ultimate, MEDLINE, PsycINFO and Emcare (File S2). Search limits applied were articles published between the year 2012 and December 2024, full text and English language. The date limit of 2012 was applied to facilitate a timely review and with reference to this being a known time when a number of research studies (Dolan, Strodl, and Hamernik 2012; Manzano García and Ayala Calvo 2012; Mealer et al. 2012) quantitatively investigating resilience in nurses were emerging. Final database searches were conducted on the 9th of December 2024. All retrieved articles were uploaded to Endnote where duplicates were removed. The remaining articles were then transferred to Rayyan (Ouzzani et al. 2016).

### 3.3 Inclusion and Exclusion Criteria

Eligibility criteria were developed based on the JBI scoping review methodology (Peters et al. 2020) which includes Participants, Concept and Context. For this review: (1) Participants were nurses; (2) Concept was instruments used to measure resilience in samples of nurses and (3) Context was all healthcare settings. Inclusion criteria captured quantitative and mixed methods research studies that used an instrument to measure resilience in a sample of nurses working in any healthcare setting. Studies of Registered Nurses, Enrolled Nurses, Licensed Practice Nurses, Nurse Practitioners, Nurse Managers and all equivalents met inclusion criteria. The exclusion criteria were qualitative research studies and study samples that included student nurses, assistants in nursing, midwives, nurses working in academic settings or other health professionals. These exclusion criteria were applied to keep the review profession specific and relevant to clinical healthcare settings.

### 3.4 Selection of Sources

Following the removal of duplicates, a pilot of source selection was undertaken by the two authors (AC and GR) completing the screening process. A random sample of 25 titles and abstracts were reviewed. No modifications to the inclusion or exclusion criteria were required on completion of the pilot. A three‐stage review process was then undertaken. In the first stage, two researchers (AC and GR) independently screened the titles and abstracts to determine potential eligibility and relevance to the review. In the second stage, full texts were then independently reviewed and assessed (AC and GR) against the inclusion criteria. After the first and second stage reviews, conflicts on screening decisions were discussed and a final consensus was reached through discussion by three authors (AC, GR and JB). In the final stage of the review, copies of the instruments used to measure resilience in the publications were sought.

### 3.5 Data Extraction

Data were extracted for all research studies that met the inclusion criteria. The plan for data extraction was discussed by two authors (AC and GR) with reference to the second objective of the scoping review. The following data were extracted from each study; citation, year of publication, country/countries, study design, sample size, setting, resilience instrument used and other measures obtained.

### 3.6 Quality Appraisal

Critical appraisal is not a requirement of a scoping review (Pollock et al. 2022). Typically, critical appraisal involves evaluating the methodological rigour of the research studies included in a literature review (Tod, Booth, and Smith 2022). However, as the focus of this review was to identify and examine instruments that have been used to measure resilience in samples of nurses, a specific appraisal tool was developed to assess the instruments. Each instrument was scored based on the key attributes and known factors that influence nurse resilience (Cooper, Leslie, and Brown 2022) with a total possible score of 11 (Table 1). Based on Cooper, Leslie, and Brown (2022), the 11 key attributes and known factors that influence nurse resilience are; social support, self‐efficacy, work‐life balance, self‐care, humour, optimism, being realistic, workplace conditions, organisational philosophy, management performance and team factors. A comparison was made between what the original authors contended that each instrument measured, with the 11 key attributes and known factors that influence nurse resilience (Cooper et al. 2020; Cooper, Leslie, and Brown 2022). The working definitions (Cooper et al. 2020; Cooper, Leslie, and Brown 2022) for each key attribute of nurse resilience were used to determine if an instrument measured the construct (Table 1). Two authors (JB and GR) independently reviewed and scored (0 absent, 1 present) each included instrument based on the criteria outlined in Table 1. The scores were then compared and discussed by all of the research team until a consensus was reached about the scoring for each instrument assessed.

**TABLE 1:** Working definitions of the key attributes of nurse resilience.

Key attribute	Working definition	Score
Social support	“Nurses can draw social support from colleagues, managers, friends and family. Individuals need to engage with social supports, and workplaces can provide support systems and foster positive collegial relationships.” (Cooper et al. 2020) “the provision of assistance or comfort to others, typically to help them cope with biological, psychological, and social stressors. Support may arise from any interpersonal relationship in an individual's social network, involving family members, friends, neighbors, religious institutions, colleagues, caregivers, or support groups. It may take the form of practical help (e.g., doing chores, offering advice), tangible support that involves giving money or other direct material assistance, and emotional support that allows the individual to feel valued, accepted, and understood” (APA dictionary of psychology) (APA Dictionary of Psychology)	1
Self‐efficacy	An individual's belief in their own ability to succeed. (Cooper et al. 2020) “An individual's subjective perception of their capability to perform in a given setting or to attain desired results” (APA dictionary of psychology) (APA Dictionary of Psychology)	1
Work–life balance	“Work–life balance is the division of an individual's time between work and family or leisure activities.” Time does not need to be equally divided between work and leisure but rather work‐life balance is met when there is a compatibility between work and non‐work activities. (Cooper et al. 2020) “The level of involvement between the multiple roles in a person's life, particularly as they pertain to employment and family or leisure activities” (APA dictionary of psychology) (APA Dictionary of Psychology)	1
Self‐care	“When an individual actively practices protecting their well‐being and happiness. This encompasses … both physical and mental well‐being” (Cooper et al. 2020)	1
Humour	“The ability to make light of adversity” (Cooper et al. 2020) “The ability to find things funny, the way in which people see that some things are funny, or the quality of being funny” (Cambridge dictionary) (HUMOUR | English meaning ‐ Cambridge Dictionary)	1
Optimism	“Optimism is the extent to which individuals hold favourable expectations for the future” (Cooper et al. 2020) “In nurses, optimism is often discussed in the context of remaining positive and looking for the positive in adversity” (Cooper et al. 2020)	1
Being realistic	“Being realistic can be described as having a practical and sensible idea of what can be achieved or expected. This includes reframing experiences, having realistic expectations about caregiving, cultivating a realistic perspective on life and realistic goal setting” (Cooper et al. 2020)	1
Workplace conditions	Perceptions of management of the hospital and organisational support offered. Physical resources available to staff, leave and pay conditions, and support services all contribute to workplace conditions (Cooper, Leslie, and Brown 2022)	1
Organisational philosophy	Does the workplace uphold the organisational values and philosophy at the organisational level? Does the organisation “live” the values it sets and is this evident in working conditions? (Cooper, Leslie, and Brown 2022)	1
Management performance	Management performance and perceptions of management impact nursing resilience. Leadership skills and level of support provided by management included (Cooper, Leslie, and Brown 2022)	1
Team factors	Camaraderie can increase resilience levels in nursing. Team and colleague support and dealing with adversity as a unit rather than an individual can assist nurses to cope with challenges and improve resilience (Cooper, Leslie, and Brown 2022)	1

### 3.7 Synthesis of Results

A descriptive summary of the review findings is presented through narrative, tables and figures. In keeping with the aim and objectives of the review, data were extracted and presented to describe the characteristics of studies that have measured resilience in samples of nurses.

## 4 Results

### 4.1 Selection of Sources of Evidence

**FIGURE 1:**
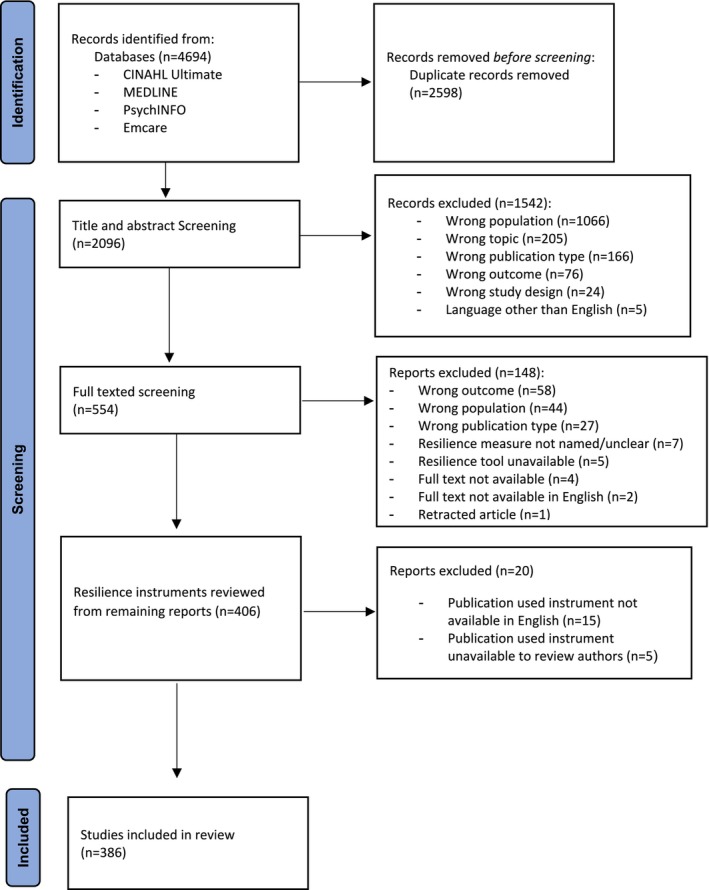
PRISMA flow diagram.

The initial search identified *n* = 4694 publications. Of these, *n* = 2598 were duplicates, leaving *n* = 2096 for possible inclusion (Figure 1). The remaining publications underwent title and abstract screening and *n* = 1542 were excluded. This left *n* = 554 publications for full‐text review, where a further *n* = 148 publications were excluded, leaving *n* = 406 publications. The resilience instruments for these *n* = 406 publications were then reviewed. There were *n* = 5 studies that used an instrument that was not published in the literature and not available following requests from the authors, and *n* = 15 where the instrument was not available in English. Following these exclusions, *n* = 386 publications met the inclusion criteria.

### 4.2 Characteristics of Sources of Evidence

**FIGURE 2:**
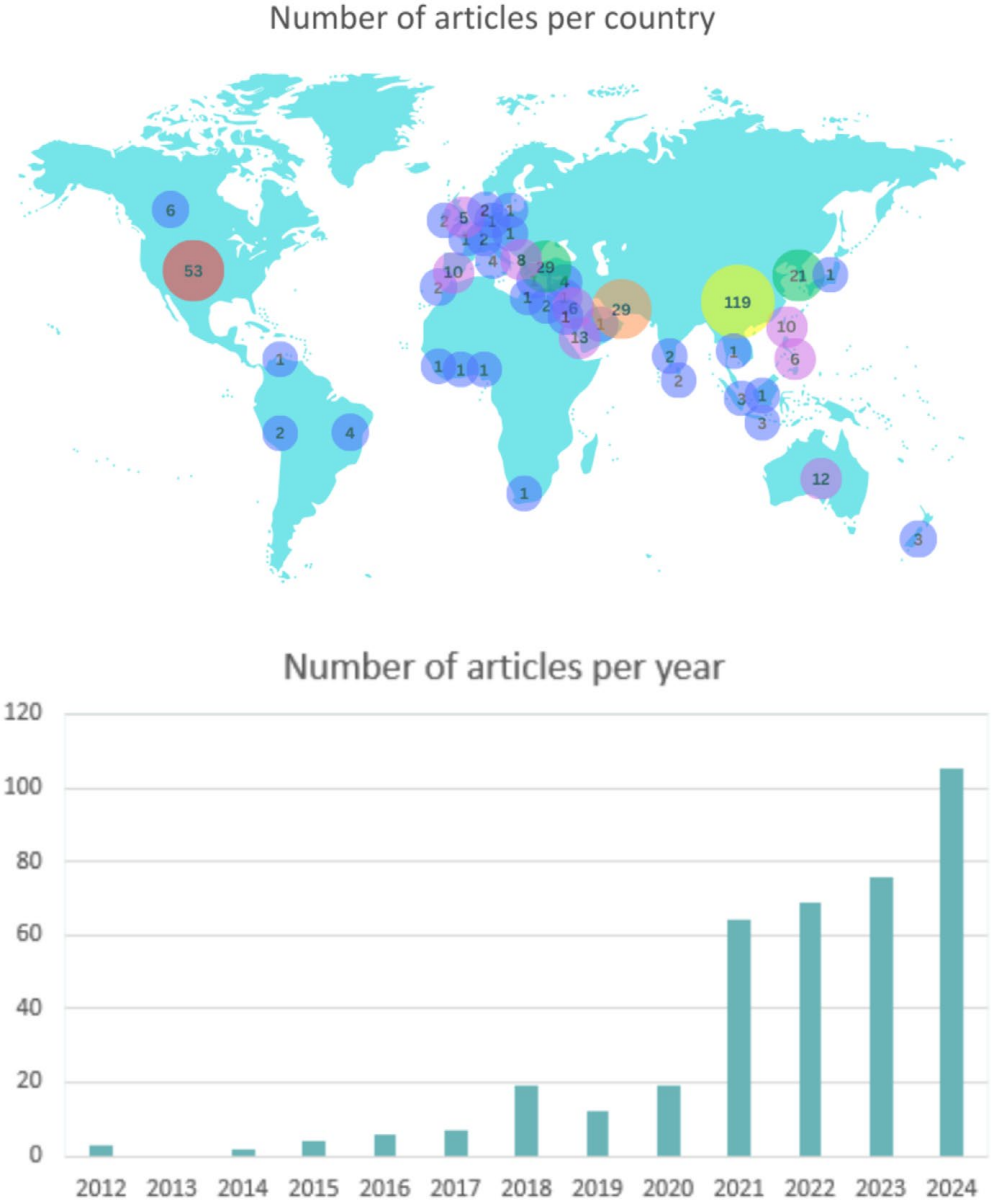
Number of articles measuring resilience in nurses by country and year.

The prevalence of studies that measured resilience in samples of nurses increased from *n* = 3 studies published in 2012 to *n* = 105 in 2024 (Figure 2). The majority of studies (*n* = 329, 85%) had a cross‐sectional design (Table 2 File S3). Only 47 studies had an interventional design. Of these *n* = 30 consisted of interventions that aimed to promote nurse resilience. Studies originated in *n* = 45 countries; the majority were conducted in China (*n* = 119) and the United States of America (*n* = 53) (Table 2). Study samples predominately consisted of hospital‐based nurses, working in all specialities. Sample sizes ranged from seven to 70,932 participants. A number of studies focused on specific specialities, with intensive care (*n* = 23), mental health settings (*n* = 23) and emergency departments (*n* = 16) being the most frequently investigated.

**TABLE 2:** Summary of included studies.

Author and year	Country	Study design	Sample size	Setting	Resilience tool/measure used	Other attributes measured/tools used
Abdollahi et al. (2021)	Iran	Cross‐sectional	422	Multisite hospital	Connor‐Davidson Resilience Scale 25‐item	Professional moral courage
Abdulmohdi (2024)	United Kingdom	Cross‐sectional	294	Multisite hospital and community	Connor‐Davidson Resilience Scale 10 item	Burnout, perceived social support and perceived organisational support
Abu‐Alhaija and Gillespie (2022)	America	Cross‐sectional	48	Multisite hospital trauma EDs	Connor‐Davidson Resilience Scale 25‐item	Clinical events
Abualruz and Hayajneh (2023)	Jordan	Quasi‐experimental	150	Single site hospital	Brief Resilience Scale	Depression, anxiety and stress
Abualruz et al. (2024a)	Jordan	Cross‐sectional	155	Multisite hospital	Brief Resilience Scale	Depression, anxiety, stress, emotional intelligence
Abualruz et al. (2024b)	Saudi Arabia	Cross‐sectional	150	Single site hospital	Resilience Scale 14‐item	Emotional intelligence, work engagement and psychological empowerment
Afshari et al. (2021)	Iran	Cross‐sectional	387	Multisite hospital	Connor‐Davidson Resilience Scale 25‐item	Nil
Aghamohammadi et al. (2023)	Iran	Cross‐sectional	1025	Multisite hospital	Connor‐Davidson Resilience Scale 25‐item (Persian translation)	Nil
Akbulut et al. (2023)	Turkey	Cross‐sectional	676	Single site hospital	Brief Resilience Scale	Vaccination hesitancy and anxiety
Akinabadi et al. (2024)	Iran	Cross‐sectional	205	Single site hospital	Connor‐Davidson Resilience Scale 25‐item	Family function and lifestyle
Alameddine et al. (2021a)	Lebanon	Cross‐sectional	265	Single site hospital	Connor‐Davidson Resilience Scale 25‐item	Job satisfaction
Alameddine et al. (2021b)	Lebanon	Cross‐sectional	511	Multisite hospital	Connor‐Davidson Resilience Scale 25‐item	Intention to quit and burnout
Alan et al. (2022)	Turkey	Cross‐sectional	489	Multisite hospital	Connor‐Davidson Resilience Scale 25‐item (Turkish translation)	Critical thinking, diagnostic skills and communication skills
Albaqawi et al. (2024)	Saudi Arabia	Cross‐sectional	511	Multisite hospital	Connor‐Davidson Resilience Scale 10 item	Compassion fatigue, moral injury and moral distress
Albougami (2024)	Saudi Arabia	Cross‐sectional	216	Single site hospital	Brief Resilient Coping Scale	Nil
Alenezi (2024)	Saudi Arabia	Cross‐sectional	361	Single site mental health setting	Resilience at Work Scale	Workplace violence
Al Hadid et al. (2022)	Jordan	Cross‐sectional	300	Multisite hospital	Connor‐Davidson Resilience Scale 25‐item	Perceived stress
Alharbi et al. (2020)	Saudi Arabia	Cross‐sectional	321	Multisite hospital critical care settings	Connor‐Davidson Resilience Scale 25‐item	Coping strategies, compassion satisfaction and compassion fatigue
Al‐Harrasi et al. (2024)	Oman	Cross‐sectional	173	Multisite hospital	Brief Resilience Scale	Burnout
Alhawatmeh et al. (2021)	Jordan	Cross‐sectional	550	Multisite hospital	Connor‐Davidson Resilience Scale 25‐item	Perceived stress, physical health, psychological health and social health
Almegewly et al. (2022)	Saudi Arabia	Cross‐sectional	139	Single site hospital critical care settings	Connor‐Davidson Resilience Scale 10‐item	Perceived stress
Alonazi et al. (2023)	Saudi Arabia	Cross‐sectional	179	Single site hospital mental health settings	Connor‐Davidson Resilience Scale 25‐item	Compassion fatigue, compassion satisfaction, burnout and secondary post‐traumatic stress
Al‐Shomrani et al. (2024)	Saudi Arabia	Cross‐sectional	318	Multisite hospital	Connor‐Davidson Resilience Scale 25‐item	Turnover intention
Andersen et al. (2021)	America	Cross‐sectional	167	Multisite hospital	Connor‐Davidson Resilience Scale 10‐item	Non‐work‐related stress and shift work characteristics
Ang et al. (2018a)	Singapore	Cross‐sectional	1338	Multisite hospital	Connor‐Davidson Resilience Scale 10‐item	Burnout, compassion fatigue and secondary traumatic stress
Ang et al. (2018b)	Singapore	Cross‐sectional	1338	Multisite hospital	Connor‐Davidson Resilience Scale 10‐item	Nil
Aqtam et al. (2023)	Iran	Cross‐sectional	227	Multisite hospital intensive care units	Brief Resilient Coping Scale	Stress
Asadi et al. (2023)	Iran	Cross‐sectional	340	Single site hospital	Connor‐Davidson Resilience Scale 25‐item	Professional ethics
Ata et al. (2020)	Turkey	Cross‐sectional	100	Single site hospital intensive care unit	Resilience Scale for Adults	Compassion fatigue, compassion satisfaction, burnout and secondary post‐traumatic stress
Atay et al. (2023)	Turkey	Cross‐sectional	263	Multisite hospital	Connor‐Davidson Resilience Scale 25‐item (Turkish translation)	Post‐traumatic Growth
Babanataj et al. (2019)	Iran	Quasi‐experimental	30	Single site hospital critical care settings	Connor‐Davidson Resilience Scale 25‐item	Stress
Bai and Bai (2024)	China	Cross‐sectional	417	Single site hospital	Connor‐Davidson Resilience Scale 10‐item (Chinese translation)	Personal strength, psychological needs satisfaction and job satisfaction
Bai et al. (2024)	China	Cross‐sectional	1169	Multisite hospital	Connor‐Davidson Resilience Scale 10‐item	Gratitude, stress and job satisfaction
Bani et al. (2023)	Italy	Cross‐sectional	102	Multisite hospital	Connor‐Davidson Resilience Scale 10‐item (Italian translation)	Mental well‐being and burnout
Bernburg et al. (2019)	Germany	Randomised controlled trial	86	Multisite hospital mental health settings	Brief Resilient Coping Scale	Job stress, emotional regulation skills and quality of relationships with patients
Bonamer and Aquino‐Russell (2019)	America	Quasi‐experimental	27	Single site community	Connor‐Davidson Resilience Scale 25‐item	Burnout, compassion satisfaction and compassion fatigue
Bouchard and Rainbow (2021)	America	Cross‐sectional	118	Nurse Practitioners completing a Doctor of Nursing Practice program	Response to Stressful Experiences Scales	Adverse childhood experiences, job‐stress, compassion fatigue, compassion satisfaction, burnout and secondary post‐traumatic stress
Brook et al. (2023)	United Kingdom	Longitudinal	68	Multisite hospital and community	Brief Resilience Scale	Post‐traumatic stress disorder, burnout, depression, anxiety, stress, well‐being and nursing work index
Brown et al. (2018)	America	Cross‐sectional	521	Multisite hospital	Connor‐Davidson Resilience Scale 10‐item	Change fatigue and job satisfaction
Buntoro et al. (2023)	Indonesia	Cross‐sectional	902	Online snowball sample	Adapted Adult Personal Resilience Scale	Depression level and work duration
Bursch et al. (2018)	America	Cross‐sectional	115	Single site hospital paediatric and neonatal intensive care	Brief Resilience Scale	Burnout, post‐traumatic stress, depression, anxiety and stress
Byers et al. (2021)	America	Cross‐sectional	77	Members of the National Black Nurses Association	Brief Resilience Scale	Experiences of racism and stress
Cabrera‐Aguilar et al. (2023)	Peru	Cross‐sectional	459	Single site hospital	Brief Resilient Coping Scale	Stress, self‐efficacy and work engagement
Cao and Chen (2019)	China	Cross‐sectional	345	Multisite hospital haemodialysis	Connor‐Davidson Resilience Scale 10‐item	Empathy and work engagement
Cao and Chen (2020)	China	Cross‐sectional	582	Multisite hospital haemodialysis	Connor‐Davidson Resilience Scale 10‐item	Empathy and work engagement
Cao and Chen (2021)	China	Cross‐sectional	528	Multisite hospital haemodialysis	Connor‐Davidson Resilience Scale 10‐item	Empathy, work engagement, compassion fatigue scale and turnover intention
Cao et al. (2021a)	China	Cross‐sectional	329	Multisite hospital	Connor‐Davidson Resilience Scale 10‐item	Transition shock, social support, work environment and turnover intention
Cao et al. (2021b)	China	Cross‐sectional	393	Multisite hospital	Connor‐Davidson Resilience Scale 10‐item	Transition shock scale, empathy, compassion fatigue, compassion satisfaction, burnout and secondary post‐traumatic stress
Cao et al. (2024)	China	Cross‐sectional	725	Multisite hospital	Connor‐Davidson Resilience Scale 10‐item	Occupational stress and insomnia
Caroccini et al. (2024)	Brazil	Cross‐sectional	164	Multisite hospital	Connor‐Davidson Resilience Scale 25‐item (Brazilian translation)	Meaning of work
Carpio et al. (2018)	America	Cross‐sectional	48	Multisite hospital	Resilience at Work Scale	Nil
Catarelli et al. (2023)	America	Cross‐sectional	43	Multisite hospital	Brief Resilience Scale	Burnout
Çelik and Yarali (2023)	Turkey	Randomised controlled trial	100	Single site hospital	Connor‐Davidson Resilience Scale 25‐item	Sleep quality
Cha and Baek (2023)	South Korea	Cross‐sectional	300	Multisite hospital	Connor‐Davidson Resilience Scale 25‐item (Korean translation)	Emotional labour, depression, job stress, Tae‐wom (workplace violence/organisational culture) coping and perceived threat of COVID‐19
Chan, S., et al. (2021)	China	Cross‐sectional	124	Multisite hospital, department of health, ‘others’	Connor‐Davidson Resilience Scale 10‐item	Anxiety, mental health, Covid‐19 pandemic‐related stress and coping ability
Chen et al. (2021)	China	Cross‐sectional	70,932	Multisite hospital	Connor‐Davidson Resilience Scale 25‐item (Chinese translation)	Work–family conflict and career development
Chen et al. (2022)	China	Cross‐sectional	413	Multisite hospital mental health settings	Connor‐Davidson Resilience Scale 25‐item (Chinese translation)	Occupational stress, and mental health and well‐being
Chen et al. (2023)	China	Cross‐sectional	160	Multisite care facilities for older adults	Connor‐Davidson Resilience Scale 25‐item (Chinese translation)	Self‐efficacy and attitudes about providing mouth care and work stress
Chen et at. (2024a)	China	Cross‐sectional	121	Multisite hospital	Connor‐Davidson Resilience Scale 25‐item (Chinese translation)	Challenge‐hindrance stressors and coping
Chen et al. (2024b)	China	Cross‐sectional	1141	Multisite hospital	Connor‐Davidson Resilience Scale 25‐item (Chinese translation)	Workplace violence, turnover intention and compassion fatigue
Chen et al. (2024c)	China	Cross‐sectional	241	Single site hospital	Connor‐Davidson Resilience Scale 25‐item (Chinese translation)	Job stress and emergency response competence
Chesak et al. (2015)	America	Randomised controlled trial	55	Single site hospital	Connor‐Davidson Resilience Scale 25‐item	Stress Scale, mindful attention awareness and anxiety
Chesak et al. (2021)	America	Quasi‐experimental	51	Single site hospital	Connor‐Davidson Resilience Scale 10‐item	Stress, anxiety and mindful attention awareness
Chiu‐Yueh et al. (2024)	Taiwan	Cross‐sectional	322	Single site hospital	Connor‐Davidson Resilience Scale 10‐item (Chinese translation)	Gratitude and thoughts of quitting
Cho et al. (2017)	South Korea	Cross‐sectional	179	Multisite hospital intensive care units	Connor‐Davidson Resilience Scale 25‐item (Korean translation)	Negative emotions and post‐traumatic stress
Choi and Kim (2022)	South Korea	Cross‐sectional	137	Multisite hospital	Connor‐Davidson Resilience Scale 25‐item (Korean translation)	Job stress, sleep quality and healthy behaviour patterns
Choi et al. (2022)	South Korea	Cross‐sectional	131	Multisite hospital emergency departments	Connor‐Davidson Resilience Scale 25‐item (Korean translation)	Perceived stress, experiences of violence, ability to cope with violence and responses to violence
Chua et al. (2024)	Singapore	Cross‐sectional	270	Multisite hospital	Brief Resilience Scale	Intent to stay, occupational self‐efficacy, working environment and insomnia severity
Chukwuorji et al. (2024)	Nigeria	Cross‐sectional	200	Single site hospital	Resilience Scale 14‐item	Spiritual involvement and beliefs and post‐traumatic growth
Chura et al. (2022)	Peru	Cross‐sectional	286	Multisite hospital	Connor‐Davidson Resilience Scale 10‐item (Spanish translation)	COVID‐19 pandemic‐related stress, health questionnaire and fear of COVID
Clark et al. (2021)	America	Cross‐sectional	175	Multisite hospital emergency departments	Connor‐Davidson Resilience Scale 25‐item	Moral distress and workplace engagement
Coetzee et al. (2024)	South Africa	Cross‐sectional	264	Multisite hospital	Connor‐Davidson Resilience Scale 25‐item	Satisfaction with life, sense of coherence, depression, perceived social support, fear of COVID‐19 and perceived vulnerability to disease
Connelly et al. (2023)	Canada	Cross‐sectional	434	Multisite aged care	Connor‐Davidson Resilience Scale 25‐item, Resilience at Work Scale and Resilience at Work Team Scale	Individual impacts of the COVID‐19 pandemic
Connelly et al. (2024)	Canada	Cross‐sectional	768	Multisite community	Connor‐Davidson Resilience Scale 10‐item and Resilience at Work Scale	Emotional intelligence
Converso et al. (2018)	Italy	Cross‐sectional	333	Multisite hospital	Connor‐Davidson Resilience Scale 10‐item	Job resources, self‐efficacy, hope, optimism and work ability
Converso et al. (2019)	Italy	Cross‐sectional	94	Multisite hospital	Connor‐Davidson Resilience Scale 10‐item (Italian translation)	Menopausal symptoms, burnout, job contentment, self‐efficacy, social support and optimism
Cooper et al. (2021)	Australia	Mixed methods (observational)	755	Single site hospital	Connor‐Davidson Resilience Scale 25‐item	Knowledge of organisational values for the organisation in which participants worked
Craigie et al. (2016)	Australia	Quasi‐experimental	21	Single site hospital	Connor‐Davidson Resilience Scale 10‐item	Depression, post‐traumatic stress, alcohol and substance misuse, compassion satisfaction, compassion fatigue, burnout, negative affect and passion for work
Çuhadar et al. (2023)	Turkey	Cross‐sectional	153	Single site hospital	Brief Resilience Scale (Turkish translation)	Stress
Dehvan et al. (2018)	Iran	Cross‐sectional	60	Single site hospital mental health	Connor‐Davidson Resilience Scale 25‐item	Mental health and well‐being
Delaney (2018)	Ireland	Mixed methods (observational)	13	Single site hospital	Connor‐Davidson Resilience Scale 25‐item	Self‐compassion, mindfulness, burnout, compassion satisfaction and compassion fatigue
Delgado et al. (2020)	Australia	Cross‐sectional	482	Multisite hospital and community mental health settings	Resilience at Work Scale	Emotional labour
Delgado et al. (2021)	Australia	Cross‐sectional	482	Multisite hospital and community mental health settings	Resilience at Work Scale	Psychological well‐being, depression, anxiety and stress
Ding et al. (2023)	China	Cross‐sectional	1774	Multisite hospital	Connor‐Davidson Resilience Scale 10‐item (Chinese translation)	Workplace violence, loneliness, sleep quality, perceived cognitive deficits, anxiety and social support
Diño et al. (2022)	Manila	Mixed methods (observational)	50	Multisite hospital	Connor‐Davidson Resilience Scale 10‐item	Motivation and intent to stay
Dolan, Strodl, and Hamernik (2012)	Australia	Mixed methods (observational)	16	Single site hospital haemodialysis	Resilience Scale 25‐Item	Burnout
Dordunoo et al. (2021)	South Korea	Cross‐sectional	199	Single site hospital	Connor‐Davidson Resilience Scale 10‐item	Work environment, burnout, compassion satisfaction and compassion fatigue
Du et al. (2024)	China	Cross‐sectional	924	Single site hospital	Brief Resilience Scale	Compliance with standard precautions, burnout and servant leadership
Durmuåÿ et al. (2024)	Turkey	Cross‐sectional	302	Single site hospital	Brief Resilience Scale (Turkish translation)	Workplace incivility, work stress, turnover intention and presenteeism
Ediz and Yanik (2024)	Turkey	Cross‐sectional	464	Multisite hospital and community	Brief Resilience Scale (Turkish translation)	Disaster preparedness, empathy
Fan et al. (2022)	China	Cross‐sectional	360	Single site hospital	Connor‐Davidson Resilience Scale 25‐item	Workplace violence and mental health
Foli et al. (2024)	America	Multi‐method (observational)	41	Nurses in recovery from substance abuse	Brief Resilience Scale	Post‐traumatic growth and perceived organisational support
Foster et al. (2018)	Australia	Quasi‐experimental	24	Multisite hospital mental health	Workplace Resilience Inventory	Depression, anxiety, stress, life satisfaction, psychological well‐being, work satisfaction and self‐efficacy
Foster et al. (2024a)	Australia	Cross‐sectional	144	Multisite mental health settings	Brief Resilience Scale	Psychological distress, well‐being, emotional intelligence, coping self‐efficacy, post‐traumatic growth, workplace belonging and turnover intention
Foster et al., (2024b)	Australia	Partially clustered randomised controlled trial	122	Multisite mental health settings	Brief Resilience Scale	Psychological distress, well‐being, emotional intelligence, coping self‐efficacy, post‐traumatic growth, workplace belonging and turnover intention
Foster et al. (2024c)	Australia	Cross‐sectional	87	Multisite mental health settings	Brief Resilience Scale	Well‐being, turnover intention, mental health stigma, perceived stress and work satisfaction
Fradelos et al. (2023)	Greece	Cross‐sectional	378	Multisite hospital general and mental health setting	Connor‐Davidson Resilience Scale 25‐item (Greek translation)	Health, anxiety and coping
Galanis et al. (2023)	Greece	Cross‐sectional	584	Multisite hospital	Connor‐Davidson Resilience Scale 10‐item (Greek translation)	Nil
Galura et al. (2022)	America	Cross‐sectional	149	Multisite hospital	Brief Resilience Scale	Job demands and resources, burnout, stress, job satisfaction and job performance
Gao et al. (2017)	China	Cross‐sectional	365	Single site hospital	Connor‐Davidson Resilience Scale 25‐item	Mental health and general well‐being
García et al. (2018)	Spain	Cross‐sectional	537	Multisite hospital	Connor‐Davidson Resilience Scale 10‐item (Spanish translation)	Burnout and mental health
Garcia‐Dia et al. (2018)	America	Cross‐sectional	150	Multisite hospital and community	Resilience Scale 14‐item	Nil
Georges et al. (2022)	Haiti	Cross‐sectional	179	Multisite hospital	Connor‐Davidson Resilience Scale 10‐item	Burnout, self‐efficacy and work environment
Ghahramani et al. (2023)	Iran	Cross‐sectional	280	Multisite hospital	Connor‐Davidson Resilience Scale 25‐item	Intention to quit
Gil‐Almagro et al. (2024)	Spain	Longitudinal	131	Multisite hospital critical care settings	Resilience Scale 14‐item (Spanish translation)	Depression, anxiety, stress, self‐efficacy and occupational hardiness
Giordano et al. (2024)	America	Cross‐sectional	177	Multisite hospital	Connor‐Davidson Resilience Scale 2‐item	Quality of life and well‐being
Gotlib et al. (2022)	Poland	Cross‐sectional	145	Multisite hospital	Brief Resilient Coping Scale (Polish translation)	Motivations towards COVID‐19 vaccinations, anxiety and self‐efficacy
Guo et al. (2017)	China	Cross‐sectional	1061	Multisite hospital	Connor‐Davidson Resilience Scale 25‐item	Self‐efficacy and coping style
Guo et al. (2018)	China	Cross‐sectional	1061	Multisite hospital	Connor‐Davidson Resilience Scale 25‐item	Burnout
Gündüz et al. (2024)	Turkey	Cross‐sectional	276	Single site hospital	Resilience Scale for Adults (Turkish translation)	Professional quality of life
Habibpour et al. (2023)	Iran	Cross‐sectional	158	Multisite hospital	Connor‐Davidson Resilience Scale 10‐item	Stress, compassion satisfaction, compassion fatigue and burnout
Hale et al. (2023)	America	Mixed methods (observational)	30	Multisite community	Brief Resilience Coping Scale	Qualitative open‐ended questions
Hamaideh et al. (2024)	Saudi Arabia	Cross‐sectional	140	Multisite hospital inpatient mental health	Connor‐Davidson Resilience Scale 25‐item	Perceived stress
Han et al. (2022)	China	Quasi‐experimental	226	Multisite hospital	Connor‐Davidson Resilience Scale 25‐item	Mental health and perceived stress
Han et al. (2023)	South Korea	Cross‐sectional	220	Multisite hospital	Connor‐Davidson Resilience Scale 25‐item (Korean translation)	Meaning in life and post‐traumatic growth
Harris et al. (2021)	America	Cross‐sectional	405	Multisite hospital	Connor‐Davidson Resilience Scale 25‐item	Job satisfaction and anticipated turnover
Harwood et al. (2024)	Canada	Cross‐sectional	42	Multisite hospital and community	Connor‐Davidson Resilience Scale 25‐item	Burnout and job satisfaction
Hasan and Alsulami (2024)	Saudi Arabia	Cross‐sectional	250	Single site hospital	Connor‐Davidson Resilience Scale 25‐item	Well‐being, depression, stress, anxiety and stress
Hassan et al. (2022)	Saudi Arabia	Cross‐sectional	219	Single site hospital mental health settings	Connor‐Davidson Resilience Scale 25‐item	Burnout and attitudes towards safety culture
He et al. (2021)	China	Cross‐sectional	709	Multisite hospital general and mental health	Connor‐Davidson Resilience Scale 10‐item	Grit, personality traits, self‐control and task performance
Hennen and Phillips (2023)	America	Cross‐sectional	35	Multisite hospital emergency departments	Brief Resilience Scale	Work performance, workplace aggression, mental health stigma and competency in caring for patients with mental illness
Honein‐AbouHaidar et al. (2023)	Lebanon	Cross‐sectional	1556	Multisite hospital and community	Resilience Scale 14‐item (Arabic translation)	Organisational and work factors
Hong et al. (2021)	South Korea	Cross‐sectional	842	Multisite hospital	Brief Resilience Scale	Stress, anxiety and mental health
Hoşgör and Yaman (2022)	Turkey	Cross‐sectional	284	Single site hospital	Brief Resilience Scale	Job performance
Hou et al. (2021)	China	Cross‐sectional	707	Multisite hospital	Connor‐Davidson Resilience Scale 25‐item	Social support and anxiety
Howie‐Esquivel et al. (2022)	America	Cross‐sectional	101	Multisite hospital and community	Brief Resilience Scale	Quality of life at work and burnout
Hsieh et al. (2016a)	Taiwan	Cross‐sectional	159	Multisite hospital emergency departments	Resilience Scale for Adults (Chinese translation)	Social support and depression
Hsieh et al. (2016b)	Taiwan	Cross‐sectional	187	Multisite hospital emergency departments	Resilience Scale for Adults (Chinese translation)	Social support, religious beliefs and personality traits
Hsieh et al. (2017)	Taiwan	Cross‐sectional	230	Multisite hospital emergency departments and mental health	Resilience Scale for Adults (Chinese translation)	Personality traits and social support
Hu et al. (2020)	China	Cross‐sectional	2014	Multisite hospital	Connor‐Davidson Resilience Scale 10‐item (Chinese translation)	Burnout, anxiety, depression, fear scale, presence of skin lesions, self‐efficacy and social support
Huang et al. (2021)	China	Cross‐sectional	953	Multisite hospital	Connor‐Davidson Resilience Scale 25‐item	Personality traits, coping styles, self‐efficacy and psychological distress
Huang et al. (2023)	China	Cross‐sectional	197	Multisite hospital emergency medical teams	Connor‐Davidson Resilience Scale 25‐item (Chinese translation)	Coping styles, anxiety and depression
Huang et al. (2024a)	China	Cross‐sectional	466	Online snowball sample of Emergency Nurses	Connor‐Davidson Resilience Scale 10‐item (Chinese translation)	Perceived organisational support and fear of future violence
Huang et al. (2024b)	China	Cross‐sectional	646	Single site hospital	Connor‐Davidson Resilience Scale 10‐item (Chinese translation)	Public health emergency competencies, perceived stress and coping
Huang et al. (2024c)	China	Cross‐sectional	1601	Multisite hospital	Resilience Scale 14‐item (Chinese translation)	Coping and empathy for pain
Hwang and Lee (2023)	South Korea	Cross‐sectional	131	Multisite hospital intensive care units	Connor‐Davidson Resilience Scale 25‐item (Korean translation)	Depression, job stress, sleep quality and burnout
Irwin et al. (2021)	America	Quasi‐experimental	42	Multisite hospital	Connor‐Davidson Resilience Scale 25‐item	Nil
Itzhaki et al. (2015)	Israel	Cross‐sectional	118	Single site hospital mental health	Connor‐Davidson Resilience Scale 10‐item (Hebrew translation)	Exposure to violence, perceived jobs stress, life satisfaction and post‐traumatic growth
Jamebozorgi et al. (2022)	Iran	Cross‐sectional	364	Multisite hospital	Connor‐Davidson Resilience Scale 25‐item	Burnout
Janzarik et al. (2022)	Germany	Randomised controlled trial	75	Single site hospital	Connor‐Davidson Resilience Scale 25‐item and Brief Resilience Scale	Mental health and micro stressors
Jeon and Kim (2023)	South Korea	Cross‐sectional	107	Single site hospital oncology setting	Connor‐Davidson Resilience Scale 25‐item (Korean translation)	Professional quality of life and caring behaviour
Jiménez et al. (2022)	Madrid	Cross‐sectional	375	Multisite hospital	Connor‐Davidson Resilience Scale 10‐item (Spanish translation)	Burnout and personality traits
Jo et al. (2023)	America, Japan and Korea	Cross‐sectional	662	Online snowball sample	Connor‐Davidson Resilience Scale 10‐item	Compassion fatigue, compassion satisfaction, burnout and secondary traumatic stress
Jose et al. (2020)	India	Cross‐sectional	120	Single site hospital emergency department	Connor‐Davidson Resilience Scale 25‐item	Burnout
Jose et al. (2022)	India	Cross‐sectional	137	Single site hospital intensive care units	Connor‐Davidson Resilience Scale 10‐item	Stress, anxiety, fear relating to the covid‐19 pandemic and insomnia
Joy et al. (2023)	Qatar	Cross‐sectional	300	Multisite hospital	Connor‐Davidson Resilience Scale 10‐item	Self‐esteem and self‐compassion
Jubin et al. (2022)	France	Cross‐sectional	9898	Members of the French National Order of Nurses	Connor‐Davidson Resilience Scale 10‐item (French translation)	Burnout, compassion satisfaction, compassion fatigue, stress, social support and coping skills
Jubin et al. (2023)	Switzerland	Longitudinal	1013 responses over three timepoints	Multisite hospital	Connor‐Davidson Resilience Scale 10‐item (French translation)	Perceived stress, post‐traumatic growth, perceived social support and psychosocial risk in the workplace
Jubin et al. (2024)	France, Switzerland, Portugal and Canada	Longitudinal	3310 responses over three timepoints	Multisite hospital	Connor‐Davidson Resilience Scale 10‐item (French, German and Portuguese translations)	Perceived stress, post‐traumatic growth, perceived social support, quality of life and well‐being
Jurado et al. (2022)	Spain	Cross‐sectional	1013	Online snowball sample	Resilience Scale 14‐item (Spanish translation)	Job strain and burnout
Kang et al. (2021)	South Korea	Cross‐sectional	435	Single site hospital	Connor‐Davidson Resilience Scale 25‐item (Korean translation)	Work‐place bullying, structural empowerment and nursing performance
Karabulak et al. (2021)	Turkey	Cross‐sectional	201	Multisite hospital and community	Brief Resilience Scale (Turkish translation)	Stress
Kartal et al. (2022)	Turkey	Cross‐sectional	156	Multisite hospital emergency departments	Brief Resilience Scale (Turkish translation)	Fear of death
Kayalar and Hicdurmaz (2024)	Turkey	Cross‐sectional	121	Multisite hospital oncology setting	Connor‐Davidson Resilience Scale 25‐item (Turkish translation)	Emotional regulation, self‐compassion and metacognitions
Ke et al. (2020)	China	Cross‐sectional	131	Multisite hospital operating rooms	Connor‐Davidson Resilience Scale 10‐item	Plasma monoamine neurotransmitter and serum cytokine levels
Kelly et al. (2021)	America	Cross‐sectional	1688	Multisite hospital	Connor‐Davidson Resilience Scale 10‐item	Burnout
Kılınç et al. (2021)	Turkey	Cross‐sectional	720	Multisite hospital	Connor‐Davidson Resilience Scale 25‐item (Turkish translation)	Social support
Kim et al. (2022)	South Korea	Cross‐sectional	206	Multisite hospital	Connor‐Davidson Resilience Scale 25‐item (Korean translation)	End‐of‐life care‐related stress and individuals “calling” for their work
Kim et al. (2024a)	America	Cross‐sectional	676	Multisite hospital and community	Resilience Scale 14‐item	Moral injury and well‐being
Kim et al. (2024b)	South Korea	Randomised Controlled Trial	112	Multisite hospital and community	Connor‐Davidson Resilience Scale 25‐item (Korean translation)	Functional health, social support, post‐traumatic stress, depression and anxiety
Kiratli and Duran (2024)	Turkey	Cross‐sectional	214	Healthcare setting not specified	Brief Resilience Scale (Turkish translation)	Compassion fatigue and loneliness at work
Kiziloglu et al. (2023)	Turkey	Cross‐sectional	325	Single site hospital	Connor‐Davidson Resilience Scale 25‐item (Turkish translation)	Personality type, fear of COVID‐19 and levels of stress
Kleier et al. (2022)	America	Cross‐sectional	189	Multisite hospital	Connor‐Davidson Resilience Scale 10‐item	Professional commitment
Kondo et al. (2024)	Japan	Cross‐sectional	229	Multisite hospital critical care settings	Connor‐Davidson Resilience Scale 25‐item (Japanese translation)	Attitudes towards care of the dying and sense of coherence
Kong et al. (2024)	China	Cross‐sectional	626	Multisite hospital	Connor‐Davidson Resilience Scale 10‐item (Chinese translation)	Job satisfaction, health‐related quality of life, self‐esteem, social support neuroticism, perceived prejudice and occupational stress
Koprowski et al. (2021)	America	Quasi‐experimental	419	Multisite hospital and community	Connor‐Davidson Resilience Scale 25‐item	Nil
Koutantelia et al. (2024)	Greece	Cross‐sectional	158	Multisite hospital paediatric settings	Connor‐Davidson Resilience Scale 25‐item (Greek translation)	Anxiety and depression
Kutluturkan et al. (2016)	Turkey	Cross‐sectional	140	Single site hospital oncology	Resilience Scale for Adults	Burnout
Labrague et al. (2020)	Philippines	Cross‐sectional	325	Multisite hospital	Brief Resilience Scale	COVID‐19‐related anxiety, social support and organisational support
Labrague et al. (2021a)	Philippines	Cross‐sectional	736	Multisite hospital and community	Brief Resilience Scale	COVID‐19‐related anxiety, social support and mental health
Labrague et al. (2021b)	Philippines	Cross‐sectional	270	Multisite hospital	Brief Resilience Scale	Compassion fatigue, job satisfaction, intention to leave and self‐appraised quality of care provided
Labrague et al. (2021c)	Philippines	Cross‐sectional	259	Multisite hospital	Brief Resilience Scale	COVID‐19‐associated discrimination, mental health and turnover intention
Labrague (2021)	Philippines	Cross‐sectional	255	Multisite hospital	Brief Resilience Scale	Pandemic fatigue, job contentment and sleep quality
Lan et al. (2023)	China	Randomised Controlled Trial	93	Multisite hospital emergency settings	Connor‐Davidson Resilience Scale 25‐item (Chinese translation)	Disaster nursing ability
Lang et al. (2022)	Australia	Cross‐sectional	239	Members of the Australian College of Perioperative Nurses	Connor‐Davidson Resilience Scale 10‐item	Workplace bullying and burnout
Lara‐Cabrera et al. (2021)	Spain	Cross‐sectional	332	Members of the Official College of Nursing of Tenerife	Resilience Scale 14‐item	Well‐being, stress, depressive symptoms and anxiety
Lee et al. (2022)	South Korea	Cross‐sectional	146	Multisite hospital operating rooms	Connor‐Davidson Resilience Scale 25‐item (Korean translation)	Job stress, burnout and communication competence
Lee et al. (2023a)	South Korea	Cross‐sectional	221	Single hospital	Connor‐Davidson Resilience Scale 25‐item (Korean translation)	Grit, calling and vocation and retention intention
Lee et al. (2023b)	Indonesia	Cross‐sectional	182	Multisite hospital and community	Connor‐Davidson Resilience Scale 25‐item (Indonesian translation)	Burnout and psychological empowerment
Lee et al. (2024a)	Taiwan	Cross‐sectional	387	Multisite hospital	Connor‐Davidson Resilience Scale 25‐item (Chinese translation)	Quality of work life, personal accomplishment and turnover intention
Lee et al. (2024b)	Taiwan	Cross‐sectional	319	Multisite hospital and community	Connor‐Davidson Resilience Scale 25‐item (Chinese translation)	Health‐related quality of life, general health and burnout
Lee and Lee (2022)	South Korea	Cross‐sectional	348	Multisite hospital	Connor‐Davidson Resilience Scale 25‐item	Job calling, relationship between leader and employee, workplace bullying, work environment, job satisfaction and intention to stay
Lei et al. (2024)	China	Cross‐sectional	522	Multisite hospital	Connor‐Davidson Resilience Scale 10‐item (Chinese translation)	Intimate partner violence, work environment and alienation at work
Leng et al. (2020)	China	Cross‐sectional	2981	Single site hospital	Connor‐Davidson Resilience Scale 25‐item (Chinese translation)	Stressors
Li et al. (2021a)	Taiwan	Cross‐sectional	132	Single site hospital emergency department	Connor‐Davidson Resilience Scale 10‐item	Workplace violence and intention to leave
Li et al. (2021b)	China	Cross‐sectional	143	Single site hospital intensive care unit	Connor‐Davidson Resilience Scale 25‐item	Burnout and social support
Li et al. (2022)	China	Cross‐sectional	318	Multisite hospital and aged care palliative care units	Connor‐Davidson Resilience Scale 25‐item (Chinese translation)	Self‐efficacy, social support, coping style, burnout, compassion satisfaction and compassion fatigue
Li et al. (2023)	China	Cross‐sectional	552	Single site hospital	Connor‐Davidson Resilience Scale 25‐item (Chinese translation)	Anxiety, depression and self‐efficacy
Li et al. (2024a)	China	Cross‐sectional	1613	Single site hospital	Connor‐Davidson Resilience Scale 25‐item (Chinese translation)	Workplace psychological violence and empathy
Li et al. (2024b)	China	Cross‐sectional	389	Multisite hospital	Connor‐Davidson Resilience Scale 10‐item (Chinese translation)	Social support and mindful self‐care
Li et al. (2024c)	China	Longitudinal	258	Multisite hospital	Connor‐Davidson Resilience Scale 10‐item (Chinese translation)	Emotional regulation and coping
Li et al. (2024d)	China	Cross‐sectional	1774	Multisite hospital	Connor‐Davidson Resilience Scale 10‐item (Chinese translation)	Perceived cognitive deficits, anxiety, depression, childhood trauma, social support, loneliness, sleep quality, suicidal ideation and non‐suicidal self‐injury
Li et al. (2024e)	China	Cross‐sectional	252	Multisite hospital	Connor‐Davidson Resilience Scale 25‐item (Chinese translation)	Transition shock, professional identity and self‐efficacy
Liao et al. (2019)	China	Cross‐sectional	597	Multisite hospital	Connor‐Davidson Resilience Scale 25‐item	Psychological distress, post‐traumatic stress disorder and post‐traumatic growth
Liao et al. (2024)	China	Cross‐sectional	1774	Multisite hospital	Connor‐Davidson Resilience Scale 10‐item (Chinese translation)	Loneliness, anxiety and burnout
Liat et al. (2024)	Israel	Cross‐sectional	200	Single site hospital	Connor‐Davidson Resilience Scale 10‐item (Hebrew translation)	Exposure to threatening events, adjustment disorder, psychological distress, positive affect and perceived social support
Lin et al. (2019)	China	Cross‐sectional	390	Multisite hospital	Resilience Scale for Adults (Chinese translation)	Work frustration and intent to stay
Lin et al. (2021)	America	Cross‐sectional	120	Multisite community long‐term care and rehabilitation settings	Connor‐Davidson Resilience Scale 25‐item	Sleep quality, burnout, compassion satisfaction and compassion fatigue
Lin et al. (2022)	China	Cross‐sectional	345	Multisite hospital gastroenterology	Connor‐Davidson Resilience Scale 25‐item (Chinese translation)	Burnout, emotional labour and work‐related characteristics
Liu et al. (2020)	China	Cross‐sectional	996	Multisite hospital	Connor‐Davidson Resilience Scale 25‐item (Chinese translation)	Fatigue, extrinsic effort and reward and perceived organisational support
Liu et al. (2021)	China	Cross‐sectional	200	Multisite hospital	Connor‐Davidson 10‐item Resilience Scale (Chinese translation)	Post‐traumatic growth, perceived professional benefit and intent to stay
Liu et al. (2023a)	China	Cross‐sectional	612	Multisite hospital	Connor‐Davidson Resilience Scale 10‐item	Depression and family functioning
Liu et al. (2023b)	China	Cross‐sectional	418	Multisite hospital	Connor‐Davidson Resilience Scale 25‐item (Chinese translation)	Rumination and post‐traumatic growth
Liu et al. (2023c)	China	Cross‐sectional	224	Snowball sampling of Registered Nurses undertaking postgraduate studies	Connor‐Davidson 10‐item Resilience Scale (Chinese translation)	Perceived stress, loneliness and interpersonal security
Liu et al. (2023d)	China	Cross‐sectional	200	Multisite hospital oncology settings	The Brief Resilience Scale (Chinese translation)	Perception of professional benefit, practice environment and transition shock
Liu et al. (2024a)	China	Cross‐sectional	828	Multisite hospital	Connor‐Davidson 10‐item Resilience Scale (Chinese translation)	Alexithymia and distress disclosure
Liu et al. (2024b)	China	Cross‐sectional	1032	Multisite hospital	Connor‐Davidson Resilience Scale 25‐item (Chinese translation)	Occupational benefit, sense of professional mission and work engagement
Liu et al. (2024c)	China	Cross‐sectional	719	Multisite hospital	Connor‐Davidson Resilience Scale 25‐item (Chinese translation)	Occupational benefit
LoGiudice and Bartos (2021)	America	Mixed methods (observational)	47	Multisite hospital	Brief Resilient Coping Scale	Qualitative data on experiences of working as a nurse during the COVID‐19 pandemic
Lu et al. (2023)	China	Cross‐sectional	13	Multisite hospital	Connor‐Davidson Resilience Scale 25‐item (Chinese translation)	Depression and burnout
Luo et al. (2022)	China	Cross‐sectional	458	Multisite hospital	Resilience Scale 25‐Item	Organisational support, leadership skills and burnout
Lyu et al. (2020)	China	Cross‐sectional	216	Multisite hospital	Connor‐Davidson Resilience Scale 25‐item (Chinese translation)	Work engagement and organisational identity
Magtibay et al. (2017)	America	Quasi‐experimental	50	Single site hospital	Connor‐Davidson Resilience Scale 2‐item	Happiness, stress, anxiety, mindfulness and burnout
Majrabi et al. (2021)	Saudi Arabia	Cross‐sectional	219	Single site hospital mental health	Connor‐Davidson Resilience Scale 25‐item	Burnout and attitude towards safety culture
Mallon et al. (2023)	Northern Ireland	Cross‐sectional	56	Multisite community older adult setting	Connor‐Davidson Resilience Scale 25‐item	Nil
Manzano García and Ayala Calvo (2012)	Spain	Cross‐sectional	200	Multisite hospital	Connor‐Davidson Resilience Scale 25‐item	Burnout and emotional annoyance
Mao et al. (2021)	China	Randomised controlled trial	103	Single site hospital	Connor‐Davidson Resilience Scale 25‐item (Chinese translation)	Emotional intelligence and stress
Mao et al. (2023)	China	Cross‐sectional	784	Multisite hospital	Connor‐Davidson Resilience Scale 25‐item (Chinese translation)	Perceived social support, self‐efficacy and post‐traumatic stress
Mao et al. (2024)	China	Cross‐sectional	784	Multisite hospital	Connor‐Davidson Resilience Scale 25‐item (Chinese translation)	Anxiety, depression, insomnia, post‐traumatic stress, perceived social support, self‐efficacy and burnout
Martins et al. (2022)	Portugal	Cross‐sectional	379	Online convenience sample	Resilience Scale 25‐Item (Portuguese translation)	Depression, anxiety, stress and burnout
McCoy, Sauer, and Sha (2023)	America	Cross‐sectional	345	Nurses registered with the State Board of Nursing	Resilience Scale 25‐item and Resilience Scale 14‐item	Bullying, stress and quality of life
Mealer et al. (2012)	America	Cross‐sectional	744	Members of the American Association of Critical Care Nurses intensive care units	Connor‐Davidson Resilience Scale 25‐item	Post‐traumatic stress, anxiety, depression and burnout
Mealer et al. (2014)	America	Randomised controlled trial	29	Single site hospital intensive care units	Connor‐Davidson Resilience Scale 25‐item	Post‐traumatic stress, anxiety, depression and burnout
Mealer et al. (2016)	America	Cross‐sectional	744	Members of the American Association of Critical Care Nurses intensive care units	Connor‐Davidson Resilience Scale 25‐item	Post‐traumatic stress
Mehdizadeh et al. (2024)	Iran	Cross‐sectional	224	Multisite hospital	Connor‐Davidson Resilience Scale 25‐item (Persian translation)	Perceived social support and perceived organisational support
Mei et al. (2022)	China	Cross‐sectional	470	Newly graduated nurses healthcare setting not specified	Connor‐Davidson Resilience Scale 10‐item (Chinese translation)	Mental health
Meng et al. (2023)	China	Cross‐sectional	356	Single site hospital	Connor‐Davidson Resilience Scale 25‐item (Chinese translation)	Career identity, work‐related quality of life and work engagement
Mensah et al. (2024)	Ghana	Cross‐sectional	343	Multisite hospital	Brief Resilience Scale	Sexual harassment, psychological well‐being and conflict resolution
Mersi et al. (2022)	Iran	Cross‐sectional	91	Single site hospital	Connor‐Davidson Resilience Scale 25‐item (Persian translation)	Mental and spiritual distress
Meyer and Shatto (2018)	America	Longitudinal (observational)	17	Direct Entry Accelerated Master's in Nursing graduates	Resilience Scale 25‐Item	Education satisfaction and graduate nurse experience
Mills et al. (2017)	Australia	Cross‐sectional	161	Multisite hospital and community	Connor‐Davidson Resilience Scale 10‐item	Nurse self‐concept, practice environment and nurse retention
Min et al. (2023)	South Korean	Cross‐sectional	235	Single site hospital	Connor‐Davidson Resilience Scale 25‐item (Korean translation)	Burnout, depression, anxiety and perceived stress
Mintz‐Binder et al. (2021)	America	Quasi‐experimental	77	Multisite hospital	Connor‐Davidson Resilience Scale 10‐item	Stress levels and sources of stress
Mohammad et al. (2023)	Egypt	Cross‐sectional	285	Single site hospital	Connor‐Davidson Resilience Scale 10‐item (Arabic translation)	Authentic leadership and self‐efficacy
Moisoglou et al. (2024)	Greece	Cross‐sectional	963	Multisite hospital and community	The Brief Resilience Scale (Greek translation)	Perceived social support and COVID‐19 burnout
Montgomery et al. (2022)	America	Cross‐sectional	56	Multisite hospital	Connor‐Davidson Resilience Scale 25‐item	Work environment, burnout and intent to leave
Montgomery and Patrician (2024)	America	Mixed methods (observational)	57	Multisite hospital	Connor‐Davidson Resilience Scale 25‐item	COVID stress
Mousavi et al. (2023)	Iran	Cross‐sectional	300	Multisite hospital	Connor‐Davidson 25‐Item Resilience Scale (Persian translation)	Fear of COVID‐19, job‐related stress, turnover intention, mental health, mental workload and work–family conflict
Muir et al. (2022)	America	Quasi‐experimental	97	Single site hospital	Connor‐Davidson Resilience Scale 10‐item	Questionnaire regarding intervention quality
Nantsupawat et al. (2024)	Thailand	Cross‐sectional	394	Multisite hospital	Connor‐Davidson Resilience Scale 10‐item (Thai translation)	Intention to leave, burnout and work engagement
Nassar et al. (2024)	Jordan	Cross‐sectional	161	Single site hospital	Resilience at Work Scale (Arabic translation)	Compassionate care
Nijland et al. (2021)	Netherlands	Quasi‐experimental	86	Single site hospital intensive units	Connor‐Davidson Resilience Scale 10‐item	Stress
Nikmanesh and Khosravi (2020)	Zahedan	Quasi‐experimental	38	Singe site hospital	Connor‐Davidson Resilience Scale 25‐item	Psychological well‐being
Norful et al. (2024)	America, Saudi Arabia, Philippines	Cross‐sectional	2864	International online snowball sample	The Brief Resilience Scale	Burnout, depression, anxiety, intention to leave and job satisfaction
Norouzinia et al. (2022)	Iran	Mixed methods (observational)	254	Single site hospital emergency department	Emergency Nurse’ professional resilience tool	Nil
Nourollahi‐Darabad et al. (2021)	Iran	Cross‐sectional	387	Multisite hospital	Connor‐Davidson Resilience Scale 25‐item	Emotional demand, leadership quality, work–family conflict, burnout, stress and job satisfaction
Öksüz et al. (2019)	Turkey	Cross‐sectional	242	Multisite hospital	Resilience Scale for Adults (Turkish translation)	Social support and job satisfaction
Ou et al. (2021)	China	Cross‐sectional	92	Single site hospital	Connor‐Davidson Resilience Scale 25‐item (Chinese translation)	Psychological health
Owens et al. (2023)	America	Quasi‐experimental	77	Multisite hospital mental health setting	Resilience Scale 14‐item	Stress and coping
Ozbek et al. (2022)	Turkey	Cross‐sectional	202	Single site hospital	Brief Resilience Scale (Turkish translation)	Traumatic stress symptoms
Pachi et al. (2024)	Greece	Cross‐sectional	441	Members of professional nursing associations	Brief Resilience Scale (Greek translation)	Insomnia and anger reactions
Pachi et al. (2024)	Greece	Cross‐sectional	433	Members of professional nursing associations	Brief Resilience Scale (Greek translation)	Nightmare distress and insomnia
Pallesen et al. (2022)	Denmark	Cross‐sectional	59	Single site hospital	Brief Resilience Scale	Burnout
Parizad et al. (2022)	Iran	Cross‐sectional	233	Single site hospital	Connor‐Davidson Resilience Scale 25‐item	Nil
Partridge et al. (2024)	America	Cross‐sectional	618	Multisite site hospital and community	Connor‐Davidson Resilience Scale 25‐item	Hope and work effectiveness
Park and Jung (2021)	South Korea	Cross‐sectional	200	Multisite site hospital	Connor‐Davidson Resilience Scale 25‐item (Korean translation)	Nursing professionalism and job stress
Park and Park (2021)	South Korea	Cross‐sectional	340	Single site hospital oncology	Connor‐Davidson Resilience Scale 25‐item (Korean translation)	Work‐related characteristics, attitude towards dignified death, compassion competence and occupational stress
Pehlivan and Güner (2020)	Turkey	Randomised controlled trial	125	Multisite hospital oncology and haematology	Resilience Scale for Adults (Turkish translation)	Stress, burnout, compassion satisfaction and compassion fatigue
Peñacoba et al. (2021)	Spain	Cross‐sectional	308	Multisite hospital intensive care units	Resilience Scale 14‐item	Stress and self‐efficacy
Peng et al. (2022)	China	Cross‐sectional	493	Multisite hospital	Connor‐Davidson Resilience Scale 10‐item (Chinese translation)	Workplace bullying and compassion fatigue
Phillips et al. (2022)	Canada	Cross‐sectional	4425	Multisite hospital and community	Connor‐Davidson Resilience Scale 10‐item	Health‐related quality of life and perceptions of the work environment
Pintus et al. (2024)	Italy	Cross‐sectional	29	Multisite hospital	Resilience Scale 14‐item (Italian translation)	Burnout, work engagement, depression, anxiety, stress and mindful awareness
Prodromou et al. (2023)	Cyprus	Cross‐sectional	470	Nurses registered with the Cyprus Nursing and Midwifery Association	Connor‐Davidson Resilience Scale 25‐item (Greek translation)	Burnout
Pu et al. (2024)	China	Cross‐sectional	1402	Multisite hospital	Connor‐Davidson Resilience Scale 25‐item (Chinese translation)	Perceived organisational support and intention to stay
Qi et al. (2022)	China	Cross‐sectional	839	Multisite hospital	Connor‐Davidson Resilience Scale 25‐item (Chinese translation)	Work–family conflict and anxiety
Qin et al. (2023)	China	Cross‐sectional	709	Online snowball sample	Connor‐Davidson Resilience Scale 10‐item (Chinese translation)	Emotional intelligence, self‐efficacy and life satisfaction
Rahmat et al. (2023)	Indonesia	Cross‐sectional	101	Single site hospital mental health	Connor‐Davidson Resilience Scale 25‐item	Stress and anxiety
Rashidi et al. (2023)	Turkey	Cross‐sectional	158	Multisite hospital intensive care and palliative care settings	Resilience Scale for Adults (Turkish translation)	Thanatophobia
Ren et al. (2018)	China	Cross‐sectional	1356	Multisite hospital	Connor‐Davidson Resilience Scale 25‐item (Chinese translation)	Self‐efficacy, coping style and job stress
Reyes et al. (2024)	America	Quasi‐experimental	60	Online snowball sample	Connor‐Davidson Resilience Scale 25‐item	Post‐traumatic stress disorder, experiential avoidance, rumination, mindfulness, intervention satisfaction and system usability
Rhéaume and Breau (2022)	Canada	Mixed methods (observational)	236	Multisite hospital intensive care units	Brief Resilience Scale	Burnout, experience during Covid‐19, moral distress and intent to leave
Rhoden et al. (2021)	Brazil	Longitudinal (observational)	53	Single site hospital	Resilience Scale 14‐item (Portuguese translation)	Occupational stress and osteomuscular pain
Rhoden et al. (2022)	Brazil	Longitudinal (observational)	53	Single site hospital	Resilience Scale 14‐item (Portuguese translation)	Stress
Rivas et al. (2021)	Spain	Cross‐sectional	101	Single site hospital	Connor‐Davidson Resilience Scale 10‐item (Spanish translation)	Burnout
Roberts et al. (2021)	United Kingdom	Cross‐sectional	255	Online convenience sample	Resilience Scale 14‐item	Anxiety and depression
Roberts et al. (2022)	United Kingdom	Cross‐sectional	161	Online convenience sample	Resilience Scale 14‐item	Anxiety and depression
Rogers et al. (2022)	Global	Cross‐sectional	928	Online snowball sample	Connor‐Davidson Resilience Scale 10‐item	Mental well‐being and spiritual well‐being
Rosa‐Besa et al. (2021)	America	Cross‐sectional	25	Single site hospital	Connor‐Davidson Resilience Scale 25‐item	Work stressors
Ruhabad et al. (2022)	Iran	Cross‐sectional	299	Multisite hospital	Connor‐Davidson Resilience Scale 25‐item	Coping strategies
Rushton et al. (2015)	America	Cross‐sectional	114	Multisite hospital	Connor‐Davidson Resilience Scale 25‐item	Burnout, moral distress, stress, perceived meaningfulness of work and hope
Rushton et al. (2021)	America	Quasi‐experimental	415	Multisite hospital	Brief Resilience Scale	Ethical confidence, moral sensitivity/moral decision‐making, moral competence, moral distress, empathy, work engagement, burnout, turnover intention, psychiatric symptoms and mindful attention and awareness
Rushton et al. (2023)	America	Quasi‐experimental	245	Multisite hospital	Brief Resilience Scale	Ethical confidence, moral competence, work engagement, mindful attention awareness, psychiatric symptoms, turnover intention, burnout, emotional empathy, moral sensitivity and moral distress
Sacgaca et al. (2023)	Saudi Arabia	Cross‐sectional	763	Multisite hospital	Connor‐Davidson Resilience Scale 10‐item	Mental well‐being, stress and coping strategies
Saez‐Ruiz et al. (2024)	Spain	Cross‐sectional	201	Multisite hospital and community	Connor‐Davidson Resilience Scale 10‐item	Nurse–patient therapeutic communication
Salam et al. (2023)	Lebanon	Cross‐sectional	240	Multisite hospital	Resilience Scale 25‐Item	Perceptions of transformational leadership
Sampaio et al. (2022)	Portugal	Cross‐sectional	247	Online convenience sample	Connor‐Davidson Resilience Scale 10‐item (Portuguese translation)	Quality of life, workplace well‐being, stress, social support, job satisfaction and personal identification
Sani et al. (2020)	Iran	Cross‐sectional	118	Multisite hospital emergency departments	Connor‐Davidson Resilience Scale 25‐item	Workplace violence
Santos et al. (2024)	Brazil	Randomised crossover trial	32	Single site hospital	Connor‐Davidson Resilience Scale 25‐item	Emotional intelligence, mindfulness
Sauer and McCoy (2017)	America	Cross‐sectional	345	Nurses registered with the State Board of Nursing	Resilience Scale 25‐Item	Workplace bullying, mental health, physical health and stress
Sawalma et al. (2024)	Palestine	Cross‐sectional	273	Multisite hospital critical care settings	Connor‐Davidson Resilience Scale 25‐item	Depression, anxiety, stress, work engagement
Sawyer et al. (2022)	America	Mixed methods (interventional)	16	Multisite hospital	Brief Resilience Scale	Self‐compassion, psychological empowerment, self‐reflection, self‐insight post‐traumatic growth, burnout, job satisfaction and stress
Sawyer et al. (2023)	America	Randomised controlled trial	30	Multisite hospital	Brief Resilience Scale	Post‐traumatic growth, burnout, compassion satisfaction, compassion fatigue, self‐reflection, self‐insight, self‐compassion, psychological empowerment, self‐efficacy, stress and job satisfaction
Selvi and Yilmaz (2023)	Turkey	Cross‐sectional	189	Multisite hospital	Resilience Scale for Adults (Turkish translation)	Authenticity
Senturk et al. (2024)	Turkey	Cross‐sectional	76	Single site hospital	Resilience Scale for Adults (Turkish translation)	Sleepiness, sleep disturbance and depression
Sexton et al. (2024)	America	Cross‐sectional	79	Multisite hospital paediatric emergency settings	Connor‐Davidson Resilience Scale 25‐item	Moral distress
Shahrbabaki et al. (2023)	Iran	Cross‐sectional	300	Single site hospital	Connor‐Davidson Resilience Scale 25‐item	Job satisfaction
Shen et al. (2023)	China	Cross‐sectional	178	Single site hospital	Connor‐Davidson Resilience Scale 25‐item (Chinese translation)	Life satisfaction and depression
Shen et al. (2024)	China	Cross‐sectional	298	Single site hospital	Connor‐Davidson Resilience Scale 25‐item (Chinese translation)	Thriving at work and work performance
Shi et al. (2018)	China	Cross‐sectional	396	Single site hospital	Brief Resilient Coping Scale	Workplace incivility, anxiety and burn‐out
Shi et al. (2024)	China	Cross‐sectional	361	Multisite military hospitals	Workplace Resilience Scale	Infectious disease emergency response capacity
Shin and Choi (2023)	South Korea	Cross‐sectional	120	Single site hospital intensive care unit	Connor‐Davidson Resilience Scale 25‐item (Korean translation)	Burnout, compassion satisfaction, compassion fatigue and post‐traumatic stress
Siami et al. (2023)	Iran	Cross‐sectional	623	Multisite hospital	Brief Resilience Scale	Safety, leadership and personal hope
Sikioti et al. (2023)	Greece	Cross‐sectional	153	Multisite hospital	Brief Resilience Scale	Burnout, stress, consequences experienced by healthcare professionals in COVID‐19 referral hospitals
Soltanian et al. (2023)	Iran	Cross‐sectional	78	Multisite hospital neonatal intensive care units	Connor‐Davidson Resilience Scale 25‐item	Self‐awareness, positive beliefs and belonging
Son and Ham (2020)	South Korea	Cross‐sectional	438	Multisite hospital	Connor‐Davidson Resilience Scale 25‐item (Korean translation)	Insomnia, job satisfaction and work–life balance
Stanton et al. (2015)	America	Quasi‐experimental	7	Single site hospital	Resilience Scale 25‐Item	Compassion fatigue, stress and empathy
Suazo et al. (2024)	Spain	Cross‐sectional	1013	Multisite hospital and community	Resilience Scale 14‐item (Spanish translation)	Burnout, general health
Sukut et al. (2022)	Turkey	Cross‐sectional	100	Single site hospital mental health	Connor‐Davidson Resilience Scale 25‐item (Turkish translation)	Burnout, compassion satisfaction and compassion fatigue
Sullivan et al. (2019)	America	Mixed methods (observational)	59	Single site hospital paediatric oncology	Connor‐Davidson Resilience Scale 2‐item	Burnout, compassion satisfaction, compassion fatigue and coping skills
Sun et al. (2022)	China	Cross‐sectional	340	Multisite hospital intensive care units	Brief Resilient Coping Scale	Job calling, thriving at work and ethical leadership
Ta'an et al. (2024)	Jordan	Cross‐sectional	100	Multisite hospital	Brief Resilience Scale (Arabic translation)	Depression, anxiety, stress and nursing performance
Tabakakis et al. (2019)	New Zealand	Cross‐sectional	480	Registered Nurses randomly selected from the New Zealand Nurses Organisation	Connor‐Davidson Resilience Scale 10‐item	Practice environment and workplace bullying
Taghighi et al. (2019)	Australia	Cross‐sectional	1495	Registered and Enrolled Nurses who were members of the Queensland Nurses and Midwives Union	Connor‐Davidson Resilience Scale 25‐item	Depression, anxiety, compassion satisfaction, compassion fatigue and intention to leave
Talebian et al. (2022)	Iran	Cross‐sectional	144	Multisite hospital intensive care units	Connor‐Davidson Resilience Scale 25‐item	Moral distress
Tang et al. (2022)	China	Cross‐sectional	709	Multisite hospital	Connor‐Davidson Resilience Scale 10‐item (Chinese translation)	Personality traits, grit and life meaning
Tang et al. (2024)	China	Cross‐sectional	118	Single site hospital emergency setting	Connor‐Davidson Resilience Scale 10‐item (Chinese translation)	Professional quality of life, family care and organisational support
Tseng et al. (2018)	Taiwan	Cross‐sectional	83	Single site hospital burns unit	Connor‐Davidson Resilience Scale 25‐item	Stress, secondary traumatic stress, burnout and compassion fatigue
Tsouvelaz et al. (2022)	Greece	Cross‐sectional	222	Online convenience and snowball sample hospital and community	Brief Resilience Scale	Secondary traumatic stress and coping
Turan and Canbulat (2023)	Turkey	Quasi‐experimental	122	Single site hospital	Resilience Scale for Adults	Depression
Turan (2020)	Turkey	Quasi‐experimental	32	Single site hospital intensive care unit	Resilience Scale for Adults (Turkish translation)	Positive and negative affect
Turunç et al. (2024)	Turkey	Cross‐sectional	228	Multisite hospital	Connor‐Davidson Resilience Scale 10‐item (Turkish translation)	Burnout
Uzar‐Ozcetin et al. (2019)	Turkey	Cross‐sectional	61	Multisite hospital oncology	Connor‐Davidson Resilience Scale 25‐item (Turkish translation)	Burnout and psychological well‐being
Uzar‐Ozcetin and Budak (2024)	Turkey	Cross‐sectional	118	Multisite hospital oncology settings	Connor‐Davidson Resilience Scale 10‐item (Turkish translation)	Work‐related rumination, attitudes towards death
Villa et al. (2021)	Switzerland	Cross‐sectional	548	Multisite hospital	Brief Resilience Scale	Ethical conflict during COVID‐19 pandemic and psychological distress
Vogt et al. (2024)	United Kingdom	Mixed methods (interventional)	84	Multisite hospital and community	Brief Resilience Scale	Coping with adverse events, knowledge of resilience, burnout, depression and intention to leave
Walpita et al. (2020)	Sri Lanka	Cross‐sectional	230	Multisite hospital	Resilience at Work Scale (Sinhala translation)	Nursing performance
Walpita et al. (2022)	Sri Lanka	Cross‐sectional	855	Multisite hospital	Resilience at Work Scale (Sinhala translation)	Nil
Wang and Dela Rosa (2022)	China	Cross‐sectional	152	Single site hospital	Connor‐Davidson Resilience Scale 25‐item (Chinese translation)	Coping style
Wang et al. (2018)	China	Cross‐sectional	747	Multisite hospital	Connor‐Davidson Resilience Scale 25‐item (Chinese translation)	General self‐efficacy and social support
Wang et al. (2022)	China	Cross‐sectional	838	Multisite hospital	Connor‐Davidson Resilience Scale 10‐item	Nil
Wang et al. (2023)	China	Cross‐sectional	355	Multisite hospital intensive care units	Connor‐Davidson Resilience Scale 25‐item (Chinese translation)	Psychological distress, occupational coping and self‐efficacy
Wang et al. (2024a)	China	Cross‐sectional	212	Multisite hospital	Connor‐Davidson Resilience Scale 25‐item (Chinese translation)	Meaning of life and death attitudes
Wang et al. (2024b)	China	Randomised control trial	99	Single site hospital mental health setting	Connor‐Davidson Resilience Scale 25‐item (Chinese translation)	Mindfulness, burnout
Wang et al. (2024c)	China	Cross‐sectional	1202	Multisite hospital mental health setting	Connor‐Davidson Resilience Scale 25‐item (Chinese translation)	Post‐traumatic growth, empathy, coping with patient aggression and post‐traumatic stress
Wang et al. (2024d)	Sierra Leone	Cross‐sectional	360	Multisite hospital	Connor‐Davidson Resilience Scale 25‐item	Caring behaviours, job satisfaction and anxiety
Waterworth et al. (2021)	New Zealand	Cross‐sectional	197	Single site hospital paediatrics	Connor‐Davidson Resilience Scale 25‐item	Burnout
Wei et al. (2014)	China	Cross‐sectional	244	Multisite hospital	Personal Resilience Tool	Physiological needs satisfaction, organisational socialisation, conscientiousness, worry, Chinese values, future orientation and career success
Welden et al. (2023)	America	Cross‐sectional	859	Multisite hospital	Connor‐Davidson Resilience Scale 25‐item	Physical, mental and social health
Welden et al. (2021)	America	Cross‐sectional	859	Multisite hospital	Connor‐Davidson Resilience Scale 25‐item	General physical and mental health
Williams et al. (2016)	Canada	Cross‐sectional	130	Aged care	Resilience Scale for Adults	Absenteeism, personhood in dementia, organisational empowerment and quality of care
Wu et al. (2024)	China	Cross‐sectional	246	Single site hospital	Connor‐Davidson Resilience Scale 25‐item (Chinese translation)	Social support and anxiety
Xia et al. (2022)	China	Cross‐sectional	1582	Single site hospital	Connor‐Davidson Resilience Scale 10‐item (Chinese translation)	Work pressure, burnout, compassion satisfaction and compassion fatigue
Xiaoyi et al. (2021)	China	Cross‐sectional	496	Multisite hospital	Connor‐Davidson Resilience Scale 10‐item	Work engagement, compassion fatigue and turnover intention
Xu et al. (2024)	China	Cross‐sectional	471	Multisite hospital	Connor‐Davidson Resilience Scale 25‐item (Chinese translation)	Work fatigue
Xue et al. (2022)	China	Cross‐sectional	2266	Multisite hospital	Connor‐Davidson Resilience Scale 25‐item (Chinese translation)	Career success and craftsmanship
Yan et al. (2022a)	China	Cross‐sectional	1536	Multisite hospital infectious disease departments	Connor‐Davidson Resilience Scale 25‐item (Chinese translation)	Occupational stress and quality of life
Yan et al. (2022b)	China	Cross‐sectional	845	Multisite hospital infectious disease departments	Connor‐Davidson Resilience Scale 25‐item (Chinese translation)	Social support and quality of life
Yan et al. (2023)	China	Cross‐sectional	1224	Multisite hospital	Connor‐Davidson Resilience Scale 25‐item (Chinese translation)	Social support and quality of life
Yan et al. (2024)	China	Cross‐sectional	941	Multisite hospital	Connor‐Davidson Resilience Scale 25‐item (Chinese translation)	Competency, mindfulness
Yang et al. (2018)	China	Cross‐sectional	536	Multisite hospital transplant services	Connor‐Davidson Resilience Scale 25‐item	Burnout
Yang et al. (2022)	China	Cross‐sectional	2101	Multisite hospital	Connor‐Davidson Resilience Scale 10‐item (Chinese translation)	Presence of skin lesions, anxiety, depression and fear
Yang et al. (2023a)	China	Cross‐sectional	196	Nurses completing a Master of Nursing Specialist degree	Connor‐Davidson 10‐item Resilience Scale (Chinese translation)	Self‐regulated learning and mindful agency
Yang et al. (2023b)	China	Cross‐sectional	330	Multisite hospital	Connor‐Davidson Resilience Scale 25‐item (Chinese translation)	Moral courage, ethical climate and moral distress
Yao et al. (2023)	China	Cross‐sectional	1512	Single site hospital	Connor‐Davidson Resilience Scale 25‐item (Chinese translation)	Professional identity, organisational support and post‐traumatic growth
Yazdanirad et al. (2024)	Iran	Cross‐sectional	300	Multisite hospital	Connor‐Davidson Resilience Scale 25‐item (Persian translation)	Fear of COVID‐19, job stress, turnover intention, general mental health, work–family conflict and mental workload
Yeh et al. (2024)	Taiwan	Cross‐sectional	215	Single site hospital	Resilience Scale 14‐item	COVID‐19‐related stress, coping strategies and compassion fatigue
Yi et al. (2023)	China	Cross‐sectional	982	Multisite hospital	Connor‐Davidson Resilience Scale 10‐item (Chinese translation)	Professional identity and self‐efficacy
Yildirim et al. (2024)	Turkey	Cross‐sectional	230	Single site hospital	Brief Resilience Scale (Turkish translation)	Burnout
Ying et al. (2021)	Malaysia	Cross‐sectional	229	Single site hospital adult, paediatric and neonatal intensive care units	Connor‐Davidson Resilience Scale 25‐item	Perceptions of the work environment and future job plan
Yousefzadeh et al. (2024)	Iran	Randomised control trial	27	Single site hospital mental health setting	Connor‐Davidson Resilience Scale 10‐item (Persian translation)	Quality of work life and communication skills
Yu et al. (2018)	South Korea	Cross‐sectional	371	Multisite hospital	Connor‐Davidson Resilience Scale 10‐item	Work environment satisfaction, emotional labour and burnout
Yu et al. (2020)	New Zealand	Cross‐sectional	93	Multisite hospital intensive care units	Connor‐Davidson Resilience Scale 25‐item	Physical activity
Yu et al. (2021)	Taiwan	Cross‐sectional	272	Multisite hospital	Connor‐Davidson Resilience Scale 25‐item	Social support, professional commitment and intention to stay in nursing
Yu et al. (2022)	China	Cross‐sectional	358	Single site hospital	Connor‐Davidson Resilience Scale 10‐item	Mindfulness, anxiety, burnout, emotional regulation, stress perception, well‐being and loneliness
Yu‐Chin et al. (2023)	America	Cross‐sectional	110	Members of the American Association of Critical Care Nurses	Brief Resilience Scale	Trauma, anxiety, post‐traumatic stress, depression, organisation support, substance abuse, personality traits and cognitive control
Yun et al. (2022)	China	Time‐lagged (observational)	845	Multisite hospital	Resilience at Work Scale	High‐performance work systems, burnout and thriving at work
Yusefi et al. (2021)	Iran	Cross‐sectional	312	Single site hospital	Connor‐Davidson Resilience Scale 25‐item	Hypochondriasis
Zahednezhad et al. (2021)	Iran	Cross‐sectional	202	Multisite hospital	Connor‐Davidson Resilience Scale 10‐item (Persian translation)	Burnout, positive and negative affect and quality of working life
Zakeri et al. (2021)	Iran	Cross‐sectional	185	Single site hospital	Connor‐Davidson Resilience Scale 25‐item	Mental health and anxiety
Zeng et al. (2024)	China	Cross‐sectional	378	Multisite hospital	Resilience Scale 14‐item (Chinese translation)	Social support and post‐traumatic growth
Zhan et al. (2024)	China	Cross‐sectional	1874	Multisite hospital	Connor‐Davidson Resilience Scale 25‐item (Chinese translation)	Negative emotions and insomnia
Zhang et al. (2021a)	China	Cross‐sectional	992	Multisite hospital	Connor‐Davidson Resilience Scale 25‐item (Chinese translation)	Burnout, compassion satisfaction, compassion fatigue and self‐efficacy
Zhang et al. (2021b)	China	Cross‐sectional	180	Multisite hospital	Connor‐Davidson Resilience Scale 25‐item	Burnout and positive and negative affect
Zhang et al. (2022a)	China	Cross‐sectional	319	Multisite hospital palliative care	Connor‐Davidson Resilience Scale 25‐item (Chinese translation)	Burnout, social support, self‐efficacy and coping style
Zhang et al. (2022b)	China	Cross‐sectional	143	Single site hospital	Connor‐Davidson Resilience Scale 10‐item	Depression, anxiety and stress
Zhang et al. (2023)	China	Cross‐sectional	319	Multisite hospital	Connor‐Davidson Resilience Scale 25‐item	Burnout and social support
Zhang et al. (2024a)	China	Cross‐sectional	879	Multisite hospital infectious disease settings	Connor‐Davidson Resilience Scale 25‐item (Chinese translation)	Risk perception, social support and quality of working life
Zhang et al. (2024b)	China	Cross‐sectional	355	Multisite hospital intensive care settings	Connor‐Davidson Resilience Scale 25‐item (Chinese translation)	Career success and professional mission
Zhang et al. (2024c)	China	Cross‐sectional	694	Single site hospital	Connor‐Davidson Resilience Scale 2‐item	Insomnia, PTSD, fear of COVID‐19 and COVID‐19 burden
Zhang et al. (2024d)	China	Cross‐sectional	11,827	Multisite hospital	Connor‐Davidson Resilience Scale 25‐item (Chinese translation)	Burnout and general well‐being
Zhang et al. (2024e)	China	Cross‐sectional	271	Single site hospital	Connor‐Davidson Resilience Scale 25‐item (Chinese translation)	Belonging and presenteeism
Zhao and Hu (2023)	China	Cross‐sectional	380	Multisite hospital emergency departments	Connor‐Davidson Resilience Scale 10‐item	Psychological distress
Zhao et al. (2020)	China	Cross‐sectional	322	Multisite hospital	Connor‐Davidson Resilience Scale 25‐item (Chinese translation)	Turnover intention, job satisfaction and social support
Zhao et al. (2022)	China	Cross‐sectional	7231	Multisite hospital	Resilience Scale 14‐item (Chinese translation)	Nil
Zheng et al. (2023)	China	Cross‐sectional	2331	Multisite hospital	Connor‐Davidson Resilience Scale 25‐item (Chinese translation)	Organisational trust and trust in patients
Zhou et al. (2024)	China	Cross‐sectional	1279	Multisite hospital	Connor‐Davidson Resilience Scale 10‐item (Chinese translation)	Anxiety and depression

*Note:* A full reference list to Table 2 is presented in File S3.

### 4.3 Critical Appraisal of Sources of Evidence

Across the *n* = 386 included studies, a total of 15 instruments were identified. All instruments available in English were critically appraised using the checklist developed for this scoping review. Each instrument was assessed and scored based on the key attributes and known factors that influence nurse resilience, with higher scores indicating a more robust measure of nurse resilience (Table 3). Where multiple versions of an instrument existed due to the instrument being directly translated into other languages, the English version of the instrument was used for critical appraisal.

**TABLE 3:** Critical appraisal of instruments used to measure resilience.

Instrument	Social support	Self‐efficacy	Work‐life balance	Self‐care	Humour	Optimism	Being realistic	Workplace conditions	Organisational philosophy	Management performance	Team factors	Score/11
Adapted adult personal resilience scale	✘	✔	✘	✘	✘	✘	✘	✘	✘	✘	✘	1
Brief resilience scale	✘	✘	✘	✘	✘	✘	✘	✘	✘	✘	✘	0
Brief resilient coping scale	✘	✘	✘	✘	✘	✔	✘	✘	✘	✘	✘	1
Connor‐Davidson resilience scale 2‐item	✘	✘	✘	✘	✘	✘	✘	✘	✘	✘	✘	0
Connor‐Davidson resilience scale 10‐item	✘	✔	✘	✘	✔	✘	✘	✘	✘	✘	✘	2
Connor‐davidson resilience scale 25‐item	✔	✔	✘	✘	✔	✔	✔	✘	✘	✘	✘	5
Emergency nurse’ professional resilience tool	✔	✘	✔	✔	✘	✔	✘	✔	✘	✘	✔	6
Personal resilience tool	✘	✔	✘	✘	✘	✘	✘	✘	✘	✘	✘	1
Resilience at work scale	✔	✘	✔	✔	✘	✘	✔	✔	✘	✘	✘	5
Resilience at work team scale	✔	✘	✔	✔	✘	✔	✘	✔	✘	✘	✔	6
Response to stressful experiences scale	✘	✔	✘	✘	✘	✔	✔	✘	✘	✘	✘	3
Resilience scale 14‐item	✘	✔	✘	✘	✔	✘	✔	✘	✘	✘	✘	3
Resilience scale 25‐item	✘	✔	✘	✘	✔	✘	✔	✘	✘	✘	✘	3
Resilience scale for adults	✔	✔	✘	✘	✔	✔	✔	✘	✘	✘	✘	5
Workplace resiliency inventory	✔	✔	✘	✘	✘	✔	✘	✘	✘	✘	✘	3
Number of times measured across instruments	6	9	3	3	5	7	6	3	0	0	2	

### 4.4 Results of Individual Sources of Evidence

The scores for the instruments critically appraised ranged from 0 to 6 out of a total possible score of 11 (Table 3). The highest scoring instruments were the Emergency Nurse Professional Resilience Tool (Norouzinia et al. 2022) and the Resilience at Work Team Scale (McEwen and Boyd 2018). Both included six of the known key attributes and factors that influence nurse resilience.

### 4.5 Synthesis of Results

#### 4.5.1 Instrument Development

Of the 15 instruments identified only two instruments, the Emergency Nurse Professional Resilience Tool (Norouzinia et al. 2022) and the Personal Resilience Tool (Wei and Taormina 2014), were specifically developed to measure nurse resilience. The Emergency Nurse Professional Resilience Tool was developed to measure resilience in Iranian nurses working in emergency departments and was reported in a single study (Norouzinia et al. 2022). The Personal Resilience Tool was developed to measure resilience in hospital‐based Chinese nurses and administered in a single study (Wei and Taormina 2014). The other 13 instruments identified were developed using samples of patient populations (Connor and Davidson 2003; Friborg et al. 2003; Sinclair and Wallston 2004; Vaishnavi, Connor, and Davidson 2007), university students (Campbell‐Sills and Stein 2007; McLarnon and Rothstein 2013; Smith et al. 2008), general populations (Friborg et al. 2003; Wagnild 2009), employees in non‐specific workplaces (McEwen and Boyd 2018; Winwood, Colon, and McEwen 2013), community‐dwelling older adults (Wagnild and Young 1993), military personnel (Johnson et al. 2011) and rural doctors (Handoyo et al. 2021). The 13 instruments developed for other populations were later applied to samples of nurses in the studies included in this scoping review. Validity and reliability testing were reported in the development of all the instruments (File S4).

#### 4.5.2 Instrument Features

Self‐efficacy was the most commonly included attribute, featuring *n* = 9 instruments (Table 3). Optimism (*n* = 7), being realistic (*n* = 6) and social support (*n* = 6) were the other most commonly included attributes. None of the instruments measured organisational philosophy or management performance. The Brief Resilience Scale (Smith et al. 2008) and the 2‐item version of the Connor‐Davidson Resilience Scale (CD‐RISC) (Vaishnavi, Connor, and Davidson 2007) did not feature any of the known key attributes and factors that influence nurse resilience.

#### 4.5.3 Application of Instruments

The majority of studies administered a single instrument to measure resilience. Exceptions to this were one study where three different instruments to measure resilience were applied (Connelly et al. 2023) and three studies that used two instruments (Connelly et al. 2024; Janzarik et al. 2022; McCoy, Sauer, and Sha 2023) (Table 2). The 25‐item CD‐RISC (Connor and Davidson 2003) was the most commonly applied instrument, applied in 45% (*n* = 175) of the included studies, followed by the abbreviated 10‐item CD‐RISC (Campbell‐Sills and Stein 2007) applied in 23% (*n* = 89) of studies (Table 4). Typically, other measures were collected alongside instruments to measure resilience including burnout, stress, depression and anxiety.

**TABLE 4:** Frequency of instrument use.

Instrument	Frequency
Adapted adult personal resilience Scale (Handoyo et al. 2021)	1
Brief resilience scale (Smith et al. 2008)	53
Brief resilient coping scale (Sinclair and Wallston 2004)	9
Connor‐davidson resilience scale 2‐item (Vaishnavi, Connor, and Davidson 2007)	4
Connor‐davidson resilience scale 10‐item (Campbell‐Sills and Stein 2007)	89
Connor‐davidson resilience scale 25‐item (Connor and Davidson 2003)	175
Emergency nurse’ professional resilience tool (Norouzinia et al. 2022)	1
Personal resilience tool (Wei and Taormina 2014)	1
Resilience at work scale (Winwood, Colon, and McEwen 2013)	10
Resilience at work team scale (McEwen and Boyd 2018)	1
Response to stressful experiences scale (Johnson et al. 2011)	1
Resilience scale 14‐item (Wagnild 2009)	20
Resilience scale 25‐item (Wagnild and Young 1993)	10
Resilience scale for adults (Friborg et al. 2003)	14
Workplace resiliency inventory (McLarnon and Rothstein 2013)	2
Total	391^a^

^a^ Four studies used multiple resilience instruments.

## 5 Discussion

The aim of this scoping review was to identify and critically appraise instruments that have been used to measure nurse resilience. The volume of research including measures of nurse resilience has steadily increased from 2012 to 2020. In the context of the coronavirus pandemic, the number of studies measuring nurse resilience exponentially increased. This marked increase reflects growing concerns about global nursing shortages that have been worsened by the added pressures of the pandemic (Buchan, Catton, and Shaffer 2022). The wide range of countries from which studies originated, highlights international recognition of the importance of nurse resilience.

Whilst there is urgency in practice to find ways to sustain and retain nurses in the profession (Kim and Chang 2022), the vast majority of studies identified in the scoping review were observational in nature and did not measure workplace factors. The predominately cross‐sectional studies that were conducted provide some insight into nurse resilience however, this saturation of non‐interventional research has not addressed the pressing issues healthcare systems face as nursing shortages continue to increase globally (Buchan and Catton 2023). Coupled with how nurse resilience has been measured in research to date, this leaves substantial limitations in our understanding of nurse resilience and how to promote resilience to ensure safe patient care.

The critical appraisal of the 15 instruments used to measure nurse resilience in the studies included in the scoping review revealed significant deficits in the existing instruments. In keeping with the criticisms levelled at research investigating nurse resilience (Taylor 2019; Virkstis, Herleth, and Langr 2018) the instruments predominately focused on individual factors and largely failed to consider the impact of the work environment. In the post‐COVID era, the nursing workforce is stretched more thinly than ever, and the workplace is even more chaotic as a result. The instruments applied to measure nurse resilience do not account for the current context the nursing profession finds itself in. Nurse resilience now more than ever, is impacted by both individual and external factors. The failure to consider the impact of the work environment was particularly evident with none of the instruments measuring the effect of organisational philosophy or management performance on nurse resilience. Even the most robust of the instruments (Norouzinia et al. 2022) only captured 55% (*n* = 6) of the key attributes and factors that are known to influence nurse resilience. Most of the instruments applied (*n* = 13) were not specifically designed to measure nurse resilience, and therefore failed to capture the complexity of the attributes and factors of nurse resilience.

The two instruments that were specifically developed to measure resilience in samples of nurses did not outperform instruments developed for other populations (Norouzinia et al. 2022; Wei and Taormina 2014). The Personal Resilience Tool (Wei and Taormina 2014) had one of the lowest critical appraisal scores, whereas the Emergency Nurse Professional Resilience Tool (Norouzinia et al. 2022) was one of the more robust instruments. These limitations even in the nurse‐specific instruments seem to be reflective of an influence from the wider literature where resilience is largely considered an individual issue (Cooper, Brown, and Leslie 2021b). Despite reported attempts to create instruments specific to nurse resilience (Norouzinia et al. 2022; Wei and Taormina 2014), a comprehensive instrument that incorporates both internal and external factors is missing from the literature.

The inclusion of multiple instruments to measure nurse resilience in some recent studies (Connelly et al. 2023), represents an awareness by some researchers of the limitations of current measures of resilience. Administering multiple instruments appears to be an attempt to address the shortcomings of existing instruments and better measure nurse resilience. The combination of three instruments to measure resilience (Connor and Davidson 2003; McEwen and Boyd 2018; Winwood, Colon, and McEwen 2013) by Connelly et al. (2023) meant that nine of the 11 attributes and factors influence nurse resilience were captured. Combining two instruments led to seven of the 11 attributes of resilience being included in Connelly et al. (2024) study. Whilst combining the instruments addressed some shortcomings, administering three instruments to measure the same variable is burdensome and inefficient for researchers and research participants. The combination of two measures of resilience resulted in no net gain in terms of the number of attributes and factors that influence nurse resilience included in the Janzarik et al. (2022) and McCoy, Sauer, and Sha (2023) studies. Given that none of the appraised instruments feature organisational philosophy or management performance, no combination of these instruments would include all the key attributes and factors that influence nurse resilience, further highlighting the need for the development of a specific and comprehensive measure that includes all the key attributes of nurse resilience for application in one tool instrument in future research and practice.

### 5.1 Limitations

The scoping review methodology facilitated an exploration of how nurse resilience has been measured, however, there were some limitations to this review. In keeping with the methodology of a scoping review no critical appraisal was conducted regarding the quality of the included studies. However, a critical appraisal of the instruments used in the included studies was conducted. The instruments included in the review were limited to those available in English.

## 6 Conclusion

A variety of instruments have been used to measure resilience in samples of nurses. There are significant deficiencies in the instruments, identified through a critical appraisal approach. None of the instruments included all of the key attributes and factors that influence nurse resilience. Notably, instruments predominately focused on individual factors, did not consider the impact of the work environment and failed to capture the complexity of nurse resilience. Therefore, the existing instruments are ineffective in measuring nurse resilience and are unlikely to promote a full understanding of nurse resilience or support the development of optimal interventions to sustain nurse resilience. In the context of a growing interest in the phenomenon of nurse resilience in the post‐COVID era, a profession‐specific comprehensive measure of nurse resilience needs to be developed to address the current gaps identified in this scoping review.

## Conflicts of Interest

The authors declare no conflicts of interest.

## Peer Review

The peer review history for this article is available at https://www.webofscience.com/api/gateway/wos/peer‐review/10.1111/jan.16769.

## Supporting information


File S1.

File S2.

File S3.

File S4.

## Data Availability

Data sharing not applicable to this article as no datasets were generated or analysed during the current study.
