# Supplementary material for: A Scoping Review of Instruments Used to Measure Resilience in Samples of Nurses
Source: J Adv Nurs. 2025 Feb 7;81(9):5718–62. doi: 10.1111/jan.16769 (PMC12371848; doi:10.1111/jan.16769)
Supplement: Supplementary file 2 — File S2. [file JAN-81-5718-s001.docx]

**Supplementary File 2.** Search strategy

| **Database** | **Limitations** | **Concept 1** | **Concept 2** | **Concept 3** | **Concept 4** | **Date range** | **Search date** | **Total results** |
| --- | --- | --- | --- | --- | --- | --- | --- | --- |
| CINAHL Ultimate | Limited to texts with available abstracts, peer reviewed and in English | Nurse or nursing or nurses (AB) | Resilience or resiliency or resilient (AB) | Measur* or tool* or scale* or instrument* or longitudinal or cross-sectional (AB) | NOT Qualitative (TI) | 2012 – 2024 | 09/12/2024 | 1116 |
| MEDLINE | Limited to texts with available abstracts, and in English | Nurse or nursing or nurses (AB) | Resilience or resiliency or resilient (AB) | Measur* or tool* or scale* or instrument* or longitudinal or cross-sectional (AB) | NOT Qualitative (TI) | 2012 – 2024 | 09/12/2024 | 1611 |
| PsychINFO | Limited to texts with available abstracts, and in English | Nurse or nursing or nurses (AB) | Resilience or resiliency or resilient (AB) | Measur* or tool* or scale* or instrument* or longitudinal or cross-sectional (AB) | NOT Qualitative (TI) | 2012 – 2024 | 09/12/2024 | 552 |
| Emcare | Limited to texts with available abstracts, and in English | Nurse or nursing or nurses (AB) | Resilience or resiliency or resilient (AB) | Measur* or tool* or scale* or instrument* or longitudinal or cross-sectional (AB) | NOT Qualitative (TI) | 2012 – 2024 | 09/12/2024 | 1415 |
