# Supplementary material for: A Scoping Review of Instruments Used to Measure Resilience in Samples of Nurses
Source: J Adv Nurs. 2025 Feb 7;81(9):5718–62. doi: 10.1111/jan.16769 (PMC12371848; doi:10.1111/jan.16769)
Supplement: Supplementary file 3 — File S3. [file JAN-81-5718-s004.docx]

**Supplementary File 3.** Table 2 reference list

Abdollahi, R., Iranpour, S., & Ajri-Khameslou, M. (2021). Relationship between resilience and professional moral courage among nurses. *Journal of Medical Ethics and History of Medicine*,*14*(3). https://doi.org/10.18502/jmehm.v14i3.5436

Abdulmohdi, N. (2024). The relationships between nurses' resilience, burnout, perceived organisational support and social support during the second wave of the COVID-19 pandemic: A quantitative cross-sectional survey. *Nursing Open*, *11*(1), e2036. https://doi.org/doi:https://dx.doi.org/10.1002/nop2.2036

Abu-Alhaija, D. M., & Gillespie, G. L. (2022). Critical clinical events and resilience among emergency nurses in 3 trauma hospital-based emergency departments: A cross-sectional study. *Journal of Emergency Nursing*,*48*(5), 525-537. https://doi.org/10.1016/j.jen.2022.05.001

Abualruz, H., & Hayajneh, F. (2023). Effectiveness of a theory-based resiliency intervention for nurses. T*he Journal of Continuing Education in Nursing*, *54*(12), 581-588. <https://doi.org/doi:https://dx.doi.org/10.3928/00220124-20231013-03>

Abualruz, H., Hayajneh, F., Othman, E. H., Abu Sabra, M. A., Khalil, M. M., Khalifeh, A. H.,…Abousoliman, A. D. (2024a). The relationship between emotional intelligence, resilience, and psychological distress among nurses in Jordan. *Archives of Psychiatric Nursing, 51*, 108-113. <https://doi.org/doi:10.1016/j.apnu.2024.05.014>

Abualruz, H., Rayan, A., Al-Ghabeesh, S., Fawaz, M., Jaafeer, R., Qutami, B., & Alyami, H. (2024b). The role of psychological factors on improving work engagement among nurses. *Frontiers in Psychology, 15*, 1419855. <https://doi.org/doi:https://dx.doi.org/10.3389/fpsyg.2024.1419855>

Afshari, D., Nourollahi-Darabad, M., & Chinisaz, N. (2021). Demographic predictors of resilience among nurses during the COVID-19 pandemic. *Work*,*68*(2), 297-303. <https://doi.org/10.3233/WOR-203376>

Aghamohammadi, F., Saed, O., & Dinmohammadi, M. (2023). Factors Affecting the Resilience of Iranian Nurses During the COVID-19 Pandemic. J*ournal of Client Centered Nursing Care, 9*(3), 223-230. <https://doi.org/doi:10.32598/JCCNC.9.3.451>

Akbulut, S., Boz, G., Gokce, A., Unsal, S., Saritas, H., Kizilay, E., . . . Colak, C. (2023). Evaluation of nurses' vaccine hesitancy, psychological resilience, and anxiety levels during COVID-19 pandemic. *The Eurasian Journal of Medicine*,*55*(2), 140-145. <https://doi.org/10.5152/eurasianjmed.2023.22162>

Akinabadi, A. S., Khari, S., Looha, M. A., & Zandi, M. (2024). Correlations among family functions, lifestyles and resilience of nurses working in COVID-19 intensive care units in post-corona era. *International Journal of Healthcare Management*. <https://doi.org/doi:https://dx.doi.org/10.1080/20479700.2024.2346878>

Al Hadid, L. A. E., Al Barmawi, M. A., Alnjadat, R., & Farajat, L. A. (2022). The impact of stress associated with caring for patients with COVID-19 on career decisions, resilience, and perceived self-efficacy in newly hired nurses in Jordan: A cross-sectional study. *Health Science Reports*,*5*(6). https://doi.org/10.1002/hsr2.899

Alameddine, M., Bou-Karroum, K., Ghalayini, W., & Abiad, F. (2021a). Resilience of nurses at the epicenter of the COVID-19 pandemic in Lebanon. *International Journal of Nursing Sciences*,*8*(4), 432-438. https://doi.org/10.1016/j.ijnss.2021.08.002

Alameddine, M., Clinton, M., Bou-Karroum, K., Richa, N., & Doumit, M. A. A. (2021b). Factors associated with the resilience of nurses during the COVID-19 pandemic. *Worldviews on Evidence-Based Nursing*,*18*(6), 320-331. https://doi.org/10.1111/wvn.12544

Alan, H., Eskici, G. T., Sen, H. T., & Bacaksiz, F. E. (2022). Nurses' disaster core competencies and resilience during the COVID-19 pandemic: A cross-sectional study from Turkey. *Journal of Nursing Management*,*30*(3), 622-632. https://doi.org/10.1111/jonm.13552

Albaqawi, H. M., & Alshammari, M. H. (2024). Resilience, compassion fatigue, moral distress and moral injury of nurses. *Nursing Ethics*, 9697330241287862. <https://doi.org/doi:https://dx.doi.org/10.1177/09697330241287862>

Albougami, A. (2024). Resilient coping levels and psychometric properties of the Brief Resilient Coping Scale among nursing professionals in Saudi Arabia. *Healthcare*, 12(21). <https://doi.org/doi:https://dx.doi.org/10.3390/healthcare12212181>

Alenezi, A. (2024). The impact of resilience on workplace violence experienced by mental health nurses: A cross-sectional survey. *Journal of Nursing Management*, 1-10. <https://doi.org/doi:10.1155/2024/4449445>

Alharbi, J., Jackson, D., & Usher, K. (2020). Personal characteristics, coping strategies, and resilience impact on compassion fatigue in critical care nurses: A cross-sectional study. *Nursing & Health Sciences*,*22*(1), 20-27. https://doi.org/10.1111/nhs.12650

Al-Harrasi, S., Al Sabei, S., Al Omari, O., & Al Abrawi, U. (2024). Nurses' job burnout and resilience in neonatal intensive care units. *Journal of Perinatal & Neonatal Nursing, 38*(2), 201-211. <https://doi.org/doi:10.1097/JPN.0000000000000817>

Alhawatmeh, H., Alsholol, R., Dalky, H., Al-Ali, N., & Albataineh, R. (2021). Mediating role of resilience on the relationship between stress and quality of life among Jordanian registered nurses during COVID-19 pandemic. *Heliyon*,*7*(11). https://doi.org/10.1016/j.heliyon.2021.e08378

Almegewly, W., Alhejji, A., Alotaibi, L., Almalki, M., Alanezi, M., Almotiri, A., . . . Albarakah, A. (2022). Perceived stress and resilience levels during the COVID-19 pandemic among critical care nurses in Saudi Arabia: A correlational cross-sectional study. *PeerJ*,*10*. https://doi.org/10.7717/peerj.13164

Alonazi, O., Alshowkan, A., & Shdaifat, E. (2023). The relationship between psychological resilience and professional quality of life among mental health nurses: A cross-sectional study. *BMC Nursing*,*22*(1). https://doi.org/10.1186/s12912-023-01346-1

Al-Shomrani, S., Mahran, S. M., & Felemban, O. (2024). The relationship between resilience and the intention to leave among staff nurses at governmental hospitals in the Al-Baha region of Saudi Arabia. *Cureus, 16*(3), e56699. <https://doi.org/doi:https://dx.doi.org/10.7759/cureus.56699>

Andersen, S., Mintz-Binder, R., Sweatt, L., & Song, H. (2021). Building nurse resilience in the workplace. *Applied Nursing Research*,*59*(1). https://doi.org/10.1016/j.apnr.2021.151433

Ang, S. Y., Hemsworth, D., Uthaman, T., Ayre, T. C., Mordiffi, S. Z., Ang, E., & Lopez, V. (2018a). Understanding the influence of resilience on psychological outcomes: Comparing results from acute care nurses in Canada and Singapore. *Applied Nursing Research*,*43*, 105-113. https://doi.org/10.1016/j.apnr.2018.07.007

Ang, S. Y., Uthaman, T., Ayre, T. C., Mordiffi, S. Z., Ang, E., & Lopez, V. (2018b). Association between demographics and resilience: A cross-sectional study among nurses in Singapore. *International Nursing Review*,*65*(3), 459-466. https://doi.org/10.1111/inr.12441

Aqtam, I., Ayed, A., Toqan, D., Salameh, B., Elhay, E. S. A., Zaben, K., & Shouli, M. M. (2023). The relationship between stress and resilience of nurses in intensive care units during the COVID-19 pandemic. *Inquiry*,*60*(1). https://doi.org/10.1177/00469580231179876

Asadi, Y., Molazem, Z., Mohebbi, Z., & Ghaemmaghami, P. (2023). Investigating the relationship between resilience and professional ethics in nurses: a cross-sectional study in southern Iran. *BMC Nursing, 22*(1), 1-8. <https://doi.org/doi:10.1186/s12912-023-01578-1>

Ata, E. E., Yilmaz, E. B., & Bayrak, N. G. (2020). Resilience, occupational satisfaction, burnout and compassion fatigue in Turkish intensive care nurses. *Kontakt: Journal of Nursing and Social Sciences Related to Health and Illness*,*22*(3), 152-158. https://doi.org/10.32725/kont.2020.028

Atay, N., Sahin-Bayindir, G., Buzlu, S., Koc, K., & Kuyuldar, Y. (2023). The relationship between posttraumatic growth and psychological resilience of nurses working at the pandemic clinics. *International Journal of Nursing Knowledge*,*34*(3), 226-235. https://doi.org/10.1111/2047-3095.12397

Babanataj, R., Mazdarani, S., Hesamzadeh, A., Gorji, M. H., & Cherati, J. Y. (2019). Resilience training: Effects on occupational stress and resilience of critical care nurses. *International Journal of Nursing Practice*,*25*(1). https://doi.org/10.1111/ijn.12697

Bai, C., & Bai, B. (2024). Personal strength use and job satisfaction in Chinese nurses: The mediating roles of basic psychological needs satisfaction and resilience. *International Nursing Review, 71*(3), 580-587. <https://doi.org/doi:10.1111/inr.12877>

Bai, C., Bai, B., Zhang, H., Kong, F., & Wang, S. (2024). How does gratitude relate to nurses' job satisfaction? The mediating role of resilience and stress. *Worldviews on Evidence-Based Nursing, 21*(2), 120-127. <https://doi.org/doi:https://dx.doi.org/10.1111/wvn.12710>

Bani, M., Russo, S., Cardinale, C., Ardenghi, S., Rampoldi, G., Luciani, M., . . . Strepparava, M. G. (2023). "Jumping into the COVID-19 arena": The professional transition into clinical practice of new graduate nurses in Italy at time of COVID-19. *Journal of Clinical Nursing*,*32*(13), 3898-3908. https://doi.org/10.1111/jocn.16554

Bernburg, M., Groneberg, D. A., & Mache, S. (2019). Mental health promotion intervention for nurses working in German psychiatric hospital departments: A pilot study. *Issues in Mental Health Nursing*,*40*(8), 706-711. https://doi.org/10.1080/01612840.2019.1565878

Bonamer, J. R., & Aquino-Russell, C. (2019). Self-care strategies for professional development: Transcendental meditation reduces compassion fatigue and improves resilience for nurses. *Journal for Nurses in Professional Development*,*35*(2), 93-97. https://doi.org/10.1097/NND.0000000000000522

Bouchard, L., & Rainbow, J. (2021). Compassion fatigue, presenteeism, Adverse Childhood Experiences (ACES), and resiliency levels of Doctor of Nursing Practice (DNP) students. *Nurse Education Today*,*100*(1). <https://doi.org/10.1016/j.nedt.2021.104852>

Brook, J., Duguid, B., & Miller, N. (2023). Symptoms of post-traumatic stress disorder in early career nurses during the COVID-19 pandemic: A longitudinal survey study. *Journal of Clinical Nursing, 32*(23), 8063-8077. <https://doi.org/doi:https://dx.doi.org/10.1111/jocn.16879>

Brown, R., Wey, H., & Foland, K. (2018). The relationship among change fatigue, resilience, and job satisfaction of hospital staff nurses. *Journal of Nursing Scholarship*,*50*(3), 306-313. https://doi.org/10.1111/jnu.12373

Buntoro, I. F., Folamauk, C. L., Nurina, R. L., Kleden, S. S., & Handoyo, N. E. (2023). Resilience, depression and their effect on nurse retention: A survey in rural Indonesia. *Rural and Remote Health*,*23*(3). https://doi.org/10.22605/RRH7725

Bursch, B., Emerson, N. D., Arevian, A. C., Aralis, H., Galuska, L., Bushman, J., . . . Bulut, Y. (2018). Feasibility of online mental wellness self-assessment and feedback for pediatric and neonatal critical care nurses. *Journal of Pediatric Nursing*,*43*, 62-68. https://doi.org/10.1016/j.pedn.2018.09.001

Byers, O. M., Fitzpatrick, J. J., McDonald, P. E., & Nelson, G. C. (2021). Giving while grieving: Racism-related stress and psychological resilience in Black/African American registered nurses. *Nursing Outlook*,*69*, 1039-1048. https://doi.org/10.1016/j.outlook.2021.05.010

Cabrera-Aguilar, E., Zevallos-Francia, M., Morales-Garcia, M., Ramirez-Coronel, A. A., Morales-Garcia, S. B., Sairitupa-Sanchez, L. Z., & Morales-Garcia, W. C. (2023). Resilience and stress as predictors of work engagement: the mediating role of self-efficacy in nurses. *Frontiers in Psychiatry*, *14*, 1202048. <https://doi.org/doi:https://dx.doi.org/10.3389/fpsyt.2023.1202048>

Cao, X., & Chen, L. (2019). Relationships among social support, empathy, resilience and work engagement in haemodialysis nurses. *International Nursing Review*,*66*(3), 366-373. https://doi.org/10.1111/inr.12516

Cao, X., & Chen, L. (2020). The impact of empathy on work engagement in hemodialysis nurses: The mediating role of resilience. *Japan Journal of Nursing Science*,*17*(1). https://doi.org/10.1111/jjns.12284

Cao, X., & Chen, L. (2021). Relationships between resilience, empathy, compassion fatigue, work engagement and turnover intention in haemodialysis nurses: A cross-sectional study. *Journal of Nursing Management*,*29*(5), 1054-1063. https://doi.org/10.1111/jonm.13243

Cao, X., Li, J., & Gong, S. (2021a). Effects of resilience, social support, and work environment on turnover intention in newly graduated nurses: The mediating role of transition shock. *Journal of Nursing Management*,*29*(8), 2585-2593. https://doi.org/doi:10.1111/jonm.13418

Cao, X., Li, J., & Gong, S. (2021b). The relationships of both transition shock, empathy, resilience and coping strategies with professional quality of life in newly graduated nurses. *BMC Nursing*,*20*(1). https://doi.org/10.1186/s12912-021-00589-0

Cao, Q., Wu, H., Tang, X., Zhang, Q., & Zhang, Y. (2024). Effect of occupational stress and resilience on insomnia among nurses during COVID-19 in China: a structural equation modelling analysis. *BMJ Open, 14*(7), e080058. <https://doi.org/doi:https://dx.doi.org/10.1136/bmjopen-2023-080058>

Caroccini, T. P., Neves, V. R., Silva, S. M. d., Belasco, A. G. S., Baptista, P. C. P., & Balsanelli, A. P. (2024). Resilience and the meaning of nurses' work in the COVID-19 pandemic. *Acta Paulista de Enfermagem, 37*(1), 1-9. <https://doi.org/doi:10.37689/acta-ape/2024AO000027244>

Carpio, R. C., Castro, L. P., Huerto, H. M., Highfield, M. E. F., & Mendelson, S. (2018). Exploring resilience at work among first-line nurse managers. *Journal of Nursing Administration*,*48*(10), 481-486. https://doi.org/10.1097/NNA.0000000000000655

Catarelli, B., Nobles, P., Aull, M., & Yi, F. (2023). Evaluating burnout and resiliency in new graduate nurses: A cross-sectional study. *Journal of Nursing Administration*,*53*(5), 259-265. https://doi.org/10.1097/NNA.0000000000001279

Celik, A. S., & Yarali, S. (2023). The effect of laughter yoga on the psychological resilience and sleep quality of nurses during the pandemic: A randomized controlled trial. *Alternative Therapies in Health and Medicine*,*29*(5), 146-152. https://doi.org/10.1016/j.ctcp.2022.101637

Cha, C., & Baek, G. (2023). Factors influencing the burnout dimensions among nurses: A cross-sectional study in South Korea. *Nursing Open, 10*(12), 7725-7737. https://doi.org/doi:https://dx.doi.org/10.1002/nop2.2013

Chan, S., Takemura, N., Chau, P., Lin, C., & Wang, M. (2021). Psychological impact of the COVID-19 pandemic on licensed full-time practicing nurses undertaking part-time studies in higher education: A cross-sectional study. *International journal of environmental research and public health*,*18*(16). https://doi.org/10.3390/ijerph18168569

Chen, S., Yan, S., Zhao, W., Gao, Y., Zong, W., Bian, C., . . . Zhang, Y. (2022). The mediating and moderating role of psychological resilience between occupational stress and mental health of psychiatric nurses: A multicenter cross-sectional study. *BMC psychiatry*,*22*(1). https://doi.org/10.1186/s12888-022-04485-y

Chen, X., Li, Q., Xu, F., & Han, B. (2021). The mediating role of resilience between work-family conflict and career development among Chinese nurses: A cross-sectional study. *Journal of Nursing Management*,*29*(6), 1733-1741. https://doi.org/10.1111/jonm.13323

Chen, M., Su, R., Hu, M., Huang, X., Wu, B., Zhou, L.,…Feng, P. (2024a). Psychological resilience and associated factors in caring for mass burn patients among rescue nurses: A cross-sectional study. *Burns*. <https://doi.org/doi:https://dx.doi.org/10.1016/j.burns.2024.07.022>

Chen, M., Weng, Y., Zhang, J., Gu, L., Chen, W., Qiao, M.,…Zhang, L. (2023). Factors associated with nurses' attitudes for providing oral care in geriatric care facilities: a cross-sectional study. *BMC Oral Health, 23*(1), 1-8. <https://doi.org/doi:10.1186/s12903-023-03517-7>

Chen, M., Xie, H., Liao, X., & Ni, J. (2024b). Workplace violence and turnover intention among Chinese nurses: the mediating role of compassion fatigue and the moderating role of psychological resilience. *BMC Public Health, 24*(1), 2437. <https://doi.org/doi:https://dx.doi.org/10.1186/s12889-024-19964-y>

Chen, Z., Li, R., Zhao, X., Li, Z., Zhou, S., & Zhang, K. (2024c). Relationship between resilience, emergency response capacity, and occupational stressors of new nurse during the re-outbreak of COVID-19 in China. *Disaster Medicine and Public Health Preparedness, 18,* e285. <https://doi.org/doi:https://dx.doi.org/10.1017/dmp.2024.157>

Chesak, S. S., Bhagra, A., Schroeder, D. R., Foy, D. A., Cutshall, S. M., & Sood, A. (2015). Enhancing resilience among new nurses: Feasibility and efficacy of a pilot intervention. *The Ochsner Journal*,*15*(1), 38-44. https://www.ncbi.nlm.nih.gov/pmc/articles/PMC4365845/

Chesak, S. S., Morin, K. H., Cutshall, S. M., Jenkins, S. M., & Sood, A. (2021). Feasibility and efficacy of integrating resiliency training into a pilot nurse residency program. *Nurse Education in Practice*,*50*. https://doi.org/10.1016/j.nepr.2020.102959

Chiu-Yueh, Y., Mu-Hsi, Y., & Beckstead, J. (2024). Psychometric properties of the Chinese version of the Gratitude at Work Scale in employed nurses: A cross-sectional Study. *Journal of Nursing Research, 32*(4), 1-11. https://doi.org/doi:10.1097/jnr.0000000000000620

Cho, G. J., & Kang, J. (2017). Type D personality and post-traumatic stress disorder symptoms among intensive care unit nurses: The mediating effect of resilience. *PLoS ONE*, *12*(4). https://doi.org/10.1371/journal.pone.0175067

Choi, D. S., & Kim, S. H. (2022). Factors affecting occupational health of shift nurses: Focusing on job stress, health promotion behavior, resilience, and sleep disturbance. *Safety and Health at Work*,*13*(1), 3-8. https://doi.org/10.1016/j.shaw.2021.09.001

Choi, S., Kim, H., & Park, K. (2022). Experience of violence and factors influencing response to violence among emergency nurses in South Korea: Perspectives on stress-coping theory. *Journal of Emergency Nursing*,*48*(1), 74-87. <https://doi.org/10.1016/j.jen.2021.07.008>

Chua, M. M., Ang, W. H. D., Siew, A. L., & Chen, H.-C. (2024). Personal and workplace characteristics as predictors of intent-to-stay among registered nurses: An exploratory quantitative multicentre study. *Journal of Advanced Nursing*. <https://doi.org/doi:https://dx.doi.org/10.1111/jan.16459>

Chukwuorji, J. C., Aluma, L. C., Ibeagha, P. N., Eze, J. E., Agbo, A. A., Muomah, R. C.,…Zacchaeus, E. A. (2024). Spirituality, resilience and vicarious posttraumatic growth among orthopedic nurses in Nigeria. *Journal of Religion and Health.* <https://doi.org/doi:https://dx.doi.org/10.1007/s10943-024-02167-5>

Chura, S., Saintila, J., Mamani, R., Mamani, P. G. R., & Morales-Garcia, W. C. (2022). Predictors of depression in nurses during COVID-19 health emergency; The mediating role of resilience: A cross-sectional study. *Journal of Primary Care and Community Health*, *13*. https://doi.org/10.1177/21501319221097075

Clark, P., Crawford, T. N., Hulse, B., & Polivka, B. J. (2021). Resilience, moral distress, and workplace engagement in emergency department nurses. *Western Journal of Nursing Research*,*43*(5), 442-451. https://doi.org/10.1177/0193945920956970

Coetzee, B., Haine, P., Kidd, M., Shongwe, L., Janse Van Vuuren, M., & Kagee, A. (2024). Coping in crisis: The role of sense of coherence, life satisfaction, and resilience in the relationship between depression, social support, fear of COVID-19, and perceived vulnerability to disease among nurses in South Africa. *Journal of Health Psychology*. https://doi.org/doi:https://dx.doi.org/10.1177/13591053241279000

Connelly, D. M., Snobelen, N., Garnett, A., Guitar, N., Flores-Sandoval, C., Sinha, S., . . . Smith-Carrier, T. (2023). Report on fraying resilience among the Ontario registered practical nurse workforce in long-term care homes during COVID-19. *Nursing Open*,*10*(7), 4359-4372. <https://doi.org/10.1002/nop2.1678>

Connelly, D. M., Guitar, N. A., Garnett, A., Smith-Carrier, T., Prentice, K., Calver, J.,…Snobelen, N. (2024). A leave of absence might not be a bad thing: Registered practical nurses working in home care during the COVID-19 pandemic. *Home Health Care Management & Practice, 36*(4), 334-344. https://doi.org/doi:10.1177/10848223241232408

Converso, D., Sottimano, I., Guidetti, G., Loera, B., Cortini, M., & Viotti, S. (2018). Aging and work ability: The moderating role of job and personal resources. *Frontiers in Psychology*, *8*. https://doi.org/10.3389/fpsyg.2017.02262

Converso, D., Viotti, S., Sottimano, I., Loera, B., Molinengo, G., & Guidetti, G. (2019). The relationship between menopausal symptoms and burnout. A cross-sectional study among nurses. *BMC Women's Health*,*19*(1), 1-6. https://doi.org/10.1186/s12905-019-0847-6

Cooper, A. L., Brown, J. A., & Leslie, G. D. (2021). The impact of organisational values on nurse resilience: A mixed-methods study. *Journal of Nursing Management*,*29*(7), 2074-2083. https://doi.org/10.1111/jonm.13338

Craigie, M., Slatyer, S., Hegney, D., Osseiran-Moisson, R., Gentry, E., Davis, S., . . . Rees, C. (2016). A pilot evaluation of a mindful self-care and resiliency (MSCR) intervention for nurses. *Mindfulness*, *7*(3), 764-774. https://doi.org/10.1007/s12671-016-0516-x

Cuhadar, D., Bahar, A., Samancioglu Baglama, S., Kocak, H. S., & Ozkaya, M. (2023). Psychological resilience and perceived stress level in nurses: Experience of nurses in turkey. *Disaster Medicine and Public Health Preparedness*,*17*(15). https://doi.org/10.1017/dmp.2022.294

Dehvan, F., Kamangar, P., Baiezeedy, S., Roshani, D., & Ghanei-Gheshlagh, R. (2018). The relationship of mental health with resilience among psychiatric nurses. *Nursing Practice Today*,*5*(4), 368-374. https://doi.org/https://doi.org/10.18502/npt.v5i4.115

Delaney, M. C. (2018). Caring for the caregivers: Evaluation of the effect of an eight-week pilot Mindful Self-Compassion (MSC) training program on nurses' compassion fatigue and resilience. *PLoS ONE*,*13*(11). https://doi.org/10.1371/journal.pone.0207261

Delgado, C., Roche, M., Fethney, J., & Foster, K. (2020). Workplace resilience and emotional labour of Australian mental health nurses: Results of a national survey. *International Journal of Mental Health Nursing*,*29*(1), 35-46. https://doi.org/10.1111/inm.12598

Delgado, C., Roche, M., Fethney, J., & Foster, K. (2021). Mental health nurses' psychological well-being, mental distress, and workplace resilience: A cross-sectional survey. *International Journal of Mental Health Nursing*,*30*(5), 1234-1247. https://doi.org/10.1111/inm.12874

Ding, C., Li, L., Li, G., Li, X., Xie, L., & Duan, Z. (2023). Impact of workplace violence against psychological health among nurse staff from Yunnan-Myanmar Chinese border region: Propensity score matching analysis. *BMC Nursing*,*22*(1). https://doi.org/10.1186/s12912-023-01402-w

Dino, M. J. S., Bracero, P. J. B., Buencamino, A., Cajayon, S., Catajan, M. W. Q., De Leon, M. L. L., . . . Uayan, M. L. (2022). 'Should I stay or should I go?': A mixed methods study on nurse retention during challenging times. *Belitung Nursing Journal*,*8*(6), 481-490. https://doi.org/10.33546/BNJ.2327

Dolan, G., Strodl, E., & Hamernik, E. (2012). Why renal nurses cope so well with their workplace stressors. *Journal of Renal Care*,*38*(4), 222-232. https://doi.org/10.1111/j.1755-6686.2012.00319.x

Dordunoo, D., An, M., Chu, M. S., Yeun, E. J., Hwang, Y. Y., Kim, M., & Lee, Y. (2021). The impact of practice environment and resilience on burnout among clinical nurses in a tertiary hospital setting. *International Journal of Environmental Research and Public Health*, *18*(5). <https://doi.org/10.3390/ijerph18052500>

Du, X., Song, Y., Wang, H., Chen, W., Wang, L., Feng, X.,…Song, C. (2024). Research on the relationship between nurses' compliance with standard precautions, servant leadership and emotional exhaustion: A cross-sectional survey. *Journal of Advanced Nursing, 80*(7), 2822-2834. <https://doi.org/doi:https://dx.doi.org/10.1111/jan.16020>

Durmuş, A., Ünal, Ö., Türktemiz, H., & Öztürk, Y. E. (2024). The effect of nurses' perceived workplace incivility on their presenteeism and turnover intention: The mediating role of work stress and psychological resilience. *International Nursing Review, 71*(4), 960-968. <https://doi.org/doi:10.1111/inr.12950>

Ediz, C., & Yanik, D. (2024). Disaster preparedness perception, pyschological resiliences and empathy levels of nurses after 2023 Great Turkiye earthquake: Are nurses prepared for disasters: A risk management study. *Public Health Nursing, 41*(1), 164-174. <https://doi.org/doi:https://dx.doi.org/10.1111/phn.13267>

Fan, S., An, W., Zeng, L., Liu, J., Tang, S., Chen, J., & Huang, H. (2022). Rethinking "zero tolerance": A moderated mediation model of mental resilience and coping strategies in workplace violence and nurses' mental health. *Journal of Nursing Scholarship*,*54*(4), 501-512. <https://doi.org/10.1111/jnu.12753>

Foli, K. J., Wang, Y., Adams, N., & Coto, J. (2024). A middle-range theory of nurses in recovery from substance use. *Journal of Nursing Regulation, 15*(3), 33-46. https://doi.org/doi:10.1016/S2155-8256(24)00073-5

Foster, K., Shochet, I., Wurfl, A., Roche, M., Maybery, D., Shakespeare-Finch, J., & Furness, T. (2018). On PAR: A feasibility study of the Promoting Adult Resilience programme with mental health nurses. *International Journal of Mental Health Nursing*,*27*(5), 1470-1480. <https://doi.org/10.1111/inm.12447>

Foster, K., Shakespeare-Finch, J., Shochet, I., Maybery, D., Bui, M. V., Steele, M., & Roche, M. (2024a). Psychological distress, well-being, resilience, posttraumatic growth, and turnover intention of mental health nurses during COVID-19: A cross-sectional study. *International Journal of Mental Health Nursing, 33*(5), 1543 EP - 1552. <https://doi.org/doi:https://dx.doi.org/10.1111/inm.13354>

Foster, K., Shochet, I., Shakespeare-Finch, J., Maybery, D., Bui, M. V., Gordon, I.,…Roche, M. (2024b). Promoting resilience in mental health nurses: A partially clustered randomised controlled trial. *International Journal of Nursing Studies, 159*, 1-10. <https://doi.org/doi:https://dx.doi.org/10.1016/j.ijnurstu.2024.104865>

Foster, K., Steele, M., Metcalfe, J., Toomey, N., & Alexander, L. (2024c). Well-being, turnover intention, and stigma attitudes of mental health transition-to-practice nurses: A cross-sectional study. *International Journal of Mental Health Nursing, 33*(2), 409-419. <https://doi.org/doi:https://dx.doi.org/10.1111/inm.13246>

Fradelos, E. C., Papathanasiou, I. V., Dafogianni, C., Misouridou, E., Koutelekos, I., Dousis, E.,…Zartaloudi, A. (2023). The effect of psychological resilience and coping strategies on mental health of nurses. *Advances in Experimental Medicine and Biology, 1425*, 23-30. <https://doi.org/doi:https://dx.doi.org/10.1007/978-3-031-31986-0_3>

Galanis, P., Psomiadi, M. E., Karagkounis, C., Liamopoulou, P., Manomenidis, G., Panayiotou, G., & Bellali, T. (2023). Psychometric properties of the Greek version of the Connor-Davidson Resilience Scale (CD-RISC-10) in a sample of nurses. *International Journal of Environmental Research and Public Health, 20*(18). https://doi.org/doi:https://dx.doi.org/10.3390/ijerph20186752

Galura, S., Warshawsky, N., Hu, W., & Utt, L. (2022). A survey of interim nurse managers to understand the role and the impact on nurse and patient outcomes. *Journal of Nursing Administration*,*52*(1), 42-50. https://doi.org/10.1097/NNA.0000000000001101

Gao, T., Ding, X., Chai, J., Zhang, Z., Zhang, H., Kong, Y., & Mei, S. (2017). The influence of resilience on mental health: The role of general well-being. *International Journal of Nursing Practice*,*23*(3), 1-7. https://doi.org/10.1111/ijn.12535

Garcia-Dia, M. J., O'Flaherty, D., & Arreglado, T. M. (2018). Cultivating resilience in the workplace: Relationship between demographic factors and nurses' perception of resilience. *Journal of Nursing Practice Applications & Reviews of Research*,*8*(1), 6-13. https://doi.org/10.13178/jnparr.2018.0801.0803

Garcia-Izquierdo, M., Pedro, M., Rios-Risquez, M. I., & Sanchez, M. I. S. (2018). Resilience as a moderator of psychological health in situations of chronic stress (burnout) in a sample of hospital nurses. *Journal of Nursing Scholarship*,*50*(2), 228-236. https://doi.org/10.1111/jnu.12367

Georges, M. T., Roberts, L. R., Taylor, E. J., Nick, J. M., & Dehom, S. (2022). Burnout, self-efficacy, and resilience in Haitian nurses: A cross-sectional study. *Journal of Holistic Nursing*,*40*(4), 310-325. https://doi.org/10.1177/08980101211065600

Ghahramani, S., Lankarani, K. B., Marzaleh, M. A., Sayari, M., & Moradi, H. (2023). Resilient Nurses in the COVID-19 compared with non-COVID-19 wards. *Disaster Medicine and Public Health Preparedness*,*17*(1). https://doi.org/10.1017/dmp.2022.264

Gil-Almagro, F., Garcia-Hedrera, F. J., Carmona-Monge, F. J., & Penacoba-Puente, C. (2024). From Anxiety to Hardiness: The role of self-efficacy in Spanish CCU nurses in the COVID-19 pandemic. *Medicina, 60*(2). <https://doi.org/doi:https://dx.doi.org/10.3390/medicina60020215>

Giordano, N. A., Razmpour, O., Mascaro, J. S., Kaplan, D. M., Lewis, A. S., Baird, M.,…Cimiotti, J. P. (2024). Reliability and validity of measures commonly utilized to assess nurse well-being. *Nursing Research, 73*(5), 399-405. <https://doi.org/doi:10.1097/NNR.0000000000000752>

Gotlib, J., Jaworski, M., Cieslak, I., Sobierajski, T., Wawrzuta, D., Malkowski, P., . . . Panczyk, M. (2022). How psychological variables maybe correlated with willingness to get COVID-19 vaccine: A nationwide cross-sectional study of polish novice nurses. *International Journal of Environmental Research and Public Health*, *19*(23). https://doi.org/10.3390/ijerph192315787

Guo, Y., Luo, Y., Lam, L., Cross, W., Plummer, V., & Zhang, J. (2018). Burnout and its association with resilience in nurses: A cross-sectional study. *Journal of Clinical Nursing*,*27*(1), 441-449. https://doi.org/10.1111/jocn.13952

Guo, Y. F., Cross, W., Plummer, V., Lam, L., Luo, Y. H., & Zhang, J. P. (2017). Exploring resilience in Chinese nurses: A cross-sectional study. *Journal of Nursing Management*,*25*(3), 223-230. <https://doi.org/10.1111/jonm.12457>

Gündüz, E. S., Yildirim, N., Akatin, Y., & Gündoğdu, N. A. (2024). Relationship between nurses' resilience and quality of professional life. *International Nursing Review, 71*(4), 1023-1031. https://doi.org/doi:10.1111/inr.12960

Habibpour, Z., Ghorbani-Sani, S., Seyed-Mohammadi, N., Ghader-Jola, K., & Mostafazadeh, A. (2022). Resilience and its relationship with occupational stress and professional quality of life during COVID-19 pandemic. *Psychology, Health and Medicine*,*28*(7), 1977-1984. https://doi.org/10.1080/13548506.2022.2072917

Hale, F. B., Mattheus, D., Fletcher, B., Michel, A., & Fontenot, H. B. (2023). Effects of the COVID 19 pandemic on school nurses' resiliency and ability to cope: A mixed methods study in the state of Hawaii. *Journal of School Nursing*,*39*(2), 133-142. <https://doi.org/10.1177/10598405221124423>

Hamaideh, S. H., Abu Khait, A., Al-Modallal, H., Masa'deh, R., Hamdan-Mansour, A., & Albashtawy, M. (2024). Perceived stress and resilience among psychiatric nurses working in inpatients units. *Hospital Topics*, 1-9. https://doi.org/doi:https://dx.doi.org/10.1080/00185868.2024.2383912

Han, J., Zhang, L., Liu, Y., Zhang, C., Zhang, Y., Tang, R., & Bi, L. (2022). Effect of a group‐based acceptance and commitment therapy programme on the mental health of clinical nurses during the COVID‐19 sporadic outbreak period. *Journal of Nursing Management*,*30*(7), 3005-3012. https://doi.org/10.1111/jonm.13696

Han, S.J., Yeun, Y.-R., & Roh, H. (2023). The impact of resilience on post-traumatic growth among nurses in COVID-19-designated hospitals: The mediating effect of meaning in life. *Healthcare, 11*(21).

Harris, M. D., Williams, L. M., & Duke, R. (2021). The millennial engagement, resiliency and retention study: What does your millennial advanced practice registered nurse workforce really want? *Journal of the American Association of Nurse Practitioners*,*33*(11), 924-930. <https://doi.org/10.1097/JXX.0000000000000535>

Harwood, L., Crandall, J., & LeFuentes, A. (2024). Burnout, resilience and job satisfaction in acute care nurse practitioners. *Nursing Leadership*, *37*(1), 29-51. <https://doi.org/doi:10.12927/cjnl.2024.27357>

Hasan, A., & Alsulami, A. (2024). Mediating role of resilience and its impact on psychological well-being, and mental distress among mental health nurses. *SAGE Open Nursing, 10*. <https://doi.org/doi:https://dx.doi.org/10.1177/23779608231219140>

Hasan, A. A., Asmi, N., & Alsharawneh, A. (2022). Exploring the relationship between burnout, resilience and safety culture for mental health nurses in Saudi Arabia. *British Journal of Mental Health Nursing*,*11*(3). https://doi.org/10.12968/bjmh.2020.0012

He, C., Wu, D., Yang, L., Yang, L., & Yue, Y. (2021). Psychometric properties of the Grit-S in Chinese nurses. *Frontiers in Psychology*,*12*. https://doi.org/10.3389/fpsyg.2021.766055

Hennen, R., & Phillips, K. E. (2023). Emergency nurses' care of psychiatric patients. *Journal of the American Psychiatric Nurses Association*,*29*(2), 96-102. https://doi.org/10.1177/10783903231153418

Honein-AbouHaidar, G., Bou-Hamad, I., Dhaini, S., Davidson, P., Reynolds, N. R., Al-Zaru, I. M., . . . Dumit, N. Y. (2023). The validation of the Arabic version of the resilience scale 14 (RS-14). *BMC Nursing*,*22*(1), 239. https://doi.org/10.1186/s12912-023-01392-9

Hong, Y., Lee, J., Lee, H. J., Kim, K., Cho, I., Ahn, M. H., . . . Chung, S. (2021). Resilience and work-related stress may affect depressive symptoms in nursing professionals during the COVID-19 pandemic era. *Psychiatry Investigation*,*18*(4), 357-363. https://doi.org/10.30773/pi.2021.0019

Hosgor, H., & Yaman, M. (2022). Investigation of the relationship between psychological resilience and job performance in Turkish nurses during the Covid-19 pandemic in terms of descriptive characteristics. *Journal of Nursing Management*,*30*(1), 44-52. https://doi.org/10.1111/jonm.13477

Hou, T., Yin, Q., Xu, Y., Gao, J., Bin, L., Li, H., . . . Ni, C. (2021). The mediating role of perceived social support between resilience and anxiety 1 year after the COVID-19 pandemic: Disparity between high-risk and low-risk nurses in China. *Frontiers in Psychiatry*, *12*. https://doi.org/10.3389/fpsyt.2021.666789

Howie-Esquivel, J., Byon, H. D., Lewis, C., Travis, A., & Cavanagh, C. (2022). Quality of work-life among advanced practice nurses who manage care for patients with heart failure: The effect of resilience during the Covid-19 pandemic. *Heart and Lung*,*55*, 34-41. https://doi.org/10.1016/j.hrtlng.2022.04.005

Hsieh, H. F., Chang, S. C., & Wang, H. H. (2017). The relationships among personality, social support, and resilience of abused nurses at emergency rooms and psychiatric wards in Taiwan. *Women and Health*,*57*(1), 40-51. https://doi.org/10.1080/03630242.2016.1150385

Hsieh, H. F., Chen, Y. M., Wang, H. H., Chang, S. C., & Ma, S. C. (2016a). Association among components of resilience and workplace violence-related depression among emergency department nurses in Taiwan: A cross-sectional study. *Journal of Clinical Nursing*,*25*(17), 2639-2647. https://doi.org/10.1111/jocn.13309

Hsieh, H. F., Hung, Y. T., Wang, H. H., Ma, S. C., & Chang, S. C. (2016b). Factors of resilience in emergency department nurses who have experienced workplace violence in Taiwan. *Journal of Nursing Scholarship*,*48*(1), 23-30. https://doi.org/10.1111/jnu.12177

Hu, D., Kong, Y., Li, W., Han, Q., Zhang, X., Zhu, L. X., . . . Zhu, J. (2020). Frontline nurses' burnout, anxiety, depression, and fear statuses and their associated factors during the COVID-19 outbreak in Wuhan, China: A large-scale cross-sectional study. *EClinicalMedicine*, *24*. https://doi.org/10.1016/j.eclinm.2020.100424

Huang, W., Cai, S., Zhou, Y., Huang, J., Sun, X., Su, Y., . . . Lan, Y. (2021). Personality profiles and personal factors associated with psychological distress in Chinese nurses. *Psychology Research and Behavior Management*,*14*, 1567-1579. https://doi.org/10.2147/PRBM.S329036

Huang, W., Li, L., Zhuo, Y., & Zhang, J. (2023). Analysis of resilience, coping style, anxiety, and depression among rescue nurses on EMTs during the disaster preparedness stage in Sichuan, China: A descriptive cross-sectional survey. *Disaster Medicine and Public Health Preparedness*,*17*(2). https://doi.org/10.1017/dmp.2022.225

Huang, H., Li, F., & Jiang, Y. (2024a). Connor Davidson resilience scores, perceived organizational support and workplace violence among emergency nurses. *International Emergency Nursing, 75*. <https://doi.org/doi:10.1016/j.ienj.2024.101489>

Huang, W., Chen, J., Zheng, L., Shi, G., & Feng, Y. (2024b). Factors affecting nurses' emergency competencies in public health emergency: A cross‐sectional study based on the stress‐coping adaptation model. *Public Health Nursing, 41*(3), 617-625. <https://doi.org/doi:10.1111/phn.13309>

Huang, Y., Li, B., Feng, S., Jiang, S., & Zeng, K. (2024c). Mediating and suppressing effects of coping styles between resilience and empathy for pain in clinical nurses: A cross-sectional study. *Journal of Multidisciplinary Healthcare, 17*, 4653-4667. <https://doi.org/doi:https://dx.doi.org/10.2147/JMDH.S480295>

Hwang, S., & Lee, J. (2023). The influence of COVID-19-related resilience on depression, job stress, sleep quality, and burnout among intensive care unit nurses. *Frontiers in Psychology*, *14*. https://doi.org/10.3389/fpsyg.2023.1168243

Irwin, K. M., Saathoff, A., Janz, D. A., & Long, C. (2021). Resiliency program for new graduate nurses. *Journal for Nurses in Professional Development*,*37*(1), 35-39. https://doi.org/10.1097/NND.0000000000000678

Itzhaki, M., Peles-Bortz, A., Kostistky, H., Barnoy, D., Filshtinsky, V., & Bluvstein, I. (2015). Exposure of mental health nurses to violence associated with job stress, life satisfaction, staff resilience, and post-traumatic growth. *International Journal of Mental Health Nursing*,*24*(5), 403-412. https://doi.org/10.1111/inm.12151

Jamebozorgi, M. H., Karamoozian, A., Bardsiri, T. I., & Sheikhbardsiri, H. (2022). Nurses burnout, resilience, and its association with socio-demographic factors during COVID-19 pandemic. *Frontiers in Psychiatry*,*12*. https://doi.org/10.3389/fpsyt.2021.803506

Janzarik, G., Wollschlager, D., Wessa, M., & Lieb, K. (2022). A group intervention to promote resilience in nursing professionals: A randomised controlled trial. *International Journal of Environmental Research and Public Health*,*19*(2). https://doi.org/10.3390/ijerph19020649

Jeon, M., & Kim, S. (2023). Association between resilience, professional quality of life, and caring behavior in oncology nurses: A cross-sectional Study. *Journal of Korean Academy of Nursing, 53*(6), 597 - 609. https://doi.org/doi:https://dx.doi.org/10.4040/jkan.23058

Jimenez, R., Corral-Liria, I., Trevisson-Redondo, B., Lopez-Lopez, D., Losa-Iglesias, M., & Becerro-de-Bengoa-Vallejo, R. (2022). Burnout, resilience and psychological flexibility in frontline nurses during the acute phase of the COVID-19 pandemic (2020) in Madrid, Spain. *Journal of Nursing Management*,*30*(7), 2549-2556. https://doi.org/10.1111/jonm.13778

Jo, S., Kurt, S., Mayer, K., Pituch, K. A., Simpson, V., Skibiski, J., . . . Reifsnider, E. (2023). Compassion fatigue and COVID-19: A global view from nurses. *Worldviews on Evidence-Based Nursing*,*20*(2), 116-125. https://doi.org/10.1111/wvn.12641

Jose, S., Cyriac, M. C., Dhandapani, M., Mehra, A., & Sharma, N. (2022). Mental health outcomes of perceived stress, anxiety, fear and insomnia, and the resilience among frontline nurses caring for critical COVID-19 patients in intensive care units. *Indian Journal of Critical Care Medicine*,*26*(2), 174-178. https://doi.org/10.5005/jp-journals-10071-24119

Jose, S., Dh, apani, M., & Cyriac, M. C. (2020). Burnout and resilience among frontline nurses during covid-19 pandemic: A cross-sectional study in the emergency department of a tertiary care center, North India. *Indian Journal of Critical Care Medicine*,*24*(11), 1081-1088. https://doi.org/10.5005/jp-journals-10071-23667

Joy, G. V., Alomari, A. M. A., Singh, K., Hassan, N., Mannethodi, K., Kunjavara, J., & AlLenjawi, B. (2023). Nurses' self-esteem, self-compassion and psychological resilience during COVID-19 pandemic. *Nursing Open*,*10*(7), 4404-4412. https://doi.org/10.1002/nop2.1682

Jubin, J., Delmas, P., Gilles, I., Bachmann, A. O., & Bucher, C. O. (2022). Protective factors and coping styles associated with quality of life during the COVID-19 pandemic: A comparison of hospital or care institution and private practice nurses. *International Journal of Environmental Research and Public Health*,*19*(12). https://doi.org/10.3390/ijerph19127112

Jubin, J., Delmas, P., Gilles, I., Oulevey Bachmann, A., & Ortoleva Bucher, C. (2023). Factors protecting Swiss nurses' health during the COVID-19 pandemic: a longitudinal study. *BMC Nursing, 22*(1), 306. <https://doi.org/doi:https://dx.doi.org/10.1186/s12912-023-01468-6>

Jubin, J., Martin, L., Kabwiku, N., Delmas, P., Gilles, I., Oulevey Bachmann, A.,.. Ortoleva Bucher, C. (2024). Protective factors of nurses' mental health and professional wellbeing during the COVID-19 pandemic: A multicenter longitudinal study. *International Journal of Public Health, 69*, 1607449. <https://doi.org/doi:https://dx.doi.org/10.3389/ijph.2024.1607449>

Jurado, M. M. M., Martinez, A. M., Perez-Fuentes, M. C., Lopez, H. C., & Gazquez Linares, J. J. (2022). Job strain and burnout in Spanish nurses during the COVID-19: Resilience as a protective factor in a cross-sectional study. *Human Resources for Health*, *20*(1). https://doi.org/10.1186/s12960-022-00776-3

Kang, H., & Han, K. (2021). Moderating effects of structural empowerment and resilience in the relationship between nurses' workplace bullying and work outcomes: A cross-sectional correlational study. *International Journal of Environmental Research and Public Health*, *18*(4). https://doi.org/10.3390/ijerph18041431

Karabulak, H., & Kaya, F. (2021). The relationship between psychological resilience and stress perception in nurses in Turkey during the COVID-19 pandemic. *Journal of Nursing Research*, *29*(6). https://doi.org/10.1097/jnr.0000000000000454

Kartal, M., Kapikiran, G., & Karakas, N. (2022). The effect of emergency nurses' psychological resilience on their thanatophobic behaviors: A cross-sectional study. *Omega: Journal of Death and Dying*,*38*(1). https://doi.org/10.1177/00302228221128156

Kayalar, A., & Hicdurmaz, D. (2024). Effects of metacognitions, self-compassion, and difficulties in emotion regulation on psychological resilience in oncology nurses. *European Journal of Oncology Nursing, 70*, 102568. https://doi.org/doi:https://dx.doi.org/10.1016/j.ejon.2024.102568

Ke, Y., Zhu, H., Huang, Y., Cheng, L., Wu, X., & Chen, N. (2020). Correlation between monoamine neurotransmitter and cytokine levels and the occurrence of post-traumatic stress disorder among operating room nurses. *Annals of Palliative Medicine*,*9*(6), 3947-3956. https://doi.org/10.21037/apm-20-1829

Kelly, L. A., Gee, P. M., & Butler, R. J. (2021). Impact of nurse burnout on organizational and position turnover. *Nursing Outlook*,*69*(1), 96-102. https://doi.org/10.1016/j.outlook.2020.06.008

Kilinc, T., & Sis Celik, A. (2021). Relationship between the social support and psychological resilience levels perceived by nurses during the COVID-19 pandemic: A study from Turkey. *Perspectives in Psychiatric Care*,*57*(3), 1000-1008. https://doi.org/10.1111/ppc.12648

Kim, J. Y., & Choi, E. H. (2022). Predictors of end-of-life care stress, calling, and resilience on end-of-life care performance: A descriptive correlational study. *BMC Palliative Care*,*21*(1), 71-10. <https://doi.org/10.1186/s12904-022-00961-0>

Kim, M., Jun, J., Lambert, J., Duah, H., Tucker, S. J., O'Mathuna, D. P.,…Fitzpatrick, J. J. (2024). Generational differences in moral injury, resilience, and well-being among nurses: Predictors of intention to leave position and profession. *Western Journal of Nursing Research, 46*(11), 909 EP - 918. <https://doi.org/doi:https://dx.doi.org/10.1177/01939459241287458>

Kim, S., Park, J., Lee, W., & Kim, G. (2024). Internet-based trauma recovery intervention for nurses: A randomized controlled trial. *Complex Psychiatry, 10*(1), 45-58. <https://doi.org/doi:https://dx.doi.org/10.1159/000540350>

Kiratli, D., & Duran, S. (2024). Compassion fatigue and loneliness at work in nurses - the mediating role of resilience: a cross-sectional study. *Journal of Research in Nursing, 29*(4), 290-302. <https://doi.org/doi:https://dx.doi.org/10.1177/17449871241238965>

Kiziloglu, B., & Karabulut, N. (2023). The effect of personality traits of surgical nurses on COVID-19 fear, work stress, and psychological resilience in the pandemic. *Journal of Perianesthesia Nursing*,*38*(4), 572-578. https://doi.org/10.1016/j.jopan.2022.10.006

Kleier, J. A., Lawrence, C., Cavanaugh, G., Schivinski, E., Holl, S., & Bruewer, J. (2022). Professional commitment, resilience and intent to leave the profession among nurses during the COVID-19 pandemic: A descriptive study. *Journal of Nursing Management*,*30*(7), 2577-2584. <https://doi.org/10.1111/jonm.13788>

Kondo, A., Oki, T., & Eckhardt, A. L. (2024). Factors related to resilience and attitudes towards care of the dying among critical care nurses. *Nursing in Critical Care*. <https://doi.org/doi:https://dx.doi.org/10.1111/nicc.13146>

Kong, W., Li, M., Chen, X., & Feng, D. (2024). Subtypes of job satisfaction and health-related quality of life in Chinese male nurses: A latent profile analysis. *Research in Nursing and Health*. <https://doi.org/doi:https://dx.doi.org/10.1002/nur.22421>

Koprowski, K., Meyer, D., Stanfill, T., & Tivis, L. J. (2021). Cultivating joy: Improving nurse resilience through use of a practice playbook. *Applied Nursing Research*,*62*(1), <https://doi.org/10.1016/j.apnr.2021.151484>

Koutantelia, O. M., Togas, C., Alexias, G., & Triantafyllidou, S. (2024). Anxiety, depression, and resilience in pediatric nurses during the COVID-19 pandemic in Greece. *International Journal of Caring Sciences, 17*(3), 1605-1620.

Kutluturkan, S., Sozeri, E., Uysal, N., & Bay, F. (2016). Resilience and burnout status among nurses working in oncology. *Annals of General Psychiatry*,*15*(1). https://doi.org/10.1186/s12991-016-0121-3

Labrague, L. J. (2021). Pandemic fatigue and clinical nurses' mental health, sleep quality and job contentment during the COVID-19 pandemic: The mediating role of resilience. *Journal of Nursing Management*,*29*(7), 1992-2001. https://doi.org/10.1111/jonm.13383

Labrague, L. J., & De los Santos , J. A. A. (2020). COVID-19 anxiety among front-line nurses: Predictive role of organisational support, personal resilience and social support. *Journal of Nursing Management*,*28*(7), 1653-1661. https://doi.org/10.1111/jonm.13121

Labrague, L. J., & De los Santos, J. A. A. (2021a). Prevalence and predictors of coronaphobia among frontline hospital and public health nurses. *Public Health Nursing*,*38*(3), 382-389. https://doi.org/10.1111/phn.12841

Labrague, L. J., & De los Santos, J. A. A. (2021b). Resilience as a mediator between compassion fatigue, nurses' work outcomes, and quality of care during the COVID-19 pandemic. *Applied Nursing Research*,*61*. https://doi.org/10.1016/j.apnr.2021.151476

Labrague, L. J., De los Santos, J. A. A., & Fronda, D. C. (2021c). Perceived COVID-19-associated discrimination, mental health and professional-turnover intention among frontline clinical nurses: The mediating role of resilience. *International Journal of Mental Health Nursing*,*30*(6), 1674-1683. https://doi.org/10.1111/inm.12920

Lan, L., Zhou, M., Wang, L., Chen, X., Dai, M., & Zhang, J. (2023). Enhancing emergency nurses' disaster nursing ability and psychological resilience: A randomized controlled trial. *Emergency Medicine International*. https://doi.org/doi:https://dx.doi.org/10.1155/2023/6108057

Lang, M., Jones, L., Harvey, C., & Munday, J. (2022). Workplace bullying, burnout and resilience amongst perioperative nurses in Australia: A descriptive correlational study. *Journal of Nursing Management*,*30*(6), 1502-1513. https://doi.org/10.1111/jonm.13437

Lara-Cabrera, M. L., Betancort, M., Munoz-Rubilar, C. A., Novo, N. R., & De Las Cuevas, C. (2021). The mediating role of resilience in the relationship between perceived stress and mental health. *International Journal of Environmental Research and Public Health*, *18*. https://doi.org/10.3390/ijerph18189762

Lee, E. Y., Kim, K. J., Ko, S., & Song, E. K. (2022). Communication competence and resilience are modifiable factors for burnout of operating room nurses in South Korea. *BMC Nursing*, *21*(1). <https://doi.org/10.1186/s12912-022-00985-0>

Lee, G. R., Lee, I., Chung, M., & Ha, J. (2023a). Effects of grit, calling, and resilience on the retention intention of general hospital nurses. *International Nursing Review*. <https://doi.org/doi:https://dx.doi.org/10.1111/inr.12908>

Lee, H.-F., Hung, H.-M., & Wang, H.-L. (2024b). Related factors of health-related quality of life in female nurses with in-service training program. *Florence Nightingale Journal of Nursing, 32*(1), 24-29. <https://doi.org/doi:https://dx.doi.org/10.5152/FNJN.2024.23182>

Lee, H. F., Chiang, H. Y., Chang, Y. J., Chang, M. Y., Lee, C. H., Wu, H. C.,…Fetzer, S. (2024a). Effects of resilience and personal accomplishment on the relationship between quality of work life and turnover intention among newly employed nurses. *Journal of Nursing Research*, 10.1097/jnr.0000000000000639. <https://doi.org/doi:https://dx.doi.org/10.1097/jnr.0000000000000639>

Lee, H. F., Hsu, H. C., Efendi, F., Ramoo, V., & Susanti, I. A. (2023b). Burnout, resilience, and empowerment among COVID-19 survivor nurses in Indonesia. *PLoS ONE, 18*(10), e0291073. <https://doi.org/doi:https://dx.doi.org/10.1371/journal.pone.0291073>

Lee, J. Y., & Lee, M. H. (2022). Structural model of retention intention of nurses in small and medium-sized hospitals: Based on Herzberg's Motivation-Hygiene Theory. *Healthcare*, *10*(3). <https://doi.org/10.3390/healthcare10030502>

Lei, J., Lai, H., Zhong, S., Zhu, X., & Lu, D. (2024). The association between intimate partner violence and work thriving/work alienation among Chinese female nurses: The mediating impact of resilience. *Journal of Multidisciplinary Healthcare, 17*, 2741-2754. https://doi.org/doi:https://dx.doi.org/10.2147/JMDH.S461895

Leng, M., Xiu, H., Yu, P., Feng, J., Wei, Y., Cui, Y., . . . Wei, H. (2020). Current state and influencing factors of nurse resilience and perceived job-related stressors. *Journal of Continuing Education in Nursing*,*51*(3), 132-137. https://doi.org/10.3928/00220124-20200216-08

Li, J., Chen, T., Lee, H., & Shih, W. (2021a). The effects of emergency room violence toward nurse's intention to leave-resilience as a mediator. *Healthcare, 9*(5). https://doi.org/10.3390/healthcare9050507

Li, J., Wang, Q., Guan, C., Luo, L., & Hu, X. (2022). Compassion fatigue and compassion satisfaction among Chinese palliative care nurses: A province-wide cross-sectional survey. *Journal of Nursing Management*,*30*(7), 3060-3073. https://doi.org/10.1111/jonm.13708

Li, P., Kuang, H., & Tan, H. (2021b). The occurrence of post-traumatic stress disorder (PTSD), job burnout and its influencing factors among ICU nurses. *American Journal of Translational Research*,*13*(7), 8302-8308. <https://www.ncbi.nlm.nih.gov/pmc/articles/PMC8340215/>

Li, L., Liao, X., & Ni, J. (2024a). A cross-sectional survey on the relationship between workplace psychological violence and empathy among Chinese nurses: the mediation role of resilience. BMC Nursing, 23(1), 85. <https://doi.org/doi:https://dx.doi.org/10.1186/s12912-024-01734-1>

Li, M., Wei, J., Yang, S., Tian, Y., Han, S., Jia, G.,…Wei, B. (2024b). Relationships among perceived social support, mindful self-care, and resilience among a sample of nurses in three provinces in China: a cross-sectional study. *Frontiers in Public Health, 12*, 1334699. <https://doi.org/doi:https://dx.doi.org/10.3389/fpubh.2024.1334699>

Li, S., Wu, Y., Yang, J., Shu, H., Luo, L., & Wei, X. (2024c). Patterns and predictors of resilience in frontline nurses before and after public health emergencies: A latent transition analysis. *Journal of Advanced Nursing*. <https://doi.org/doi:https://dx.doi.org/10.1111/jan.16612>

Li, W., Wan, Z., & XianYu, Y. (2023). Factors influencing nurses self-efficacy two years after the COVID-19 outbreak: A cross-sectional study in Wuhan, China. *Medicine, 102*(36), e35059. <https://doi.org/doi:https://dx.doi.org/10.1097/MD.0000000000035059>

Li, X., Ding, C., Li, G., & Duan, Z. (2024d). Psychosocial characteristics pattern correlated with suicidal ideation and non-suicidal self-injury among nurse staff: a latent profile analysis. *BMC Nursing, 23*(1), 280. <https://doi.org/doi:https://dx.doi.org/10.1186/s12912-024-01970-5>

Li, Y. R., Liu, J. Y., Fang, Y., Shen, X., & Li, S. W. (2024e). Novice nurses' transition shock and professional identity: The chain mediating roles of self-efficacy and resilience. *Journal of Clinical Nursing*, 33(8), 3161-3171. <https://doi.org/doi:https://dx.doi.org/10.1111/jocn.17002>

Liao, J., Ma, X., Gao, B., Zhang, M., Zhang, Y., Liu, M., & Li, X. (2019). Psychological status of nursing survivors in China and its associated factors: 6 years after the 2008 Sichuan earthquake. *Neuropsychiatric Disease and Treatment*,*15*, 2301-2311. <https://doi.org/10.2147/NDT.S203909>

Liao, T., Liu, Y., Luo, W., Duan, Z., Zhan, K., Lu, H., & Chen, X. (2024). Non-linear association of years of experience and burnout among nursing staff: a restricted cubic spline analysis. *Frontiers in Public Health, 12*, 1343293. <https://doi.org/doi:https://dx.doi.org/10.3389/fpubh.2024.1343293>

Liat, H., Inbal, A., & Michal, I. (2024). Nurses during war: Profiles-based risk and protective factors. *Journal of Nursing Scholarship*. <https://doi.org/doi:https://dx.doi.org/10.1111/jnu.13019>

Lin, H., Li, Z., & Yan, M. (2022). Burn-out, emotional labour and psychological resilience among gastroenterology nurses during COVID-19: A cross-sectional study. *BMJ Open*, *12*(12). https://doi.org/10.1136/bmjopen-2022-064909

Lin, L. C., Huang, Y. C., Carter, P., & Zuniga, J. (2021). Resilience among nurses in long term care and rehabilitation settings. *Applied Nursing Research*, *62*. https://doi.org/10.1016/j.apnr.2021.151518

Lin, Y. Y., Lee, Y. H., Chang, S. C., Lee, D. C., Lu, K. Y., Hung, Y. M., & Chang, Y. P. (2019). Individual resilience, intention to stay, and work frustration among postgraduate two-year programme nurses. *Collegian*,*26*(4), 435-440. https://doi.org/10.1016/j.colegn.2018.12.001

Liu, H., Zhou, Z., Liu, Y., Tao, X., Zhan, Y., & Zhang, M. (2023a). Prevalence and associated factors of depression among frontline nurses in Wuhan 6 months after the outbreak of COVID-19: A cross-sectional study. *Medical Science Monitor Basic Research*, *29*. https://doi.org/10.12659/MSMBR.938633

Liu, J., Wei, S., Qiu, G., Li, N., Wang, D., Wu, X., . . . Yi, H. (2023b). Relationship between rumination and post-traumatic growth in mobile cabin hospital nurses: The mediating role of psychological resilience. *Preventive Medicine Reports*, *34*. https://doi.org/10.1016/j.pmedr.2023.102266

Liu, L., Wu, D., Wang, L., Qu, Y., & Wu, H. (2020). Effort-reward imbalance, resilience and perceived organizational support: A moderated mediation model of fatigue in Chinese nurses. *Risk Management and Healthcare Policy*,*13*, 893-901. https://doi.org/10.2147/RMHP.S259339

Liu, X., Ju, X., & Liu, X. (2021). The relationship between resilience and intent to stay among Chinese nurses to support Wuhan in managing COVID-19: The serial mediation effect of post-traumatic growth and perceived professional benefits. *Nursing open*,*8*(5), 2866-2876. <https://doi.org/10.1002/nop2.874>

Liu, J., Yu, X., Kong, L., & Zhou, X. (2023). Prevalence and factors associated with smartphone addiction among nursing postgraduates during the COVID-19 pandemic: A multilevel study from China's mainland. *BMC Psychiatry, 23*(1), 915. <https://doi.org/doi:https://dx.doi.org/10.1186/s12888-023-05369-5>

Liu, L., Lv, Z., Zhou, Y., Liu, M., & Liu, Y. (2023d). The mediating effect of the perceived professional benefit of new nurses in cancer hospitals on the nursing work environment, psychological resilience, and transition shock: A cross-sectional questionnaire survey. *Journal of Nursing Management*, 5741160. <https://doi.org/doi:https://dx.doi.org/10.1155/2023/5741160>

Liu, Q., Jian, X., Peng, F., Wang, M., Li, J., Deng, X.,…Geng, L. (2024a). The effect of alexithymia on distress disclosure among nurses: The mediating role of resilience. Current Psychology: A *Journal for Diverse Perspectives on Diverse Psychological Issues, 43*(25), 21931-21939. <https://doi.org/doi:https://dx.doi.org/10.1007/s12144-024-06004-6>

Liu, Z., Chen, C., Yan, X., Wu, J., & Long, L. (2024b). Analysis of the chain-mediated effects of nurses' sense of professional gain and sense of professional mission between psychological resilience and work engagement in 10 general hospitals in Sichuan province. *Frontiers in Psychology*, 15, 1309901. <https://doi.org/doi:https://dx.doi.org/10.3389/fpsyg.2024.1309901>

Liu, Z., Yan, X., Chen, C., Wu, J., & Lu, J. (2024c). Analysis of the current situation and influencing factors of night shift nurses' sense of occupational benefit. Medicine, 103(46), e40539. <https://doi.org/doi:https://dx.doi.org/10.1097/MD.0000000000040539>

LoGiudice, J. A., & Bartos, S. (2021). Experiences of nurses during the COVID-19 pandemic: A mixed-methods study. *AACN Advanced Critical Care*,*32*(1), 14-25. https://doi.org/10.4037/AACNACC2021816

Lu, J., Xu, P., Ge, J., Zeng, H., Liu, W., & Tang, P. (2023). Analysis of factors affecting psychological resilience of emergency room nurses under public health emergencies. *Inquiry*, *60*. https://doi.org/10.1177/00469580231155296

Luo, D., Song, Y., Cai, X., Li, R., Bai, Y., Chen, B., & Liu, Y. (2022). Nurse managers' burnout and organizational support: The serial mediating role of leadership and resilience. *Journal of Nursing Management*,*30*(8), 4251-4261. https://doi.org/10.1111/jonm.13852

Lyu, H., Yao, M., Zhang, D., & Liu, X. (2020). The relationship among organizational identity, psychological resilience and work engagement of the first-line nurses in the prevention and control of COVID-19 based on structural equation model. *Risk Management and Healthcare Policy*,*13*, 2379-2386. https://doi.org/10.2147/RMHP.S254928

Magtibay, D. L., Chesak, S. S., Coughlin, K., & Sood, A. (2017). Decreasing stress and burnout in nurses: Efficacy of blended learning with stress management and resilience training program. *Journal of Nursing Administration*,*47*(7), 391-395. https://doi.org/10.1097/NNA.0000000000000501

Majrabi, M. A., Hasan, A. A., & Alasmee, N. (2021). Nurses burnout, resilience and its association with safety culture: A cross sectional study. *Mental Health & Social Inclusion*,*25*(2), 171-182. <https://doi.org/10.1108/MHSI-08-2020-0050>

Mallon, A., Mitchell, G., Carter, G., Francis McLaughlin, D., Linden, M., & Brown Wilson, C. (2023). Exploring resilience in care home nurses: An online survey. *Healthcare, 11*(24). https://doi.org/doi:https://dx.doi.org/10.3390/healthcare11243120

Manzano Garcia, G., & Ayala Calvo, J. C. (2012). Emotional exhaustion of nursing staff: Influence of emotional annoyance and resilience. *International Nursing Review*,*59*(1), 101-107. https://doi.org/10.1111/j.1466-7657.2011.00927.x

Mao, L., Huang, L., & Chen, Q. (2021). Promoting resilience and lower stress in nurses and improving inpatient experience through emotional intelligence training in China: A randomized controlled trial. *Nurse Education Today*,*107*. https://doi.org/10.1016/j.nedt.2021.105130

Mao, X., Hou, T., Wang, H., Tang, Y., Ni, C., Zhang, Y.,…Qian, X. (2024). Status and influencing factors of nurses' burnout: A cross-sectional study during COVID-19 regular prevention and control in Jiangsu Province, China. *Global Mental Health, 11*, e54. <https://doi.org/doi:https://dx.doi.org/10.1017/gmh.2024.42>

Mao, X., Luo, P., Li, F., Zhang, F., Zhang, J., Deng, W.,…Dong, W. (2023). PTSD of Chinese nurses in the normalisation of COVID-19 pandemic prevention and control: Prevalence and correlates. *Journal of Global Health, 13*, 06033. https://doi.org/doi:https://dx.doi.org/10.7189/JOGH.13.06033

Martins, V., Serrao, C., Teixeira, A., Castro, L., & Duarte, I. (2022). The mediating role of life satisfaction in the relationship between depression, anxiety, stress and burnout among Portuguese nurses during COVID-19 pandemic. *BMC Nursing*, *21*(1). https://doi.org/10.1186/s12912-022-00958-3

McCoy, T. P., Sauer, P. A., & Sha, S. (2023). Resilience in nurses: The reliability and validity of the resilience scale. *Journal of Nursing Measurement*,*32*(1), 106-116. https://doi.org/10.1891/JNM-2022-0019

Mealer, M., Conrad, D., Evans, J., Jooste, K., Solyntjes, J., Rothbaum, B., & Moss, M. (2014). Feasibility and acceptability of a resilience training program for intensive care unit nurses. *American Journal of Critical Care*,*23*(6), 97-105. https://doi.org/10.4037/ajcc2014747

Mealer, M., Jones, J., Newman, J., McFann, K. K., Rothbaum, B., & Moss, M. (2012). The presence of resilience is associated with a healthier psychological profile in intensive care unit (ICU) nurses: Results of a national survey. *International Journal of Nursing Studies*,*49*(3), 292-299. https://doi.org/10.1016/j.ijnurstu.2011.09.015

Mealer, M., Schmiege, S. J., & Meek, P. (2016). The Connor-Davidson Resilience Scale in critical care nurses: A psychometric analysis. *Journal of Nursing Measurement*,*24*(1), 28-39. <https://doi.org/10.1891/1061-3749.24.1.28>

Mehdizadeh, S., Aghamohammadi, P., Maleki, M., Hasanlo, M., & Abbasi, S. (2024). Relationship between resilience and social and organizational support among nurses working with COVID-19 patients: A cross-sectional study. *Iranian Journal of Nursing and Midwifery Research, 29*(3), 352 EP - 357.

Mei, X. X., Wu, X. N., Wang, H. Y., Wu, J. Y., Wang, X. Q., & Ye, Z. J. (2022). Heterogeneity in psychological resilience and mental health among newly graduated nursing students: A latent profile and generalized additive model analysis. *Psychology Research and Behavior Management*,*15*, 597-606. https://doi.org/10.2147/PRBM.S348661

Meng, Z., Zhang, L., Zan, H., & Wang, J. (2023). Psychological resilience and work engagement of Chinese nurses: a chain mediating model of career identity and quality of work life. *Frontiers in Psychology, 14*, 1275511. <https://doi.org/doi:https://dx.doi.org/10.3389/fpsyg.2023.1275511>

Mensah, J., Nanteer-Oteng, E., & Atinyo, R. (2024). Impact of exposure to workplace sexual harassment on wellbeing among Ghanaian nurses: The role of resilience and conflict resolution climate. *International Journal of Africa Nursing Sciences, 20*, 100694. <https://doi.org/doi:https://dx.doi.org/10.1016/j.ijans.2024.100694>

Mesri, M., Safara, M., Koohestani, H. R., & Baghcheghi, N. (2022). Examining the predictive role of spiritual health and resilience in mental distress of nurses in COVID-19 wards in Iran. *Mental Health, Religion & Culture*,*25*(4), 435-447. https://doi.org/10.1080/13674676.2021.2023487

Meyer, G., & Shatto, B. (2018). Resilience and transition to practice in Direct Entry nursing graduates. *Nurse Education in Practice*,*28*, 276-279. https://doi.org/10.1016/j.nepr.2017.10.008

Mills, J., Woods, C., Harrison, H., Chamberlain-Salaun, J., & Spencer, B. (2017). Retention of early career registered nurses: The influence of self-concept, practice environment and resilience in the first five years post-graduation. *Journal of Research in Nursing*,*22*(5), 372-385. <https://doi.org/10.1177/1744987117709515>

Min, Y.-S., Lee, H.-A., Kwon, S.-C., Lee, I., Kim, K., Kim, J. S.,…Lee, H.-Y. (2023). Occupational and psychological factors associated with burnout in night shift nurses. *Psychiatry Investigation, 20*(10), 904-911. https://doi.org/doi:https://dx.doi.org/10.30773/pi.2023.0084

Mintz-Binder, R., Andersen, S., Sweatt, L., & Song, H. (2021). Exploring strategies to build resiliency in nurses during work hours. *Journal of Nursing Administration*,*51*(4), 185-191. <https://doi.org/10.1097/NNA.0000000000000996>

Mohammad, H. F., Abou Hashish, E. A., & Elliethey, N. S. (2023). The relationship between authentic leadership and nurses' resilience: A mediating role of self-efficacy. *SAGE Open Nursing, 9*. <https://doi.org/doi:https://dx.doi.org/10.1177/23779608231214213>

Moisoglou, I., Katsiroumpa, A., Malliarou, M., Papathanasiou, I. V., Gallos, P., & Galanis, P. (2024). Social support and resilience are protective factors against COVID-19 pandemic burnout and job burnout among nurses in the post-COVID-19 era. *Healthcare, 12*(7). <https://doi.org/doi:https://dx.doi.org/10.3390/healthcare12070710>

Montgomery, A. P., & Patrician, P. A. (2022). Work environment, resilience, burnout, intent to leave during COVID pandemic among nurse leaders: A cross-sectional study. *Journal of Nursing Management*,*30*(8), 4015-4023. https://doi.org/10.1111/jonm.13831

Montgomery, A. P., & Patrician, P. A. (2024). COVID-19 stressors and resilience among nurse leaders. *Nursing Administration Quarterly, 48*(4), E21-E29. https://doi.org/doi:https://dx.doi.org/10.1097/NAQ.0000000000000607

Mousavi, S., Yazdanirad, S., Naeini, M. J., Khoshakhlagh, A., & Haghighat, M. (2023). Determining the effect of selected mental factors on turnover intention through two modulators: Stress and resilience over COVID-19 period. *BMC Health Services Research*, *23*(1). https://doi.org/10.1186/s12913-023-09268-z

Muir, K. J., Webb-Jones, J., Farish, N., Barker, K., Miller-Davis, C., & Galloway, S. (2022). "Room to Reflect": A pilot workplace resiliency intervention for nurses. *International Journal of Environmental Research and Public Health*,*19*(12). https://doi.org/10.3390/ijerph19127272

Nantsupawat, A., Kutney-Lee, A., Abhicharttibutra, K., Wichaikhum, O. A., & Poghosyan, L. (2024). Exploring the relationships between resilience, burnout, work engagement, and intention to leave among nurses in the context of the COVID-19 pandemic: a cross-sectional study. *BMC Nursing, 23*(1), 290. <https://doi.org/doi:https://dx.doi.org/10.1186/s12912-024-01958-1>

Nassar, Y. M., Eshah, N., Al-Maqableh, H. O., Nashwan, A. J., Rayan, A., & Alhawajreh, M. J. (2024). Workplace resilience and compassionate care among Jordanian private sector nurses. *BMC Nursing, 23*(1), 634. <https://doi.org/doi:https://dx.doi.org/10.1186/s12912-024-02295-z>

Nijland, J. W. H. M., Veling, W., Lestestuiver, B. P., & Van Driel, C. M. G. (2021). Virtual reality relaxation for reducing perceived stress of intensive care nurses during the COVID-19 pandemic. *Frontiers in Psychology*,*12*. https://doi.org/10.3389/fpsyg.2021.706527

Nikmanesh, Z., & Khosravi, Z. (2020). The effect of training emotion regulation techniques on resilience and psychological well-being among nurses in Zahedan. *Advances in Nursing & Midwifery*,*29*(2), 1-6. https://doi.org/10.29252/anm-20421

Norful, A. A., Albloushi, M., Zhao, J., Gao, Y., Castro, J., Palaganas, E.,…Rivera, R. (2024). Modifiable work stress factors and psychological health risk among nurses working within 13 countries. *Journal of Nursing Scholarship, 56*(5), 742-751. https://doi.org/doi:https://dx.doi.org/10.1111/jnu.12994

Norouzinia, R., Yarmohammadian, M. H., Ferdosi, M., Masoumi, G., & Ebadi, A. (2022). Development and psychometric evaluation of the emergency nurses' professional resilience tool. *PLoS ONE*,*17*(6). https://doi.org/10.1371/journal.pone.0269539

Nourollahi-Darabad, M., Afshari, D., & Chinisaz, N. (2021). Psychosocial factors associated with resilience among Iranian nurses during COVID-19 outbreak. *Frontiers in Public Health*, *9*. <https://doi.org/10.3389/fpubh.2021.714971>

Öksüz, E., Demiralp, M., Mersin, S., Tüzer, H., Aksu, M., & Sarıkoc, G. (2019). Resilience in nurses in terms of perceived social support, job satisfaction and certain variables. *Journal of Nursing Management*,*27*(2), 423-432. https://doi.org/10.1111/jonm.12703

Ou, X., Chen, Y., Liang, Z., Wen, S., Li, S., & Chen, Y. (2021). Resilience of nurses in isolation wards during the COVID⁃19 pandemic: A cross-sectional study. *Psychology, Health & Medicine*, *26*(1), 98-106. https://doi.org/10.1080/13548506.2020.1861312

Owens, R. A., Houchins, J., Nolan, S., Smalling, M. M., Attia, E., & Fitzpatrick, J. J. (2024). Feasibility of a 3-minute mindful breathing intervention for enhancing psychiatric mental health nurses' resilience during COVID: Findings from a 4-week pilot study. *Holistic Nursing Practice, 38*(1), E1-E9. https://doi.org/doi:https://dx.doi.org/10.1097/HNP.0000000000000628

Ozbek, H., Dilmen, S., & Pinar, S. E. (2022). The relationship between traumatic stress symptoms and psychological resilience in nurses working during the COVID-19 pandemic. *International Journal of Caring Sciences*,*15*(2), 1202-1210. https://doi.org/10.1371/journal.pone.0293392

Pachi, A., Panagiotou, A., Soultanis, N., Ivanidou, M., Manta, M., Sikaras, C.,…Tselebis, A. (2024). Resilience, anger, and insomnia in nurses after the end of the pandemic crisis. *Epidemiologia, 5*(4), 643-657. <https://doi.org/doi:https://dx.doi.org/10.3390/epidemiologia5040045>

Pachi, A., Tselebis, A., Sikaras, C., Sideri, E. P., Ivanidou, M., Baras, S.,…Ilias, I. (2024). Nightmare distress, insomnia and resilience of nursing staff in the post-pandemic era. *AIMS Public Health*, 11(1), 36-57. <https://doi.org/doi:https://dx.doi.org/10.3934/publichealth.2024003>

Pallesen, K. S., McCormack, B., Kjerholt, M., Borre, L. Z., Rosted, E., & Holge-Hazelton, B. (2022). An investigation of the level of burnout and resilience among hospital based nurse managers after COVID 19: A cross-sectional questionnaire-based study. *Journal of Nursing Management*,*30*(8), 4107-4115. https://doi.org/10.1111/jonm.13868

Parizad, N., Soheili, A., Powers, K., Mohebbi, I., Moghbeli, G., & Hosseingolipour, K. (2022). Level of resilience in nurses working at COVID‐19 referral centers in Iran. *Nursing Forum*,*57*(3), 344-351. https://doi.org/10.1111/nuf.12685

Partridge, A., Jorgenson, M., Johnson, E., & Lott, T. (2024). The impact of professional governance on hope, resilience, and empowerment. *The Journal of Nursing Administration, 54*(12), 677-682. https://doi.org/doi:10.1097/NNA.0000000000001512

Park, B., & Jung, J. (2021). Effects of the resilience of nurses in long-term care hospitals during on job stress COVID-19 pandemic: Mediating effects of nursing professionalism. *International Journal of Environmental Research and Public Health*, *18*(19). https://doi.org/10.3390/ijerph181910327

Park, S., & Park, H. J. (2021). The relationships between oncology nurses' attitudes toward a dignified death, compassion competence, resilience, and occupational stress in South Korea. *Seminars in Oncology Nursing*,*37*(3). https://doi.org/10.1016/j.soncn.2021.151147

Pehlivan, T., & Guner, P. (2020). Effect of a compassion fatigue resiliency program on nurses' professional quality of life, perceived stress, resilience: A randomized controlled trial. *Journal of Advanced Nursing*,*76*(12), 3584-3596. https://doi.org/10.1111/jan.14568

Penacoba, C., Catala, P., Velasco, L., Carmona-Monge, F. J., Garcia-Hedrera, F. J., & Gil-Almagro, F. (2021). Stress and quality of life of intensive care nurses during the COVID-19 pandemic: Self-efficacy and resilience as resources. *Nursing in Critical Care*,*26*(6), 493-500. https://doi.org/10.1111/nicc.12690

Peng, J., Luo, H., Ma, Q., Zhong, Y., Yang, X., Huang, Y., . . . Song, Y. (2022). Association between workplace bullying and nurses' professional quality of life: The mediating role of resilience. *Journal of Nursing Management*,*30*(6), 1549-1558. https://doi.org/10.1111/jonm.13471

Phillips, L. A., De los Santos, N., Ntanda, H., & Jackson, J. (2022). The impact of the work environment on the health-related quality of life of Licensed Practical Nurses: A cross-sectional survey in four work environments. *Health and Quality of Life Outcomes*, *20*(1). <https://doi.org/10.1186/s12955-022-01951-9>

Pintus, G., Mannocci, A., Spataro, P., Brai, E., Talucci, M., Capece, G.,…Delli Poggi, A. (2024). Occupational health and psychological wellbeing in first line nurses during health crisis periods: reliability study of the SOIC tool. *La Clinica Terapeutica, 175*(1), 34-41. https://doi.org/doi:https://dx.doi.org/10.7417/CT.2024.5031

Prodromou, M., Stylianou, N., Protopapas, A., & Leontiou, I. (2023). Resilience, burnout and wellbeing of nurses during the third wave of COVID-19 in Cyprus. *Open Nursing Journal*,*17*. https://doi.org/10.2174/18744346-v17-e230704-2023-7

Pu, J., Wang, W., Li, G., Xie, Z., Fan, X., Zhan, N.,…Huang, H. (2024). Psychological resilience and intention to stay among nurses: the mediating role of perceived organizational support. *Frontiers in Psychology*, 15, 1407206. https://doi.org/doi:https://dx.doi.org/10.3389/fpsyg.2024.1407206

Qi, Q., Yan, X., Gao, M., Wu, X., Zhang, S., Dela Rosa, R. D., . . . Xu, Y. (2022). A study on the relationship between mental resilience, work-family conflict, and anxiety of nurses in Shandong, China. *BioMed Research International*, *2022*. https://doi.org/10.1155/2022/4308618

Qin, Y., Liu, J., & Wu, D. (2023). The impact of emotional intelligence on life satisfaction among Chinese nurses: A chain mediating model. *Frontiers in Psychology*, *14*. https://doi.org/10.3389/fpsyg.2023.1125465

Rahmat, I., Pawestri, F., Saputro, R. A., Widianingrum, S., & Hanifah, T. (2023). Psychosocial problems among psychiatric nurses for caring patients with mental disorders during the COVID-19 pandemic. *Nursing Research and Practice*, *2023*. <https://doi.org/10.1155/2023/3689759>

Rashidi, M., Karaman, F., Yildirim, G., Kiskaç N., Ünsal Jafarov, G., & Saygin Şahin, B*.* (2023). Examination of the relationship between thanatophobia and resilience levels of nurses working in intensive care and palliative care units. *BMC Nursing 22*, 281. https://doi.org/10.1186/s12912-023-01405-7

Ren, Y., Zhou, Y., Wang, S., Luo, T., Huang, M., & Zeng, Y. (2018). Exploratory study on resilience and its influencing factors among hospital nurses in Guangzhou, China. *International Journal of Nursing Sciences*,*5*(1), 57-62. https://doi.org/10.1016/j.ijnss.2017.11.001

Reyes, A. T., Fudolig, M., Sharma, M., & L, S. E. (2024). Testing the effectiveness of a mindfulness- and acceptance-based smartphone app for nurses traumatized by the COVID-19 pandemic: A pilot study. *Issues in Mental Health Nursing, 45*(10), 1034-1045. https://doi.org/doi:https://dx.doi.org/10.1080/01612840.2024.2385571

Rhoden, D. J., Colet, C. D. F., & Stumm, E. M. F. (2021). Association and correlation between stress, musculoskeletal pain and resilience in nurses before hospital accreditation maintenance assessment. *Latin American Journal of Nursing*,*29*. https://doi.org/10.1590/1518-8345.4658.3465

Rhoden, D. J., Dezordi, C. C. M., Husein, R. A. M. M., Barbosa, D. A., Treviso, P., Colet, C. D. F., & Stumm, E. M. F. (2021). Nurses' stress and resilience before and after evaluation for hospital accreditation. *Brazilian Journal of Nursing*,*75*(3). https://doi.org/10.1590/0034-7167-2020-1341

Rhéaume, A., & Breau, M. (2022). Antecedents of burnout and turnover intentions during the COVID-19 pandemic in critical care nurses: A mediation study. *Canadian Journal of Critical Care Nursing*,*33*(3), 6-16. https://doi.org/10.5737/23688653-333616

Rivas, N., Lopez, M., Castro, M., Luis-Vian, S., Fernandez-Castro, M., Cao, M., . . . Jimenez, J. (2021). Analysis of burnout syndrome and resilience in nurses throughout the COVID-19 pandemic: A cross-sectional study. *International Journal of Environmental Research and Public Health*, *18*(19). https://doi.org/10.3390/ijerph181910470

Roberts, N. J., McAloney-Kocaman, K., Lippiett, K., Ray, E., Welch, L., & Kelly, C. (2021). Levels of resilience, anxiety and depression in nurses working in respiratory clinical areas during the COVID pandemic. *Respiratory Medicine*,*176*. https://doi.org/10.1016/j.rmed.2020.106219

Roberts, N. J., McAloney-Kocaman, K., Lippiett, K., Ray, E., Welch, L., & Kelly, C. A. (2022). Factors influencing fatigue in UK nurses working in respiratory clinical areas during the second wave of the Covid-19 pandemic: An online survey. *Journal of Clinical Nursing*,*33*(1), 322-332. https://doi.org/10.1111/jocn.16375

Rogers, M., Lamarche, K., Miller, M., Moore, K. S., Spies, L. A., Taylor, J., & Staempfli, S. (2022). Global emotional and spiritual well‐being and resilience of Advanced Practice Nurses during the COVID‐19 pandemic: A cross‐sectional study. *Journal of Advanced Nursing*,*78*(5), 1483-1492. https://doi.org/10.1111/jan.15161

Rosa-Besa, R. D., Graboso, R., Banal, M. S., Malpass, A., & Moyer, G. (2021). Work stress and resiliency in nurse leaders. *Nursing Management*,*52*(7), 42-47. https://doi.org/10.1097/01.NUMA.0000754100.49039.f9

Ruhabadi, F., Assarroudi, A., Mahdavifar, N., & Rad, M. (2022). Correlations of resilience with coping strategies, and the underlying factors in the nurses working in COVID-19 hospitals. *Journal of Education and Health Promotion*,*11*. https://doi.org/10.4103/jehp.jehp_1634_21

Rushton, C. H., Batcheller, J., Schroeder, K., & Donohue, P. (2015). Burnout and resilience among nurses practicing in high-intensity settings. *American Journal of Critical Care*,*24*(5), 412-421. https://doi.org/10.4037/ajcc2015291

Rushton, C. H., Swoboda, S. M., Reimer, T., Boyce, D., & Hanson, G. C. (2023). The mindful ethical practice and resilience academy: Sustainability of impact. *American Journal of Critical Care*, *32*(3), 184-194. https://doi.org/10.4037/ajcc2023236

Rushton, C. H., Swoboda, S. M., Reller, N., Skarupski, K. A., Prizzi, M., Young, P. D., & Hanson, G. C. (2021). Mindful ethical practice and resilience academy: Equipping nurses to address ethical challenges. *American Journal of Critical Care*,*30*(1), 1-11. https://doi.org/10.4037/ajcc2021359

Sacgaca, L., Gonzales, A., Alkubati, S., Alrashidi, N., Alreshidi, M. S., Pasay-An, E., . . . Saguban, R. (2023). The impact of mental well-being, stress, and coping strategies on resilience among staff nurses during COVID-19 in Saudi Arabia: A structural equational model. *Healthcare*, *11*(3). <https://doi.org/10.3390/healthcare11030368>

Saez-Ruiz, I. M., Marquez-Hernandez, V. V., Granados-Gamez, G., Corral-Granados, A., Artero-Lopez, C., & Gutierrez-Puertas, L. (2024). Therapeutic relational communication and resilience among nursing professionals in a pandemic situation. *Nursing Reports, 14*(3), 2130-2139. https://doi.org/doi:https://dx.doi.org/10.3390/nursrep14030159

Salam, H. A., Dumit, N. Y., Clinton, M., & Mahfoud, Z. (2023). Transformational leadership and predictors of resilience among registered nurses: A cross-sectional survey in an underserved area. *BMC Nursing*,*22*(1), 37. https://doi.org/10.1186/s12912-023-01192-1

Sampaio, F., Salgado, R., Antonini, M., Delmas, P., Bachmann, A. O., Gilles, I., & Bucher, C. O. (2022). Workplace wellbeing and quality of life perceived by Portuguese nurses during the COVID-19 Pandemic: The role of protective factors and stressors. *International Journal of Environmental Research and Public Health*, *19*(21). https://doi.org/10.3390/ijerph192114231

Sani, S., Tabrizi, F., Rahmani, A., Sarbakhsh, P., Zamanzadeh, V., & Dickens, G. (2020). Resilience and its relationship with exposure to violence in emergency nurses. *Nursing and Midwifery Studies*,*9*(4), 222-228. https://doi.org/10.4103/nms.nms_6_20

Santos, T. M. D., Balsanelli, A. P., & Souza, K. M. J. d. (2024). Randomized crossover clinical trial of a Mindfulness-based intervention for nurse leaders: A pilot study. Revista Latino-Americana de Enfermagem, 32, e4101. https://doi.org/doi:https://dx.doi.org/10.1590/1518-8345.6548.4101

Sauer, P. A., & McCoy, T. P. (2017). Nurse bullying: Impact on nurses' health. *Western Journal of Nursing Research*,*39*(12), 1533-1546. https://doi.org/10.1177/0193945916681278

Sawalma, A. N., Malak, M. Z., Asfour, B. Y., & Khader, I. A. (2024). The association between psychological reactions, resilience, and work engagement among Palestinian critical care nurses in West Bank. *International Nursing Review*. https://doi.org/doi:https://dx.doi.org/10.1111/inr.12975

Sawyer, A. T., McManus, K., & Bailey, A. K. (2022). A mixed-methods pilot study of a psychoeducational group programme for nurse managers during the COVID-19 pandemic. *Journal of Nursing Management*,*30*(8), 4126-4137. https://doi.org/10.1111/jonm.13881

Sawyer, A. T., Tao, H., & Bailey, A. K. (2023). The impact of a psychoeducational group program on the mental well-being of unit-based nurse leaders: A randomized controlled trial. *International Journal of Environmental Research and Public Health*, *20*(11). <https://doi.org/10.3390/ijerph20116035>

Selvi, E. G., & Yilmaz, G. (2023). Authenticity and psychological resilience levels of nurses and related factors. *Minerva Psychiatry, 64*(3), 335 EP - 346. <https://doi.org/doi:https://dx.doi.org/10.23736/S2724-6612.22.02283-7>

Senturk, E., Ustundag, H., & Demir Gokmen, B. (2024). Melatonin hormone level in nurses and factors affecting it; Investigation according to shift working pattern. *Archives of Psychiatric Nursing, 52*, 52-59. <https://doi.org/doi:https://dx.doi.org/10.1016/j.apnu.2024.07.006>

Sexton, J. R., Truog, A. W., Kelly-Weeder, S., & Loftin, C. (2024). The effects of moral distress on resilience in pediatric emergency department nurses. *Journal of Emergency Nursing, 50*(5), 626-634. <https://doi.org/doi:https://dx.doi.org/10.1016/j.jen.2023.10.006>

Shahrbabaki, P. M., Abolghaseminejad, P., lari, L. A., Zeidabadinejad, S., & Dehghan, M. (2023). The relationship between nurses' psychological resilience and job satisfaction during the COVID-19 pandemic: A descriptive-analytical cross-sectional study in Iran. *BMC Nursing*, *22*(1). <https://doi.org/10.1186/s12912-023-01310-z>

Shen, X.-F., Li, L., Ma, H., Liu, J., Jin, L.-W., Li, X.,…Gao, G. (2023). Influence of resilience on depression among nurses in clean operating departments: The mediating effect of life satisfaction. *World Journal of Psychiatry, 13*(9), 698-706. <https://doi.org/doi:https://dx.doi.org/10.5498/wjp.v13.i9.698>

Shen, Z. M., Wang, Y. Y., Cai, Y. M., Li, A. Q., Zhang, Y. X., Chen, H. J.,…Tan, J. (2024). Thriving at work as a mediator of the relationship between psychological resilience and the work performance of clinical nurses. *BMC Nursing, 23*(1), 194. <https://doi.org/doi:https://dx.doi.org/10.1186/s12912-024-01705-6>

Shi, Y., Guo, H., Zhang, S., Xie, F., Wang, J., Sun, Z., . . . Fan, L. (2018). Impact of workplace incivility against new nurses on job burn-out: A cross-sectional study in China. *BMJ Open*, *8*(4). <https://doi.org/10.1136/bmjopen-2017-020461>

Shi, W., Qiu, C., Zhang, Y., Wang, Y., & Gui, L. (2024). Research on the relationship between nurses' emergency public health response capacity and workplace resilience: A cross-sectional study. International *Journal of Nursing Sciences, 11*(3), 301-307. https://doi.org/doi:https://dx.doi.org/10.1016/j.ijnss.2024.06.007

Shin, N., & Choi, Y. J. (2023). Professional quality of life, resilience, posttraumatic stress and leisure activity among intensive care unit nurses. *International Nursing Review*,*71*(1), 94-100. https://doi.org/10.1111/inr.12850

Siami, S., Gorji, M., & Martin, A. (2023). Psychosocial safety climate and supportive leadership as vital enhancers of personal hope and resilience during the COVID-19 pandemic. *Stress and Health*,*39*(2), 404-413. https://doi.org/10.1002/smi.3192

Sikioti, T., Zartaloudi, A., Pappa, D., Mangoulia, P., Fradelos, E. C., Kourti, F. E.,…Dafogianni, C. (2023). Stress and burnout among Greek critical care nurses during the COVID-19 pandemic. *AIMS Public Health, 10*(4), 755-774. https://doi.org/doi:https://dx.doi.org/10.3934/publichealth.2023051

Soltanian, M., Payegozar, R., Paran, M., & Sharifi, N. (2023). The relationship between metacognitive beliefs with clinical belongingness and resilience among novice nurses in neonatal intensive care units. *Nursing Research and Practice*,*2023*. https://doi.org/10.1155/2023/2949772

Son, D., & Ham, O. (2020). Influence of group resilience on job satisfaction among Korean nurses: A cross-sectional study. *Journal of Clinical Nursing*,*29*(17), 3473-3481. https://doi.org/10.1111/jocn.15385

Stanton, M. P., Houser, R. A., Riechel, M. E. K., Burnham, J. J., & McDougall, G. (2015). The effect of transcranial Direct Current Stimulation (tDCS) on resilience, compassion fatigue, stress and empathy in professional nurses. *Advances in Research*, *5*(2). <https://doi.org/10.9734/AIR/2015/16842>

Suazo Galdames, I., Molero Jurado, M. D. M., Fernandez Martinez, E., Perez-Fuentes, M. D. C., & Gazquez Linares, J. J. (2024). Resilience, burnout and mental health in nurses: A latent mediation model. *Journal of Clinical Medicine, 13*(10). https://doi.org/doi:https://dx.doi.org/10.3390/jcm13102769

Sukut, O., Sahin‐Bayindir, G., Ayhan‐Balik, C. H., & Albal, E. (2022). Professional quality of life and psychological resilience among psychiatric nurses. *Perspectives in Psychiatric Care*,*58*(1), 330-338. https://doi.org/10.1111/ppc.12791

Sullivan, C. E., King, A., Holdiness, J., Durrell, J., Roberts, K. K., Spencer, C., . . . Mandrell, B. N. (2019). Reducing compassion fatigue in inpatient pediatric oncology nurses. *Oncology Nursing Forum*, *46*(3), 338-347. https://doi.org/10.1188/19.ONF.338-347

Sun, T., Zhang, S., Yin, H., Li, Q., Li, Y., Li, L., . . . Liu, B. (2022). Can resilience promote calling among Chinese nurses in intensive care units during the COVID-19 pandemic? The mediating role of thriving at work and moderating role of ethical leadership. *Frontiers in Psychology*, *13*. https://doi.org/10.3389/fpsyg.2022.847536

Ta'an, W., Hijazi, D., Suliman, M., Al Rub, R. A., & Albashtawy, M. (2024). Exploring the relationships between stress, resilience and job performance among nurses in Jordan. *Nursing Management, 31*(3). https://doi.org/doi:https://dx.doi.org/10.7748/nm.2023.e2112

Tabakakis, C., McAllister, M., Bradshaw, J., & To, Q. G. (2019). Psychological resilience in New Zealand registered nurses: The role of workplace characteristics. *Journal of Nursing Management*,*27*(7), 1351-1358. https://doi.org/10.1111/jonm.12815

Tahghighi, M., Brown, J. A., Breen, L. J., Kane, R., Hegney, D., & Rees, C. S. (2019). A comparison of nurse shift workers' and non-shift workers' psychological functioning and resilience. *Journal of Advanced Nursing*,*75*(11), 2570-2578. https://doi.org/doi:https://dx.doi.org/10.1111/jan.14023

Talebian, F., Hosseinnataj, A., & Yaghoubi, T. (2022). The relationship between resilience and moral distress among Iranian critical care nurses: A cross-sectional correlational study. *Ethiopian Journal of Health Sciences*,*32*(2), 405-412. https://doi.org/10.4314/ejhs.v32i2.21

Tang, Y., He, C., Feng, L., Wu, D., Zhou, X., Li, T., . . . Yue, Y. (2022). The impact of implicit theories on resilience among Chinese nurses: The chain mediating effect of grit and meaning in life. *Frontiers in Psychology*,*13*. https://doi.org/10.3389/fpsyg.2022.940138

Tang, L., Wang, F., & Tang, T. (2024). Exploring the relationship between family care, organizational support, and resilience on the professional quality of life among emergency nurses: A cross-sectional study. *International Emergency Nursing, 72*, 101399. https://doi.org/doi:https://dx.doi.org/10.1016/j.ienj.2023.101399

Tseng, H. M., Shih, W. M., Shen, Y. C., Ho, L. H., & Wu, C. F. (2018). Work stress, resilience, and professional quality of life among nurses caring for mass burn casualty patients after formosa color dust explosion. *Journal of Burn Care and Research*,*39*(5), 798-804. https://doi.org/10.1093/jbcr/irx053

Tsouvelas, G., Kalaitzaki, A., Tamiolaki, A., Rovithis, M., & Konstantakopoulos, G. (2022). Secondary traumatic stress and dissociative coping strategies in nurses during the COVID-19 pandemic: The protective role of resilience. *Archives of Psychiatric Nursing*,*41*, 264-270. https://doi.org/10.1016/j.apnu.2022.08.010

Turan, N. (2021). An investigation of the effects of an anger management psychoeducation programme on psychological resilience and affect of intensive care nurses. *Intensive and Critical Care Nursing*,*62*. https://doi.org/10.1016/j.iccn.2020.102915

Turan, N., & Canbulat, S. (2023). The effectiveness of the training program on accepting and expressing emotions on the psychological resilience and depression levels of nurses: A two-year follow-up study. *Archives of Psychiatric Nursing*,*44*, 1-7. <https://doi.org/10.1016/j.apnu.2023.03.002>

Turunc, O., Caliskan, A., Akkoc, I., Koroglu, O., Gursel, G., Demirci, A.,…Ozcanarslan, N. (2024). The impact of intensive care unit nurses' burnout levels on turnover intention and the mediating role of psychological resilience. *Behavioral Sciences, 14*(9). https://doi.org/doi:https://dx.doi.org/10.3390/bs14090782

Uzar-ozcetin, Y. S., Sarioglu, G., & Dursun, S. I. (2019). Resilience, burnout and psychological well-being levels of oncology nurses. *Current Approaches in Psychiatry*,*11*, 147-164. <https://doi.org/10.18863/PGY.589202>

Uzar-Ozcetin, Y. S., & Budak, S. E. (2024). The relationship between attitudes toward death, rumination, and psychological resilience of oncology nurses. *Seminars in Oncology Nursing, 40*(3), 151645. https://doi.org/doi:https://dx.doi.org/10.1016/j.soncn.2024.151645

Villa, M., Balice-Bourgois, C., Tolotti, A., Falco-Pegueroles, A., Barello, S., Luca, E. C., . . . Bonetti, L. (2021). Ethical conflict and its psychological correlates among hospital nurses in the pandemic: A cross-sectional study within Swiss COVID-19 and non-COVID-19 wards. *International Journal of Environmental Research and Public Health*, *18*(22). <https://doi.org/10.3390/ijerph182212012>

Vogt, K. S., Johnson, J., Coleman, R., Simms-Ellis, R., Harrison, R., Shearman, N.,…Grange, A. (2024). Can the Reboot coaching programme support critical care nurses in coping with stressful clinical events? A mixed-methods evaluation assessing resilience, burnout, depression and turnover intentions. *BMC Health Services Research, 24*(1), 343. https://doi.org/doi:https://dx.doi.org/10.1186/s12913-023-10468-w

Walpita, Y. N., & Arambepola, C. (2020). High resilience leads to better work performance in nurses: Evidence from South Asia. *Journal of Nursing Management*,*28*(2), 342-350. https://doi.org/10.1111/jonm.12930

Walpita, Y. N., & Arambepola, C. (2022). Assessment of resilience levels among Sri Lankan nurses: Is there room for improvement? *International Journal of Health Planning and Management*, *37*, 3238-3249. https://doi.org/10.1002/hpm.3552

Wang, H., & Dela Rosa, R. D. (2022). Situational analysis of influencing factors of the psychological resilience of junior nurses at a tertiary hospital in Linfen City, Shanxi Province, China. *Africa Journal of Nursing & Midwifery*,*24*(3), 1-14. https://doi.org/10.25159/2520-5293/12200

Wang, L., Tao, H., Bowers, B. J., Brown, R., & Zhang, Y. (2018). Influence of social support and self-efficacy on resilience of early career registered nurses. *Western Journal of Nursing Research*, *40*(5), 648-664. https://doi.org/10.1177/0193945916685712

Wang, Y., Li, L., Tan, S., Guan, Y., & Luo, X. (2023). Psychological stress and associated factors in caring for patients with delirium among intensive care unit nurses: A cross-sectional study. *Australian Critical Care*,*36*, 793-798. https://doi.org/10.1016/j.aucc.2022.09.006

Wang, Z., Liu, H., Huang, J., Li, S., Yan, Z., & Luan, X. (2022). Validation of a Chinese version of the Analysing and Developing Adaptability and Performance in Teams to Enhance Resilience Scale in nurses in China. *Journal of Nursing Management*,*30*(5), 1324-1336. <https://doi.org/10.1111/jonm.13628>

Wang, L., Li, S., Liu, X., Li, R., & Li, R. (2024a). The mediating role of resilience in the relationship between meaning in life and attitude toward death among ICU nurses: a cross-sectional study. *Frontiers in Psychology, 15*, 1414989. <https://doi.org/doi:https://dx.doi.org/10.3389/fpsyg.2024.1414989>

Wang, Q., Luan, Y., Liu, D., Dai, J., Wang, H., Zhang, Y.,…Bi, H. (2024b). Guided self-help mindfulness-based intervention for increasing psychological resilience and reducing job burnout in psychiatric nurses: A randomized controlled trial. *International Journal of Nursing Practice, 30*(4), e13204. <https://doi.org/doi:https://dx.doi.org/10.1111/ijn.13204>

Wang, W., Ye, J., Wei, Y., Yuan, L., Wu, J., Xia, Z.,…Xiao, A. (2024c). Characteristics and influencing factors of post-traumatic growth: A cross-sectional study of psychiatric nurses suffering from workplace violence in Guangdong China. *Journal of Multidisciplinary Healthcare, 17*, 1291-1302. <https://doi.org/doi:https://dx.doi.org/10.2147/JMDH.S450347>

Wang, X., Xue, J., Zhang, A., Luo, Y., Chen, O., Liu, C.,…Wu, M. (2024d). Job satisfaction and psychological factors influence the caring behavior in nurses in Sierra Leone: a cross-sectional study. *Frontiers in Psychology, 15*, 1418260. <https://doi.org/doi:https://dx.doi.org/10.3389/fpsyg.2024.1418260>

Waterworth, S., & Grace, A. M. (2021). Resilience and burnout in pediatric nurses in a tertiary children's hospital. *The American Journal of Maternal/Child Nursing*,*46*(3), 168-173. https://doi.org/10.1097/NMC.0000000000000713

Wei, W., & Taormina, R. J. (2014). A new multidimensional measure of personal resilience and its use: Chinese nurse resilience, organizational socialization and career success. *Nursing Inquiry*, *21*(4), 346-357. https://doi.org/doi:10.1111/nin.12067

Welden, L. M. S., Chen, C., Willegal-Russ, K., & Kalb, E. (2023). Nurse resiliency and health in practicing nurses before and during COVID-19. *Journal of Nursing Administration*,*53*(7), 420-428. https://doi.org/10.1097/NNA.0000000000001308

Welden, L. M. S., Kalb, E., Willegal, K., Chen, C., & White, A. (2021). A descriptive study of resiliency and health in practicing nurses. *Journal of Nursing Administration*,*51*(7), 366-373. https://doi.org/10.1097/NNA.0000000000001031

Williams, J., Hadjistavropoulos, T., Ghandehari, O. O., Malloy, D. C., Hunter, P. V., & Martin, R. R. (2016). Resilience and organisational empowerment among long-term care nurses: Effects on patient care and absenteeism. *Journal of Nursing Management*,*24*(3), 300-308. <https://doi.org/doi:10.1111/jonm.12311>

Wu, X., Tang, L., & Gong, J. (2024). Correlation analysis of mental toughness, family social support, and anxiety of nursing staff. *American Journal of Translational Research, 1*6(6), 2563-2570. https://doi.org/doi:https://dx.doi.org/10.62347/PWLM8459

Xia, W., Defang, W., Xiaoli, G., Jinrui, C., Weidi, W., Junya, L., . . . Hui, W. (2022). Compassion satisfaction and compassion fatigue in frontline nurses during the COVID-19 pandemic in Wuhan, China. *Journal of Nursing Management*,*30*(7), 2537-2548. https://doi.org/10.1111/jonm.13777

Xiaoyi, C., & Lin, C. (2021). The impact of resilience on turnover intention in dialysis nurses: The mediating effects of work engagement and compassion fatigue. *Japan Journal of Nursing Science*,*18*(3), 1-11. https://doi.org/10.1111/jjns.12414

Xu, D., Zhang, N., Bu, X., & Xu, Z. (2024). A latent profile analysis of psychological resilience associated with work fatigue among Chinese nurses. *Research in Nursing and Health*. https://doi.org/doi:https://dx.doi.org/10.1002/nur.22371

Xue, H., Si, X., Wang, H., Song, X., Zhu, K., Liu, X., & Zhang, F. (2022). Psychological resilience and career success of female nurses in central China: The mediating role of craftsmanship. *Frontiers in psychology*, *13*. https://doi.org/10.3389/fpsyg.2022.915479

Yan, J., Wu, C., Du, Y., He, S., Shang, L., & Lang, H. (2022a). Occupational stress and the quality of life of nurses in infectious disease departments in China: The mediating role of psychological resilience. *Frontiers in psychology*,*13*. https://doi.org/10.3389/fpsyg.2022.817639

Yan, J., Wu, C., He, C., Lin, Y., He, S., Du, Y., . . . Lang, H. (2022b). The social support, psychological resilience and quality of life of nurses in infectious disease departments in China: A mediated model. *Journal of Nursing Management*,*30*(8), 4503-4513. https://doi.org/10.1111/jonm.13889

Yan, J., Wu, C., Liu, Y., Zhang, H., He, C., Lin, Y., . . . Lang, H. (2023). Influencing factors of quality of life among front-line nurses who collected nucleic acid samples during COVID-19: A path analysis. *Frontiers in Public Health*,*11*. https://doi.org/10.3389/fpubh.2023.1154725

Yan, X., Jia, X., Feng, L., Ge, W., Kong, B., & Xia, M. (2024). Enhancing nursing competence in China: The interplay of resilience, mindfulness and social support. *Nurse Education in Practice, 79*, 104087. https://doi.org/doi:https://dx.doi.org/10.1016/j.nepr.2024.104087

Yang, G., Liu, J., Liu, L., Wu, X., Ding, S., & Xie, J. (2018). Burnout and resilience among transplant nurses in 22 hospitals in China. *Transplantation Proceedings*,*50*(10), 2905-2910. https://doi.org/10.1016/j.transproceed.2018.04.033

Yang, R., Gao, Y., & Ji, Z. (2023a). The relationship between self-regulated learning, mindful agency, and psychological resilience in Chinese master of nursing specialists: A cross-sectional study. *Frontiers in psychology*,*14*. https://doi.org/10.3389/fpsyg.2023.1066806

Yang, R., Ke, Q., Chan, S. W., Liu, Y., Lin, H., Li, W., & Zhu, J. (2022). A cross-sectional examination of the relationship between nurses' experiences of skin lesions and anxiety and depression during the COVID-19 pandemic: Exploring the mediating role of fear and resilience. *Journal of Nursing Management*,*30*(6), 1903-1912. https://doi.org/10.1111/jonm.13638

Yang, Q., Zheng, Z., Ge, L., Huang, B. X., Liu, J., Wang, J.,…Zhang, J. (2023). The impact of resilience on clinical nurses' moral courage during COVID-19: A moderated mediation model of ethical climate and moral distress. *International Nursing Review*. <https://doi.org/doi:https://dx.doi.org/10.1111/inr.12871>

Yao, X., Wang, J., Yang, Y., & Zhang, H. (2023). Factors influencing nurses' post-traumatic growth during the COVID-19 pandemic: Bayesian network analysis. Frontiers in psychiatry, 14, 1163956. <https://doi.org/doi:https://dx.doi.org/10.3389/fpsyt.2023.1163956>

yazdanirad, S., haghighat, M., Jahadinaeini, M., khoshakhlagh, A., & mousavi, S. (2024). A Bayesian approach to reveal the role of psychological factors on turnover intention among nurses during the COVID-19 pandemic. *BMC Health Services Research, 24*(1), 877. <https://doi.org/doi:https://dx.doi.org/10.1186/s12913-024-11307-2>

Yeh, Y. C., Sheng, W. H., Pan, M. Y., Liu, H. Y., Liu, C. Y., & Chiou, P. Y. (2024). Stress coping and resilience of frontline nurses under the emergency infectious disease pandemic: A latent class analysis. *Applied Nursing Research, 80*, 151857. <https://doi.org/doi:https://dx.doi.org/10.1016/j.apnr.2024.151857>

Yi, R., Zhou, Z., Ma, W., Yang, C., Wang, F., & Wu, J. (2023). Mediating role of psychological resilience in the relationship between self-efficacy and professional identity among nurses. *Biotechnology & Genetic Engineering Reviews*,*39*(1), 1-13. <https://doi.org/10.1080/02648725.2023.2190943>

Yildirim, D., Senyuva, E., & Kaya, E. (2024). Turkish nurses' psychological resilience and burnout levels during the COVID-19 pandemic: A correlational study. I*nternational Journal of Nursing Practice, 30(*4), e13239. https://doi.org/doi:https://dx.doi.org/10.1111/ijn.13239

Ying, L. Y., Ramoo, V., Ling, L. W., Nahasaram, S. T., Lei, C. P., Leong, L. K., & Danaee, M. (2021). Nursing practice environment, resilience, and intention to leave among critical care nurses. *Nursing in Critical Care*,*26*(6), 432-440. https://doi.org/10.1111/nicc.12551

Yousefzadeh, N. K., Dehkordi, M. K., Vahedi, M., Astaneh, A. N., & Bateni, F. S. (2024). The effectiveness of Balint group work on the quality of work life, resilience, and nurse-patient communication skills among psychiatric nurses: a randomized controlled trial. *Frontiers in Psychology, 15,* 1212200.

Yu, F., Cavadino, A., Mackay, L., Ward, K., King, A., & Smith, M. (2020). Physical activity and personal factors associated with nurse resilience in intensive care units. *Journal of Clinical Nursing*,*29*(17), 3246-3262. https://doi.org/10.1111/jocn.15338

Yu, H., Huang, C., Chin, Y., Shen, Y., Chiang, Y., Chang, C., & Lou, J. (2021). The mediating effects of nursing professional commitment on the relationship between social support, resilience, and intention to stay among newly graduated male nurses: A cross-sectional questionnaire survey. *International Journal of Environmental Research and Public Health*, *18*(14). https://doi.org/10.3390/ijerph18147546

Yu, J., Song, Y., Hua, D., Shi, Y., & Zhao, J. (2022). Factors influencing mindfulness among clinical nurses in China: An observational cross-sectional study. *Journal of Nursing Management*, *30*(3), 758-766. https://doi.org/10.1111/jonm.13560

Yu, M., & Lee, H. (2018). Impact of resilience and job involvement on turnover intention of new graduate nurses using structural equation modelling. *Japan Journal of Nursing Science*,*15*(4), 351-362. https://doi.org/10.1111/jjns.12210

Yu-Chin, C., Forster, A. K., Zhang, L., & Foli, K. J. (2023). Nurses' psychological trauma and cognitive control in the COVID-19 pandemic. *SAGE Open Nursing, 9*. https://doi.org/doi:https://dx.doi.org/10.1177/23779608231214601

Yun, Z., Zhou, P., & Zhang, B. (2022). High-performance work systems, thriving at work, and job burnout among nurses in Chinese public hospitals: The role of resilience at work. *Healthcare*, *10*(10). https://doi.org/10.3390/healthcare10101935

Yusefi, A. R., Daneshi, S., Davarani, E. R., Nikmanesh, P., Mehralian, G., & Bastani, P. (2021). Resilience level and its relationship with hypochondriasis in nurses working in COVID-19 reference hospitals. *BMC Nursing*,*20*(1), 1-9. https://doi.org/10.1186/s12912-021-00730-z

Zahednezhad, H., Zareiyan, A., & Jame, S. Z. B. (2021). Relationship between quality of work-life, resilience and burnout among nursing professionals during COVID-19 pandemic in Iran: A cross-sectional study. *Belitung Nursing Journal*,*7*(6), 508-515. https://doi.org/10.33546/bnj.1702

Zakeri, M. A., Rafsanjanipoor, S. M. H., Zakeri, M., & Dehghan, M. (2021). The relationship between frontline nurses' psychosocial status, satisfaction with life and resilience during the prevalence of COVID-19 disease. *Nursing Open*,*8*(4), 1829-1839. <https://doi.org/10.1002/nop2.832>

Zeng, D., Li, Y., Yang, C., Tang, D., Yi, Y., He, Y., & Yang, M. (2024). Prolonged exposure to a public health event: the mediating role of resilience between social support and post-traumatic growth among frontline nurses. BMC Nursing, 23(1), 753.

Zhan, N., Xu, Y., Pu, J., Wang, W., Xie, Z., & Huang, H. (2024). The interaction between mental resilience and insomnia disorder on negative emotions in nurses in Guangdong Province, China. *Frontiers in Psychiatry, 15*, 1396417. https://doi.org/doi:https://dx.doi.org/10.3389/fpsyt.2024.1396417

Zhang, J., Wang, X., Xu, T., Li, J., Li, H., Wu, Y., . . . Zhang, J. (2021a). The effect of resilience and self-efficacy on nurses' compassion fatigue: A cross-sectional study. *Journal of Advanced Nursing*, *78*(7), 2030-2041. https://doi.org/10.1111/jan.15113

Zhang, X., Jiang, X., Ni, P., Li, H., Li, C., Zhou, Q., . . . Cao, J. (2021b). Association between resilience and burnout of front‐line nurses at the peak of the COVID‐19 pandemic: Positive and negative affect as mediators in Wuhan. *International Journal of Mental Health Nursing*, *30*(4), 939-954. https://doi.org/10.1111/inm.12847

Zhang, Y., Guan, C., Jiang, J., Zhu, C., & Hu, X. (2023). Mediating effect of resilience on the relationship between perceived social support and burnout among Chinese palliative nurses. *Journal of Clinical Nursing*,*32*(13), 3887-3897. https://doi.org/10.1111/jocn.16532

Zhang, Y., Jiang, J., Zhu, C., Liu, C., Guan, C., & Hu, X. (2022a). Status and related factors of burnout among palliative nurses in China: A cross-sectional study. *BMC Nursing*,*21*(1). https://doi.org/10.1186/s12912-022-01083-x

Zhang, Y., Xiong, Y., Zhang, L., Jiang, X., Zhuang, X., Meng, L., . . . Wu, J. (2022b). Sociodemographic and psychological predictors of resilience among frontline nurses fighting the COVID-19 pandemic. *Disaster Medicine and Public Health Preparedness*, *17*. <https://doi.org/10.1017/dmp.2022.138>

Zhang, H. L., Wu, C., Hu, M. Y., Ma, W. J., Xu, X. L., Shi, R. J., & Lang, H. J. (2024a). Risk perception and quality of working life of nurses in infectious disease department in China: The chain-mediating effects of psychological resilience and social support. *Nursing Open, 11*(9), e70045. <https://doi.org/doi:https://dx.doi.org/10.1002/nop2.70045>

Zhang, L., Liang, X., Cheng, N., Han, L., Jia, Y., Wang, R.,…Jiang, X. (2024b). Psychological resilience mediates sense of professional mission and career success in Chinese intensive care unit nurses: a cross-sectional study. *BMC Nursing, 23*(1), 607. <https://doi.org/doi:https://dx.doi.org/10.1186/s12912-024-02271-7>

Zhang, M., Chi, C., Liu, Q., Zhou, N., Zhou, Z., Tao, X.,…Liu, H. (2024c). Prevalence and associated factors of insomnia symptoms after ending China's dynamic zero-COVID policy: a cross-sectional survey of frontline nursing staff in Chinese hospitals. *Frontiers in Public Health, 12*, 1363048. <https://doi.org/doi:https://dx.doi.org/10.3389/fpubh.2024.1363048>

Zhang, X., Tian, W., Tang, X., Jia, L., Meng, X., Shi, T., & Zhao, J. (2024d). Mediating role of resilience on burnout to well-being for hospital nursing staff in Northeast China: a cross-sectional study. BMJ open, 14(11), e081718. <https://doi.org/doi:https://dx.doi.org/10.1136/bmjopen-2023-081718>

Zhang, Y., Min, S., & Hao, H. (2024e). Resilience's role in clinical belongingness and presenteeism of new nurses: A moderated mediation model. *Nursing & Health Sciences, 26*(1), 1-7. <https://doi.org/doi:10.1111/nhs.13078>

Zhao, W., Shu, T., Ma, Y., Wei, X., Zhu, C., Peng, L., . . . Zhang, Q. (2022). Examining the dimensionality, reliability, and invariance of the Chinese version of the Resilience Scale-14: A multicenter cross-sectional survey in Chinese junior nurses. *Frontiers in Psychiatry*,*13*. https://doi.org/10.3389/fpsyt.2022.964151

Zhao, Y., & Hu, J. (2023). Psychological distress in emergency department nurses at women's and children's hospitals during the COVID-19 epidemic: A cross-sectional study. *Medical Science Monitor*,*29*. https://doi.org/10.12659/MSM.940175

Zhao, Y., Wang, H., Sun, D., Ma, D., Li, H., Li, Y., . . . Sun, J. (2021). Job satisfaction, resilience and social support in relation to nurses' turnover intention based on the theory of planned behaviour: A structural equation modelling approach. *International Journal of Nursing Practice*,*27*(6). https://doi.org/10.1111/ijn.12941

Zheng, Z., Zhan, S., Xu, J., Lu, K., Wang, J., Huang, B.,…Ge, L. (2023). The impact of nurses' experiences of hospital violence on resilience: A mediated moderation model. *Journal of Clinical Nursing*. <https://doi.org/doi:https://dx.doi.org/10.1111/jocn.16982>

Zhou, Y., Gao, W., Li, H., Yao, X., Wang, J., & Zhao, X. (2024). Network analysis of resilience, anxiety and depression in clinical nurses. *BMC Psychiatry, 24*(1), 719. https://doi.org/doi:https://dx.doi.org/10.1186/s12888-024-06138-8
