# Supplementary material for: A Scoping Review of Instruments Used to Measure Resilience in Samples of Nurses
Source: J Adv Nurs. 2025 Feb 7;81(9):5718–62. doi: 10.1111/jan.16769 (PMC12371848; doi:10.1111/jan.16769)
Supplement: Supplementary file 4 — File S4. [file JAN-81-5718-s003.docx]

**Supplementary File 4.** Instrument reliability and validation

| **Instrument** | **Reported reliability and validation** | **Cronbach’s alpha** |
| --- | --- | --- |
| Adapted Adult Personal Resilience Scale (Handoyo et al., 2021) | Content validation and reliability testing | 0.809-0.960 |
| Brief Resilience Scale (Smith et al., 2008) | Convergent validity, discriminant predictive validity and reliability | 0.80-0.91 |
| Brief Resilient Coping Scale (Sinclair & Wallston, 2004) | Reliability, test-retest reliability, initial validity, predictive validity, sensitivity to change, and interaction effects | 0.69 |
| Connor-Davidson Resilience Scale 2-item (Vaishnavi et al., 2007) | Internal consistency, test–retest reliability, convergent validity, and divergent validity. | Not reported |
| Connor-Davidson Resilience Scale 10-item (Campbell‐Sills & Stein, 2007) | Construct validity and internal consistency | 0.85 |
| Connor-Davidson Resilience Scale 25-item (Connor & Davidson, 2003) | Test-retest reliability, internal consistency, convergent validity, and divergent validity | 0.89 |
| Emergency Nurse' Professional Resilience Tool (Norouzinia et al., 2022) | Face validity, content validity, construct validity, consistency, stability, internal consistency, and repeatability | 0.915 |
| Personal Resilience Tool (Wei & Taormina, 2014) | Concurrent validity and internal consistency | 0.74-0.77 |
| Resilience at Work Scale (Winwood et al., 2013) | Internal consistency, face validity, and ecological validity | 0.60-0.89 |
| Resilience at Work Team Scale (McEwen & Boyd, 2018) | Internal reliability and face validity | 0.83-0.98 |
| Response to Stressful Experiences Scale (Johnson et al., 2011) | Internal consistency, test-retest reliability, convergent validity, discriminant validity, concurrent validity, and incremental criterion validity | 0.91-0.93 |
| Resilience Scale 14-item (Wagnild, 2009) | Internal consistency and construct validity | 0.89-0.96 |
| Resilience Scale 25-item (Wagnild & Young, 1993) | Internal consistency, test-retest reliability, construct validity, and concurrent validity | 0.91 |
| Resilience Scale for Adults (Friborg et al., 2003) | Internal consistency, test-retest reliability, construct validity, discriminant validity, convergent validity, | 0.67-0.90 |
| Workplace Resiliency Inventory (McLarnon & Rothstein, 2013) | Internal consistency, criterion validity, convergent validity and content validity, | 0.76-0.96 |
